# Applications, indications, and effects of passive hydrotherapy WATSU (WaterShiatsu)—A systematic review and meta-analysis

**DOI:** 10.1371/journal.pone.0229705

**Published:** 2020-03-13

**Authors:** Agnes M. Schitter, Johannes Fleckenstein, Peter Frei, Jan Taeymans, Nico Kurpiers, Lorenz Radlinger

**Affiliations:** 1 Institute of Complementary Medicine, University of Bern, Switzerland; 2 Institute of Sport Science, University of Hildesheim, Germany; 3 Department of Sports Medicine, Institute of Sports Sciences, Goethe-University Frankfurt, Frankfurt, Germany; 4 Faculty of Sports- and Rehabilitation Sciences, Laboratory of Biometry, Vrije Universiteit Brussel, Belgium; 5 Department of Health Professions, Bern University of Applied Sciences, Bern, Switzerland; Campus Bio-Medico University of Roma, ITALY

## Abstract

**Background:**

WATSU (portmanteau word: water and shiatsu) is a form of passive hydrotherapy in chest-deep thermoneutral water (35°C = 95°F = 308.15 K). It combines elements of myofascial stretching, joint mobilization, massage, and shiatsu and is reported to be used to address physical and mental issues. The objective of this systematic review (PROSPERO Registration No. CRD42016029347) and the meta-analyses was to assess the applications, indications, and the effects of WATSU to form a basis for further studies.

**Methods:**

A search for “WATSU OR watershiatsu OR (water AND shiatsu)” was conducted without any restrictions in 32 databases. Peer reviewed original articles addressing WATSU as a stand-alone hydrotherapy were assessed for risk of bias. Quantitative data of effects on pain, physical function, and mental issues were processed in random model meta-analyses with subgroup analyses by study design. Effect sizes were expressed as Hedges's g (± 95% confidence intervals).

**Results:**

Of 1,906 unique citations, 27 articles regardless of study design were assessed for risk of bias. WATSU has been applied to individuals of all ages. Indications covered acute (e.g. pregnancy related low back pain) and chronic conditions (e.g. cerebral palsy) with beneficial effects of WATSU regarding e.g. relaxation or sleep quality. Meta-analyses suggest beneficial effect sizes of WATSU on pain (overall Hedges’s g = -0.71, 95% CI = -0.91 to -0.51), physical function (overall Hedges’s g = -0.76, 95% CI = -1.08 to -0.44), and mental issues (overall Hedges’s g = -0.68, 95% CI = -1.02 to -0.35).

**Conclusion:**

Various applications, indications and beneficial effects of WATSU were identified. The grade of this evidence is estimated to be low to moderate at the best. To strengthen the findings of this study, high-quality RCTs are needed.

## Introduction

WATSU (portmanteau word: English "water" and Japanese 指圧 "Shiatsu”) was first described by its originator Dull in the 1980s as a treatment consisting of Japanese Shiatsu bodywork applied in thermal water [1]. To practice WATSU, a therapist stands in thermoneutral water (35°C = 95°F = 308.15 K), supporting the supine receiver with hands, forearms, or shoulders and softly moving her / him in slow and spacious circular motion sequences following elaborate movement patterns related to receiver’s and therapist’s level of experience [1]. The hands of the therapist function as a grip to facilitate movement and at the same time to stimulate acupuncture points. Gentle traction is applied to the body of the receiver to mobilize joints and stretch myofascial structures, as well as *meridians*, channels through which the life-energy (Chinese 氣 “qi”, flows in the concept of Traditional Chinese Medicine [1, 2]. During immersion, hydrostatic pressure influences fluid distribution, metabolism, and respiration. The impact of gravity is greatly reduced, thus decreasing joint loads and allowing maximal flexibility in the positioning of the treated individual [3, 4]. The thermoneutral temperature of 35°C is recommended because it allows passive immersion of about 60 minutes without causing temperature-induced stress [1, 5–7].

Originally, WATSU was created as a non-therapeutic application to support wellbeing and relaxation, and was consequently adopted by therapists. Therefore, therapeutic indications of WATSU are reported in the literature, e.g. to address musculoskeletal conditions [8, 9] neurologic diseases [10–13], and mental distress [14–16], to complement palliative care [17, 18], or to meet the needs of cognitively impaired individuals [19–22]. Originating in the Asian philosophy of maintenance and restoration of health, WATSU can be considered as a floating massage, a tool for rehabilitation, a guided meditation to foster mindfulness and resilience, and a mediator of personal and spiritual growth [8, 14, 23–25].

However, the literature concerning WATSU appears to have been neither systematically reviewed nor evaluated to date. In other words, the evidence to support its use as a differentiated treatment with specific objectives is not refined yet.

Therefore, the aim of this systematic review was to assess the applications, indications, and the effects of WATSU to form a basis for further studies. A comprehensive overview of the current scientific knowledge on the non-therapeutic and therapeutic use of WATSU is provided, covering populations where WATSU is applied, as well as indications and reported effects. In addition, meta-analyses concerning the effectiveness of WATSU as reported in the retrieved literature are presented.

## Methods

This systematic review was registered in advance at PROSPERO (Registration No. CRD42016029347). It was conducted following the Cochrane Handbook for Systematic Reviews [26] and reported according to the Preferred Reporting Items for Systematic Reviews and Meta-Analyses (PRISMA) Guidelines [27, 28].

### Eligibility criteria, search strategy, and screening

Since a preliminary PubMed-search with the term (trademark) “WATSU” resulted in only five articles, a highly sensitive systematic electronic literature search without any filters, restrictions or extensive use of Boolean operators (regarding applications, indications, effects etc.) was conducted in 32 databases: CINAHL, Cochrane Central Register of Controlled Trials (CENTRAL), EBSCO (Dentistry & Oral Sciences Source, MEDLINE, SocINDEX, SPORTDiscus), EU Clinical Trials Register, German Clinical Trials Register, Google Scholar, LIVIVO (MEDPILOT), Ovid (Embase, Books@ovid, Journals@ovid, LWW, MEDLINE, PsycARTICLES), Physiotherapy Evidence Database (PEDro), ProQuest (ProQuest Dissertations & Theses Global, American Periodicals, British Periodicals, Periodicals Archive Online, Periodicals Index Online), PubMed, ResearchGate, ScienceDirect, Swissbib, The Cochrane Library, U.S. National Institutes of Health’s Clinical Trials Register, the homepage of WABA (Worldwide Aquatic Bodywork Association), Web of Science (Web of Science TM Core Collection, Korean Journal Database, SciELO Citation Index), and World Health Organization’s International Clinical Trials Registry Platform (ICTRP). The sensitive search strategy “WATSU OR watershiatsu OR (water AND shiatsu)” was adapted to meet the specific requirements of each database. It was kept up-to-date by alerts or rechecked prior to final analysis (October 3^rd^, 2019). Additional records were retrieved via reference lists of retrieved publications, authors of publications on WATSU, and Institutes of Aquatic Bodywork (of the United States of America, Germany, Austria, and Switzerland).

Procedures of data screening were predefined employing the PICOS approach [27] (*P*opulation: humans (not animals), *I*ntervention: passive hydrotherapy WATSU as stand-alone treatment (not only warm-up or cool-down in aquatic exercise program, no cognate passive hydrotherapies), *C*omparison: not applicable (n/a): all designs, *O*utcomes: applications (treated individuals or groups, e.g. children), indications (treated conditions, e.g. low back pain), effects (pre- post-intervention changes, e.g. decrease of pain), *S*tudy design: original peer reviewed articles (any design, including conference papers and posters)) and performed by two independent reviewers each (AMS and (AH or AM or AS, or PWS)). Exclusion and inclusion were in a first cycle based on titles and abstracts, followed by assessments of full text content. Consensus on disagreements regarding eligibility was reached through discussion. Studies concerning effects of WATSU as a stand-alone hydrotherapy, which were reported in peer reviewed original articles (regardless of study design) were submitted to assessment for risk of bias. If articles were reporting quantitative data, these were considered for meta-analysis. Studies that met the above criteria but described only adaptations in the administration of, or additions to WATSU were excluded [15, 16, 18, 20, 29, 30]. Case reports (CRs) were assessed for risk of bias but not considered in the meta-analysis [11, 31–35]. Primary (original studies) and secondary (reviews, expert opinions) peer reviewed and grey literature (books, theses, conference proceedings) that did not fulfill inclusion criteria for risk of bias assessment but was identified as fitting the scope of the review was described narratively.

### Data extraction, quality assessment, and risk of bias

Data was extracted by two independent reviewers (AMS and CB) using a customized extraction sheet capturing study identification, location, language, search engine(s) / database(s), study design, timeframe of the intervention series, number of sessions delivered in this timeframe, total participants (N) / participants receiving WATSU (n_W_), application(s) / participant descriptors, indication(s) of WATSU reported on, water temperature in°C, duration of sessions in minutes, reports on negative side effects / adverse events, tools used to assess effects, and described effects. If data were found to be missing or unclear, the authors of the study were contacted for clarification or additional data. Since the search was performed without any restrictions, it was necessary to execute translations from the original language to English. If translations via *Google Translate* did not produce sufficiently comprehensible results or appeared to be misleading, colleagues with advanced skills in the concerned languages were contacted. Any disagreements in data extraction and quality assessment were solved by discussion.

Risk of bias was evaluated by two independent reviewers (AMS and CB) pursuant to the Cochrane Handbook for Systematic Reviews [36], employing its review management tool RevMan [37]. Items suggested by the Agency for Healthcare Research and Quality [38], and the Norwegian Knowledge Centre for the Health Services [39] were additionally incorporated into the questionnaire for a total of 19 criteria to address the diversity of study designs. Each item was scored “+” if the criterion was estimated indicating low risk of bias, “n/a” if the criterion was not applicable due to study design, “?” if information necessary to determine risk of bias was missing, and “-”if the criterion was determined to indicate high risk of bias.

### Data synthesis and analysis

Random-effects model meta-analyses of the reported quantitative data of WATSU were conducted. The conduction of meta-analyses was pre-specified in the PROSPERO-protocol for variables for which enough comparable data could be retrieved. The different individual study effect sizes were standardized by dividing the effect sizes by the pooled standard deviations. To correct for potential overestimation of the true effect size in small study samples, effect sizes were expressed as Hedges’s g [40]. Individual study effect sizes and their corresponding 95% confidence intervals (95%CI) as well as the overall weighted estimate and its 95% confidence interval were presented in a forest plot. If more than one randomized controlled trial (RCT, plural: RCTs) was included, subgroup analyses by RCTs and non-RCTs were performed, as pre-specified in the protocol. The difference in the direction of the scales (e.g. Visual Analog Scale (VAS) versus SF-36) was adjusted. If groups in controlled trials exhibited differences in baseline outcome measurements that were suspected to introduce risk of bias (e.g. incomplete wash-out in cross design), only data of the WATSU-intervention group were considered for meta analyses of the concerned variables. When several points in time were reported (e.g. baseline, pre- and post-WATSU, follow-up), those closest to the intervention were analyzed. When two or more scales were employed to describe a domain (e.g. VAS and SF-36 for pain) in a study, the one more frequently used in the included studies was analyzed. If several values were fully reported (e.g. range of motion in several joints), the values were pooled for further processing. When several calculated values (e.g. means and standard deviations of pain in different tasks) were reported, the number of participants was split to process all available data while avoiding artificially increased precision. If values were not reported in numbers, they were estimated from charts.

Heterogeneity was assessed by Cochran’s Q-test, its degrees of freedom and corresponding *p*-value. The degree of heterogeneity was represented as Higgins’ *I*^*2*^ which was calculated as a relative measure (i.e. a proportion) of between-study variability [41]. Higgins’ benchmarking was used for the interpretation of the *I*^*2*^ value. According to this, an *I*^*2*^ between 0% and 40% indicates that the heterogeneity might not be important while an *I*^*2*^ around 30% to 60% might present moderate, from 50% to 90% substantial, and from 75% to 100% considerable heterogeneity [26]. The Comprehensive Meta-Analysis 2 software (CMA–Version 2 Professional, Biostat Inc., Englewood, USA) was used for calculating the effect sizes and the pooled estimate, as well as for establishing the forest plots.

Since only eleven, ten and five studies were included in the different meta-analyses for pain, physical function and mental effects, respectively, publication bias was not statistically analyzed [36]. Level (according to the Oxford Centre for Evidence-Based Medicine, CEBM) and grade (according to the Grading of Recommendations Assessment, Development and Evaluation working group, GRADE) of evidence were reported [42, 43].

## Results

### Study selection

**Fig 1:**
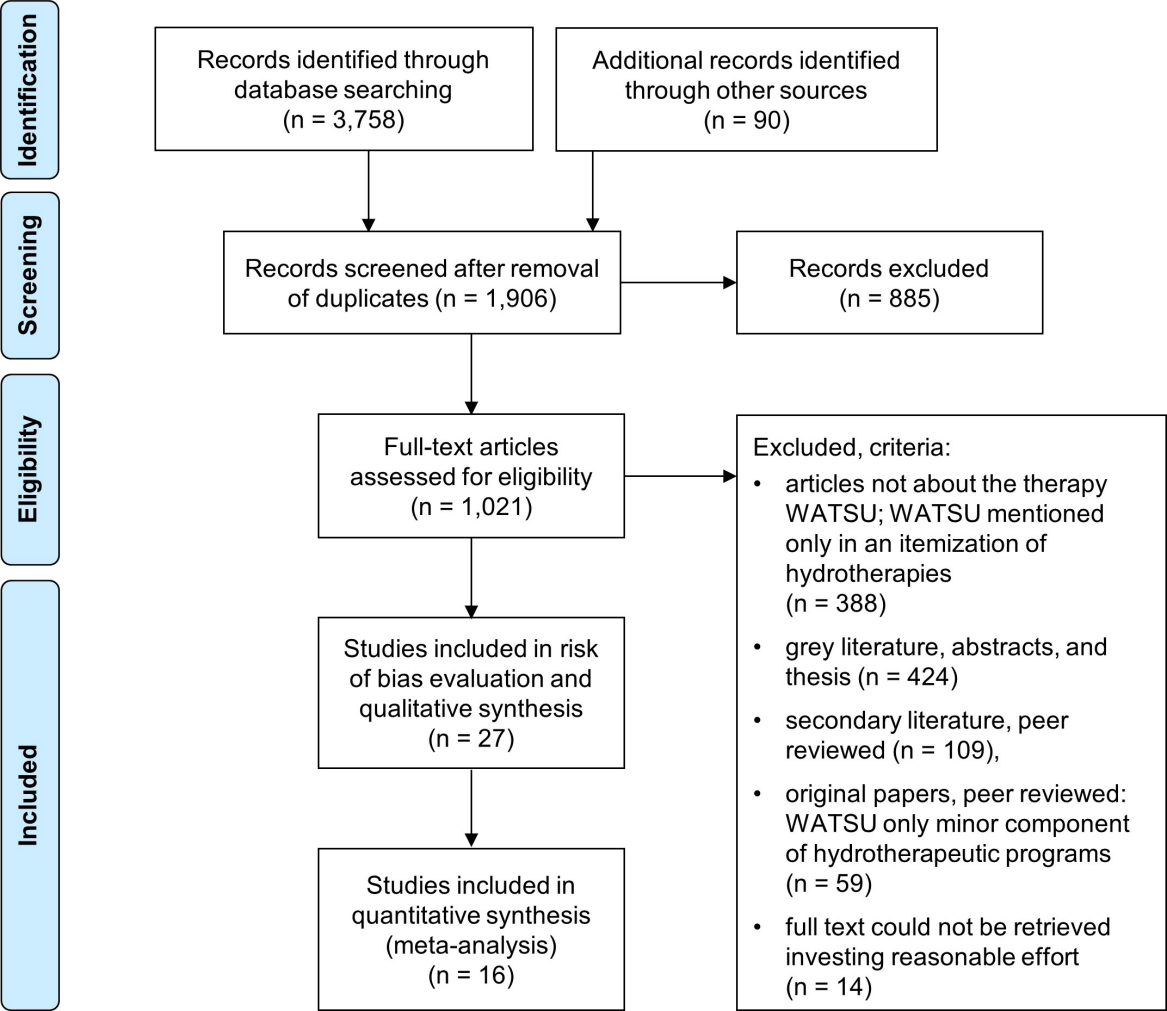
Flow chart depicting the selection progress [28].

The search strategy resulted firstly in a total of 3,848 (3,758 + 90) citations. Of the 195 peer reviewed articles (109 excluded secondary articles + 59 excluded original articles with WATSU as minor component of hydrotherapeutic programs + 27 included articles), 27 studies met the eligibility criteria and were further assessed in the present review (Fig 1, Table 1).

**Table 1:** Characteristics of the articles assessed for risk of bias.

Study ID	Origin / Language	Search engine / database	Design	Time-frame in days	How many WATSU sessions	N	n_W_	Application / participant descriptors	Indication	Water temperature in°C	Duration of sessions in minutes	Negative side effects observed	Assessment tools or outcome variables	Results / reported effects of WATSU
**Antunes et al., 2016**	**Brazil / por**	**Initially in references, GS, CINAHL, Swissbib**	**CS**	**35**	**10**	**17**	**17**	**f, 67.5 ± 4.7 yrs**	**Fibromyalgia**	**39**	**49**	**?**	**VAS allergy, SF-36**	**Sig. improvements in all scales except general health and social function**
Barbosa et al., 2014	Brazil / eng	GS, EBSCO, Swissbib, Web of Science, Researchgate	CR	70	10	1	1	f, 62 yrs; no general body pain or diagnosed fibromyalgia, onset ≥1 yr	Temporo-mandibular disorder	35	40–60	?	STAI, GHQ, WHOQOL-BREF	Sig. improvements in all scales
**Borges & Parizotto, 2001**	**Brazil / por**	**GS, Swissbib, Researchgate**	**CS**	**?**	**5**	**18**	**18**	**17 f, 1 m, 20–60 yrs; pain intensity 4-10/10 (average 6.9)**	**Stress, tension of postural musculature**	**34–36**	**50**	**?**	**VAS pain, respiratory rate, BP, HR**	**Sig. less pain, increased signs of relaxation**
**Campos et al., 2018**	**Brazil / por**	**GS**	**CS**	**42**	**6**	**11**	**11**	**11 f, 18 to 65 yrs**	**Stress, sleep disorder and pain**	**34**	**45**	**no**	**BP, HR, Job Stress Scale, VAS pain, finger-floor**	**Sig. improvements in all scales**
**Chen et al., 2018**	**Israel / heb**	**EBSCO, CINAHL**	**CT**	**58**	**8**	**20**	**10**	**f, 40–75 yrs; pain for ≥ 2 yrs**	**Fibromyalgia**	**34–35**	**30**	**?**	**PSQI, VAS pain**	**Sig. improvements in all scales**
**Chon et al., 2009**	**Korea / eng**	**GS, Swissbib, CINAHL, Embase, Web of Science, PubMed, EBSCO, Livivo, SPORTDiscus Researchgate**	**CS**	**56**	**40**	**3**	**3**	**2 f, 1 m, 49–62 yrs; stroke 8–20 months ago, all independent walkers**	**Hemiplegia**	**34**	**40**	**?**	**TAS, RVGA**	**Descriptive statistics only**
**Chun et al., 2006**	**Korea / kor**	**GS**	**RCT**_**c**_	**?**	**12**	**3**	**3**	**1 f, 2 m, 55–64 yrs; stroke 23–31 months ago**	**Hemiplegia**	**34**	**40**	**?**	**TAS**	**Sig. improvement**
**Cunha et al., 2010**	**Brazil / por**	**Initially in references, then GS**	**CS**	**1**	**1**	**30**	**30**	**f, 20–52 yrs (mean 27.7); healthy**	**Experiment**	**35**	**40**	**?**	**BP, flexibility, HR, qualitative data**	**Sig. more flexibility, increased sings of relaxation**
Dornelas, 2011	Brazil / por	GS, Embase, Researchgate	CR	35	15	1	1	f, 23 yrs; paraplegic	Jarcho-Levin- Syndrome	34	40	?	Goniometer, AIS, FIM, Modified Ashworth Scale	Sig. improvements in all scales
**Faull, 2005**	**New Zealand / eng**	**EBSCO, Swissbib, Science direct, PEDro, Embase, Researchgate, SPORTDiscus, GS, CINAHL**	**RCT**_**c**_	**14**	**4**	**13**	**13**	**f, 26–65 yrs (mean 46.3); mean onset 4.3 yrs ago**	**Fibromyalgia**	**32–35**	**45**	**?**	**SF-36**	**Sig. improvements in physical function, bodily pain, vitality, social function**
**Gimenes et al., 2006**	**Brazil / por**	**Embase, Researchgate, GS, Web of Science, Swissbib, Livivo**	**CS**	**112**	**?**	**10**	**10**	**10 f, 40–82 yrs (mean 53.37)**	**Fibromyalgia**	**?**	**?**	**?**	**VAS pain, GDS**	**Sig. improvements in all scales**
**Gimenez & Castro 2018**	**Brazil / por**	**GS**	**CS**	**8**	**7**	**3**	**3**	**2 f, 13/14 yrs; 1m, 15yrs**	**Cerebral palsy**	**33–35**	**30**	**?**	**PSQI, Modified Ashworth Scale**	**Sig. improvements in all scales**
Hora et al., 2017	Brazil / por	GS	CS	1	1	36	36	53% f, 47% m, 24.6 ± 6 yrs; healthy students	Experiment	34.25 ± 2.82	40–45	no	BP, HR, VAS pain	Sig. lower BP and HR during immersion; VAS not reported
**Israel et al., 2006**	**Brazil / eng**	**Private archive**	**CS**	**?**	**15**	**3**	**3**	**3 m, 25–60 yrs**	**Ankylosing spondylitis**	**34**	**30**	**?**	**NRS pain**	**Descriptive statistics only**
Jithin & Adarsh, 2019	India / eng	GS	RCT	49	21	20	10	10–14 yrs; young swimmers	Experiment	35	?	?	HR, BP, body temperature	Sig. increased signs of relaxation, lower body temperature
Lima et al., 2009	Brazil / por	GS, Embase, Researchgate	CR	35	10	1	1	f, 21 yrs; moderate asthma	Asthma	34	60	?	FVC, FEV1, FEV1 / FVC, PEF, HR, RR, SpO_2_, STAI, BDI, QQV-Asma, chest circometry	Sig. improvements in all parameters except FVC, SpO_2_ and HR
Mota et al., 2007	Brazil / por	GS	CS	1	2	28	28	f, m, 22.82 ± 4.65 yrs; healthy students	Experiment	33–34	40	?	HRV	Sig. increased signs of relaxation / adaptation
Nakamoto, 2016	Brazil / por	Initially private archive, then GS	CR	35	10	1	1	f, 63 yrs; history of falls	Fall prevention	33–35	60	?	Berg-Balance-Scale	Sig. improvement
Pastrello et al., 2009	Brazil / por	GS, CINAHL, EBSCO, Researchgate	CR	112	16	1	1	m, 4 yrs; spastic quadriplegic cerebral palsy, GMFM level V	Cerebral palsy	33	30	?	GMFM	Sig. improvement
**Pinkalsky et al., 2011**	**Brazil / por**	**GS**	**CS**	**35**	**5**	**5**	**5**	**f on waiting-list for physiotherapy, 50–68 yrs**	**Fibromyalgia**	**34**	**60**	**?**	**VAS pain, WHOQOL-BREF, FIQ**	**Sig. improvements in all scales except mental health, environment, and FIQ**
**Rambo & Filippin, 2019**	**Brazil / por**	**GS**	**CS**	**1**	**1**	**15**	**15**	**Newborns at neonatal Intensive Care Unit**	**Pain in premature newborns**	**37**	**10**	**no**	**HR, RR, MAP, SpO**_**2**_**, PIPP, Brazelton**	**No significant changes**
**Ramirez et al., 2019**	**Brazil / por**	**GS, PubMed, Livivo, Web of Science, Researchgate MEDLINE, Embase**	**RCT**	**70**	**10**	**30**	**16**	**76.1% f, 8–18 yrs**	**Juvenile idiopathic arthritis**	**?**	**45**	**no**	**PedsQL 4.0, CHAQ, GROMS**	**Sig. improvements in all scales except PedsQL 4.0 QoL scale**
**Ribeiro et al., 2019**	**Brazil / eng**	**GS, Researchgate**	**CS**	**35**	**9**	**4**	**4**	**m, 60.5 yrs**	**Parkinson**	**35**	**60**	**?**	**BAI, BDI, Katz Index, Tinneti’s Scale, PDQ-39**	**No significant changes**
Schitter & Fleckenstein, 2018	Switzerland / eng	GS, PubMed, EBSCO, Web of Science, MEDLINE, Swissbib, Researchgate, Livivo	CR	56	8	1	1	f accident survivor, 52 yrs; 6 weeks after accident	Rehabilitation multiple trauma (incl. pelvic ring)	35	60	Adverse finding: swelling of knee after first session	NRS (general condition, emotional, mental, physical), pain medication, circumference knee, PSFS, qual. data	Sig. improvements in all scales
**Schitter et al., 2015**	**Switzer-land / eng**	**GS, EBSCO, Cochrane, Embase, Web of Science, Livivo, PubMed, Science direct, Swissbib, CINAHL Researchgate**	**CT**	**8**	**2**	**17**	**9**	**f, mean 32 ± 3.2 yrs; healthy, >34th week of pregnancy**	**Pregnancy-related com-plaints, breech presentation**	**35**	**60**	**no**	**PSS, SF-36, VAS (pain, stress), MDMQ, USE, qualitative data**	**Sig. improvements in all scales except SF-36 physical component**
Tufekcioglu et al., 2018	Turkey / eng	GS, Researchgate	RCT	84	24	34	13	f, m, 8.30 ± 0.31 yrs; obese with BMI above 30	Obesity	32	30	?	HRV	No significant effects between the three conditions
Wieser, 2007	USA / eng	GS, SPORTDiscus, EBSCO	CS	?	?	19	19	f, m, 6–21 yrs; 9 autistic, 10 with severe handicap	Autism, multiple handicaps	?	30	?	Narrative report	No quantitative data reported

Studies that were included in the meta-analyses are in **bold** print.

Abbreviations: AIS: American Spinal Injury Association Impairment Scale, BAI: Beck's Anxiety Inventory, BDI: Beck's Depression Inventory, BP: Blood Pressure, Brazelton: Brazelton Adapted Sleep-Wake Rating Scale, CHAQ: Childhood Health Assessment Questionnaire, CHQ-PF50: The Child Health Questionnaire Parent's Form, CR: Case Report, CS: Case Series, CT: non-randomized Controlled Trial, eng: English, esp: Spanish, f: female(s), FEV1: Forced Expiratory Pressure in 1 Second, FIM: Functional Independence Measure, FIQ: Fibromyalgia Impact Questionnaire, FKB: Fragebogen zum Körperbild [Body Perception Questionnaire], FKKS: Frankfurter Körperkonzeptskalen [Frankfurt Body Concept Scales], FVC: Forced Vital Capacity, GDS: Geriatric Depression Scale, Genuss-Fragebogen [Pleasure-Questionnaire], ger: German, GHQ: General Health Questionnaire, GMFM: Gross Motor Function Measure, GROMS: 10-joint Global Range of Mobility Scale, GS: Google Scholar, heb: Hebrew, HR: Heart Rate, HRV: Heart Rate Variability, ICD-10: International Statistical Classification of Diseases and Related Health Problems 10th Revision, m: male(s), kor: Korean, MAAS: Mindful Attention Awareness Scale, MAP: Mean Arterial Pressure, MDMQ: Multidimensional Mood State Questionnaire, N: Participants total, n_W_: Participants receiving WATSU, NRS: Numeric Rating Scale, PDQ-39: Parkinson’s Disease Questionnaire-39, PedsQL 4.0: Pediatric Quality of Life Inventory Scale, PEF: Peak Expiratory Flow, por: Portuguese, PIPP: Premature Infant Pain Profile, PSFS: Patient Specific Functional Scale, PSQI: Pittsburgh Sleep Quality Index, PTSD: Post Traumatic Stress Disorder, QoL: Quality of life, QQV-Asma: Questionário de Qualidade de Vida na Asma [Quality of Life Questionnaire of Asthmatic Patients], RCT: Random Controlled Trial, RCT_c_: RCT with cross design, RR: Respiratory Rate, RVGA: Rivermead Visual Gait Assessment, SF-36: 36-Item Short Form Health Survey, Sig.: statistically significant(ly), SpO_2_: Peripheral Oxygen Saturation, SOC: Sense of Coherence, Spielerisches Welterleben [Playful Worldview], STAI: State-Trait-Anxiety Inventory, TAS: Tone Assessment Scale, TWSTRS: Toronto Western Spasmodic Torticollis Rating Scale, USE: Ultrasound Examination, VAS: Visual Analog Scale, WHOQOL-BREF: World Health Organization Quality of Life Measurement (short version), yr(s): year(s) (of age).

### Study designs and assessments

Among the fully identified articles of the search of this systematic review, WATSU appeared from 1990 in the grey literature and from 1994 in the secondary scientific literature [44–48]. Efforts to scientifically assess WATSU were observed since 1998 [49]. Studies fully meeting the inclusion criteria have been carried out since 2001. Research on WATSU was conducted worldwide and in several languages: Of the 27 articles assessed for risk of bias, 14 were written in Portuguese, 11 in English, one in Hebrew, and one in Korean.

The five RCTs in the assessment were from Korea [10], New Zealand [8], India [50], Brazil [51], and Turkey [52], and the two non-randomized controlled trials (CTs) were from Israel [53] and Switzerland [9]. Control groups underwent no treatment [9, 52], massage [8], active hydrotherapy [51, 53], passive stretching on land and in water [10] or were not specified [50].

The remaining 20 studies reported on repeated measure designs involving within-group comparisons (14 case series, CSs [13, 54–65] including a collection of narrative retrospective reports [21]), and on single cases (6 CRs) [11, 31–35]. Tools chosen for assessment of the effects of WATSU considered physical and mental aspects. Water temperatures, duration of sessions, periods of administration of WATSU, and treatment-intervals varied between the different studies (32–39°C (34.5 ± 1.3), 10 minutes (for premature newborns), in general from 30 to 60 minutes (43.9 ± 11.3), one day to 16 weeks, daily to weekly, respectively).

The peer reviewed secondary literature comprised 109 articles, and the grey literature included a total of 424 items: 38 abstracts / conference proceedings, 156 theses, 99 books / book chapters, and 131 non-scientific contexts such as magazine-articles or webpages).

### Applications, indications, and general effects

Detailed applications, indications and effects of the articles that were assessed for risk of bias are depicted in Table 1. In these studies, WATSU was applied in childhood, adulthood, and advanced age. The indications included musculoskeletal disorders (e.g. fibromyalgia, rehabilitation), and neurological (e.g. cerebral palsy, hemiplegia) and mental challenges (e.g. stress, sleep disorder). In addition, four experiments with healthy participants were conducted to study physiological effects of WATSU (e.g. changes of heart rate or blood pressure).

Applications and indications of WATSU as reported in the primary peer reviewed literature were contentually comparable with the secondary peer reviewed and grey literature. In both, WATSU was mainly reported to be used for the treatment of chronic conditions (e.g. fibromyalgia, asthma, neurologic conditions, geriatric care), and it was also described as one component of palliative care (e.g. during cancer and vigil coma state) [17, 18, 66–76]. Most mentions referred to implementation of elements of WATSU in hydrotherapeutic interventions / aquatic exercises.

Concerning WATSU as a stand-alone therapy, the grey literature frequently focused on qualitative aspects of the experience of WATSU and its potential to address psychological trauma [14, 77]. A central theme was being held and carried by the therapist throughout the treatment. The therapist’s facilitation of the receiver’s comfort, and provision of unconditional support are being considered pivotal to the therapeutic effects of WATSU [1, 12, 23, 78, 79].

The exceptional sensorimotor experiences reported during WATSU included the perception of weightlessness, sometimes interpreted as the notion of omnipresent support, or being back in the mother’s womb [80]. Moreover, WATSU was credited with facilitating emotional growth, spiritual experiences, enhanced states of awareness, altered states of consciousness, visions of vivid colors with eyes closed, the impression of flying or floating in the air, feelings of utter connectedness with all beings, and inner stillness [1, 14, 23, 24, 77, 80–83].

### Meta-analyses

#### Effect of WATSU on pain

**Fig 2:**
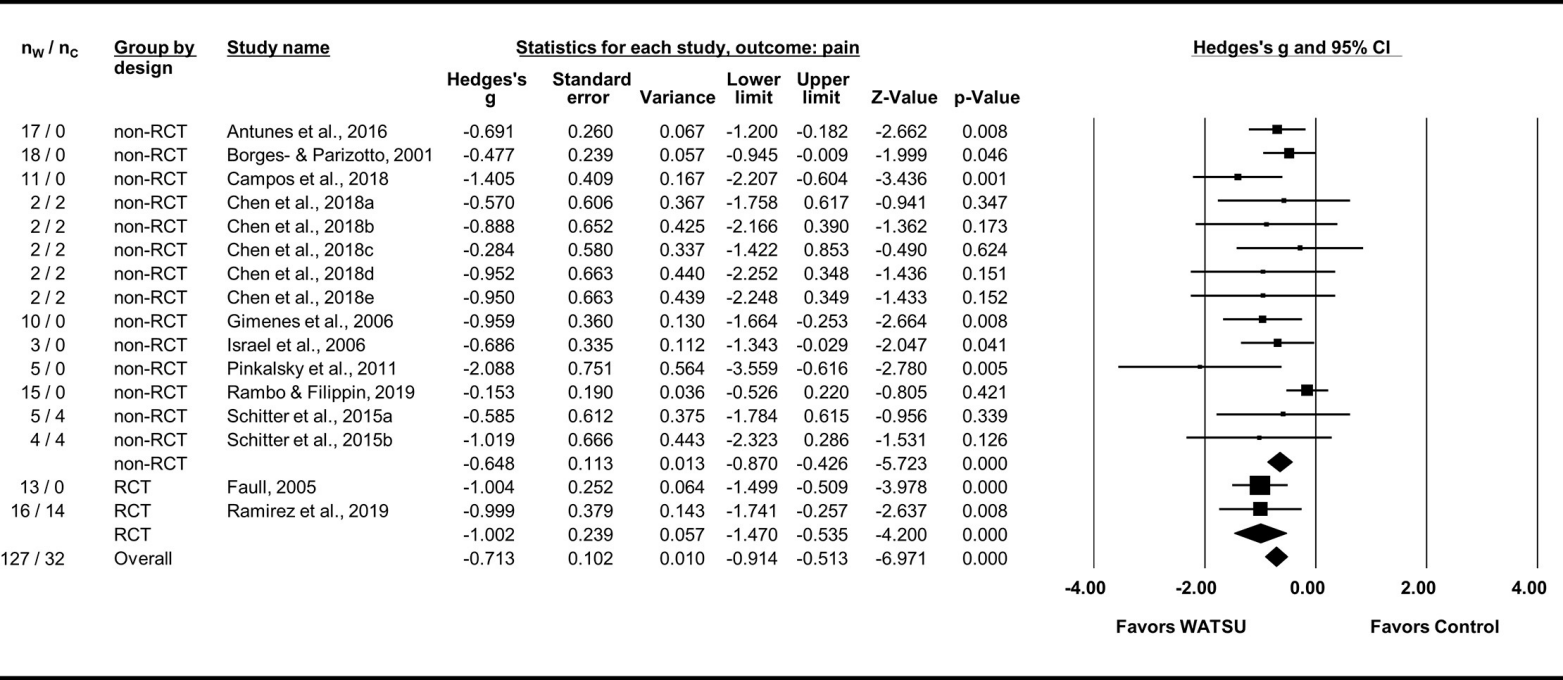
Forest plot of random model meta-analysis for effects of WATSU on pain. Subgroup analyses by design (non-RCTs versus RCTs) table depicting number of participants in intervention group (receiving WATSU, n_W_), number of participants in control group (n_C_), total number of participants in these groups, Hedges’s g, standard error, variance, CI, Z-value, and p-value. The number of participants in the 2015 study by Schitter et al. was split and used twice (as Schitter et al., 2015a: day 1 of the study, 1^st^ treatment, and 2015b: day 4, 2^nd^ treatment) to process all available data while avoiding artificially increased precision. Likewise, the number of participants in the 2018 study by Chen et al. was split and used five times for the various positions reported (as Chen et al., 2018a: sitting, 2018b: walking, 2018c: driving, 2018d: standing, and 2018e: lying). Detailed pain scores from this study were provided by the authors. Data of reported pre- and post-pain-measurements of 15 WATSU sessions in the 2006 study by Israel et al. were estimated from the graph and pooled for further processing. The data of the control group in the 2005 study by Faull was not considered because of baseline-differences in outcome.

The eleven articles included in the meta-analysis for pain (with a total of 159 participants, 127 of them receiving WATSU) reported comparable quantitative data on pain related to fibromyalgia [8, 53, 57, 58, 63], mental and occupational stress [56, 59], juvenile idiopathic arthritis [51], ankylosing spondylitis [65], painful medical interventions in newborns [64], and pregnancy [9]. Pain was assessed by Visual Analog Scale (VAS) [9, 53, 56, 57, 59, 63], Physical Pain Subscale of the 36-Item Short Form Health Survey (SF-36) [8, 58], Numeric Rating Scale [65], Premature Infant Pain Profile (PIPP) [64], and Childhood Health Assessment Questionnaire (CHAQ) [51]. The overall effect size was Hedges’s g = -0.71 (95% CI = -0.91 to -0.51) in favor of WATSU (Fig 2). Overall heterogeneity was *I*^*2*^ = 22% and statistically not significant (Q = 19.212, df(Q) = 15, *P* = 0.204). In the subgroup analysis by study design, RCTs presented with Hedges’s g = -1.00 (95% CI = -1.47 to -0.54), and *I*^*2*^ <1% (Q <0.001, df(Q) = 1, *P* = 0.991). In comparison, the effect observed in non-RCTs was g = -0.65 (95% CI = -0.87 to -0.43), *I*^*2*^ = 20% (Q = 16.211, df(Q) = 13, *P* = 0.238).

The studies reported on short-term effects measured immediately pre- and post-treatment [9, 59, 64, 65], and on the effects measured after two [8], five [58, 63], eight [53], ten [51], and 16 weeks of treatment [57]. Two studies did not report on the applied timeframe [56, 65].

#### Effect of WATSU on physical function

**Fig 3:**
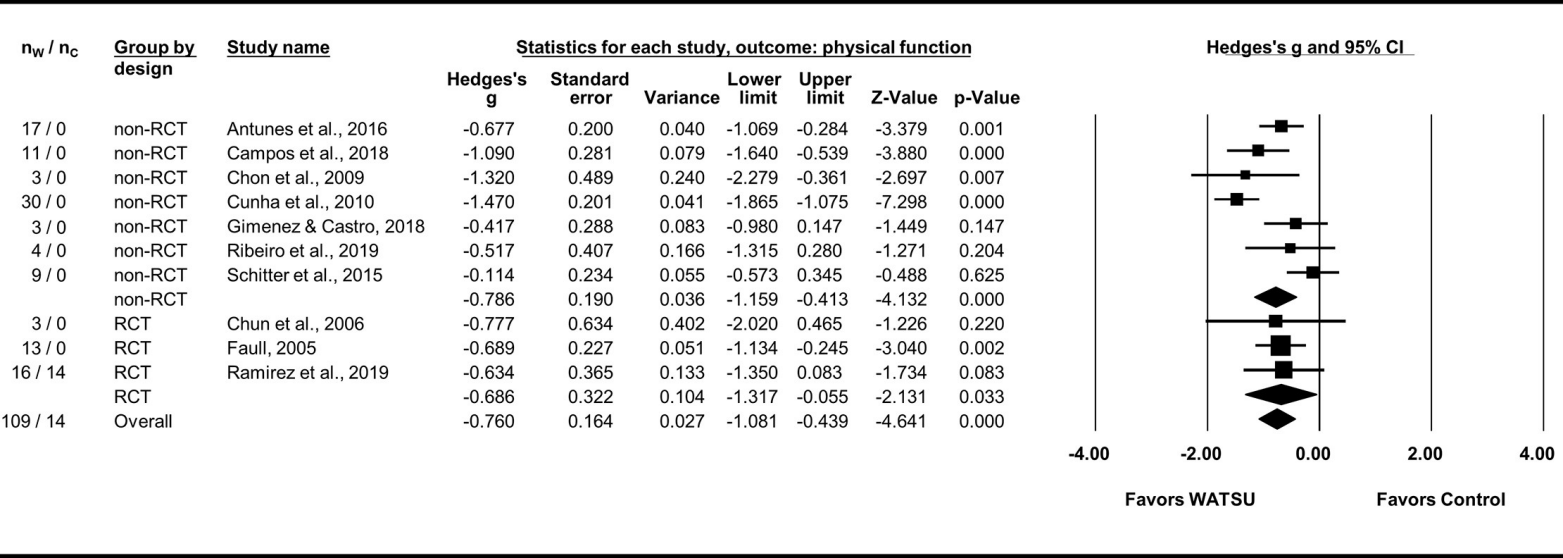
Forest plot of random model meta-analysis for effects of WATSU on physical function. Subgroup analyses by design (non-RCTs versus RCTs), table depicting number of participants in intervention group (receiving WATSU, n_W_), number of participants in control group (n_C_), total number of participants in these groups, Hedges’s g, standard error, variance, CI, Z-value, and p-value. Chun et al., 2006, reported on three participants in a cross design with three interventions. To avoid artificially increased precision, only a comparison between WATSU and the control-intervention with the larger beneficial effect size is presented in the forest plot. Outcome data of several measurements presented in the studies of Chon et al., 2009, Chun et al., 2006, and Gimenez & Castro, 2018, were each pooled for further processing. The data of the control group in the 2005 study by Faull was not considered because of baseline-differences in outcome, data of the control group in the 2015 study by Schitter et al. was not reported for this outcome.

The ten articles included in the meta-analysis on physical function (with a total of 123 participants, 109 of them receiving WATSU) reported comparable quantitative data on gains of physical function related to fibromyalgia [8, 58], occupational stress [59], neurological disorders [10, 13, 60, 61], pregnancy [9], juvenile idiopathic arthritis [51], and healthy individuals [54]. To record effects of WATSU on physical function, range of motion was measured [54, 59] or assessed by the Global Range of Motion Scale (GROMS) [51]. Also, the Tone Assessment Scale (TAS) [10, 13], the modified Ashworth Scale [60], the Physical Function Subscale of the SF-36 [8, 9, 58], and the Katz index [61] were used. The overall effect size was Hedges’s g = -0.76 (95% CI = -1.08 to -0.44) in favor of WATSU (Fig 3). Overall heterogeneity was *I*^*2*^ = 64% and statistically significant (Q = 24.85, df(Q) = 9, *P* = 0.003). In the subgroup analysis, in RCTs an effect of Hedges’s g = -0.69 (95% CI = -1.32 to -0.06) with *I*^*2*^ <1% (Q = 0.041, df(Q) = 2, *P* = 0.980) was observed. In comparison, the effect observed in non-RCTs was Hedges’s g = -0.99 (95% CI = -1.16 to -0.41), *I*^*2*^ = 75% (Q = 24.471, df(Q) = 6, *P* <0.001).

The studies reported on short-term effects measured immediately pre- and post-treatment [54, 59], and on effects measured after one [9], two [8], five [58, 61], eight [13, 60], and ten weeks of treatment [51]. One study did not report on the applied timeframe [10].

#### Mental effects of WATSU

**Fig 4:**
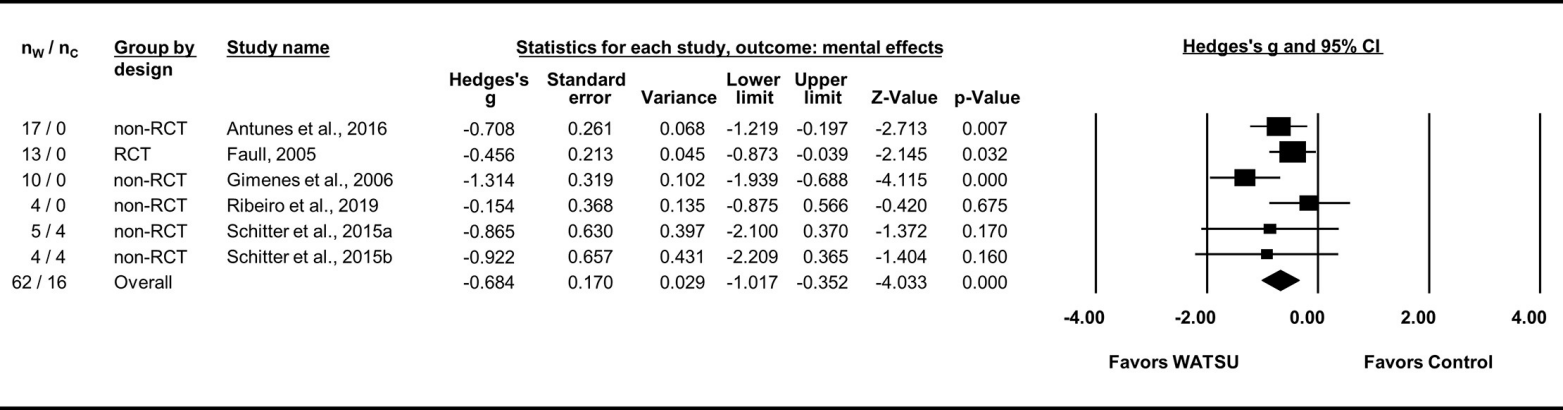
Forest plot of random model meta-analysis for mental effects of WATSU. Table depicting number of participants in intervention group (receiving WATSU, n_W_), number of participants in control group (n_C_), total number of participants in these groups, Hedges’s g, standard error, variance, CI, Z-value, and p-value. The number of participants in the 2015 study by Schitter et al. was split and used twice (as Schitter et al., 2015a: day 1 of the study, 1^st^ treatment, and 2015b: day 4, 2^nd^ treatment) to process all available data while avoiding artificially increased precision. The data of the control group in the 2005 study of Faull was not considered because of baseline-differences in outcome.

The five articles included in the meta-analysis on mental effects (with a total of 78 participants, 62 of them receiving WATSU) reported comparable quantitative data related to fibromyalgia [8, 57, 58], neurological disorders [61], and pregnancy [9]. Mental effects were assessed by the Mental Health Subscale of the SF-36 [8, 58], the Geriatric Depression Inventory (GDI 15) [57], the Beck’s Depression Inventory (BDI) [61], and VAS (stress) [9]. The overall effect size was Hedges’s g = -0.68 (95% CI = -1.02 to -0.35) in favor of WATSU (Fig 4). Heterogeneity was *I*^*2*^ = 31% and statistically not significant (Q = 7.297, df(Q) = 5, *P* = 0.199). Since only one RCT was included (Faull, 2005), a subgroup analysis of this outcome was omitted.

The studies reported on short-term effects measured immediately pre- and post-treatment [9], and effects measured after two [8], five [58, 61], and 16 weeks of treatment [57].

### Risk of bias

**Fig 5:**
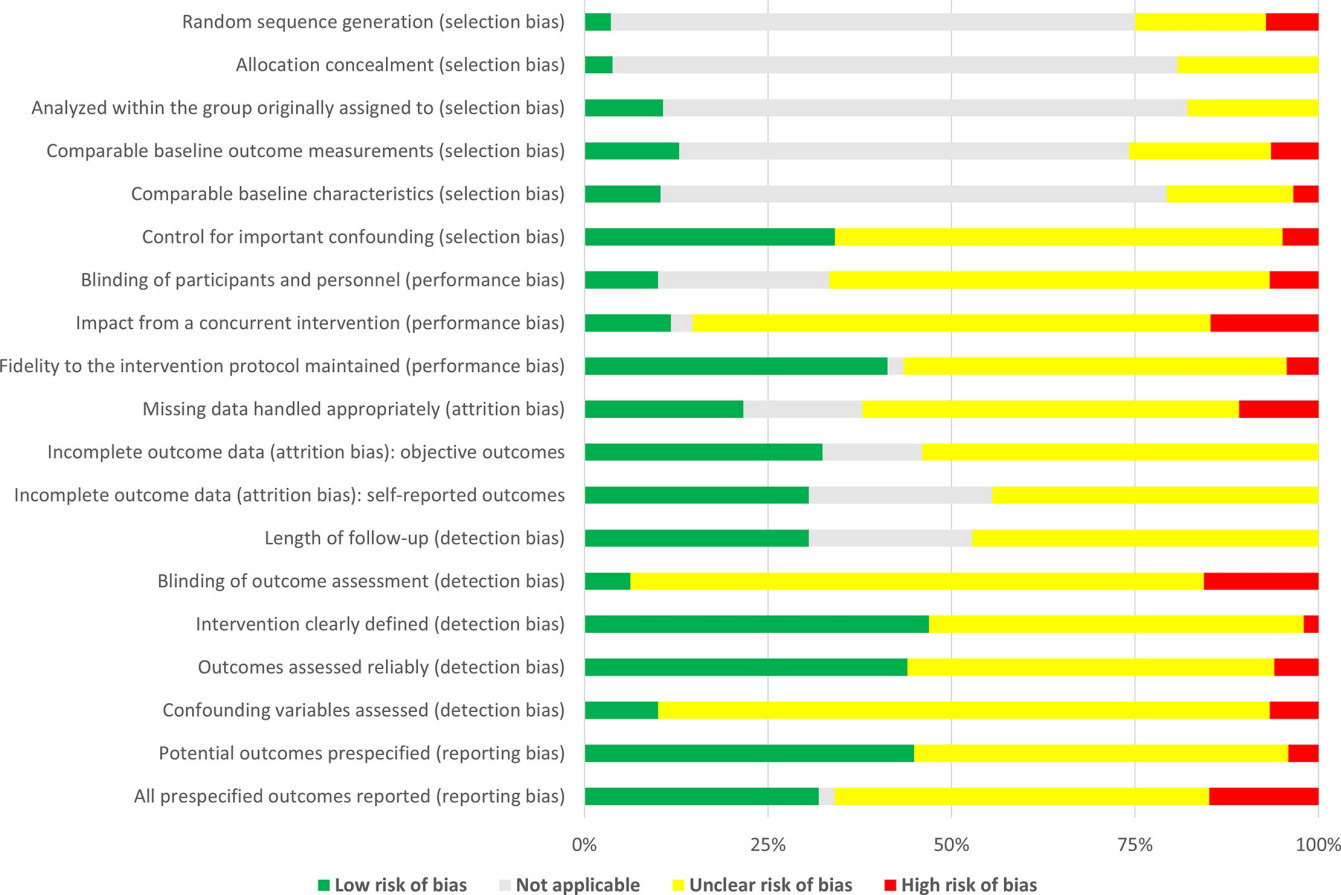
Risk of bias summary. Review authors' judgements about each risk of bias item presented as percentages across all included studies.

**Fig 6:**
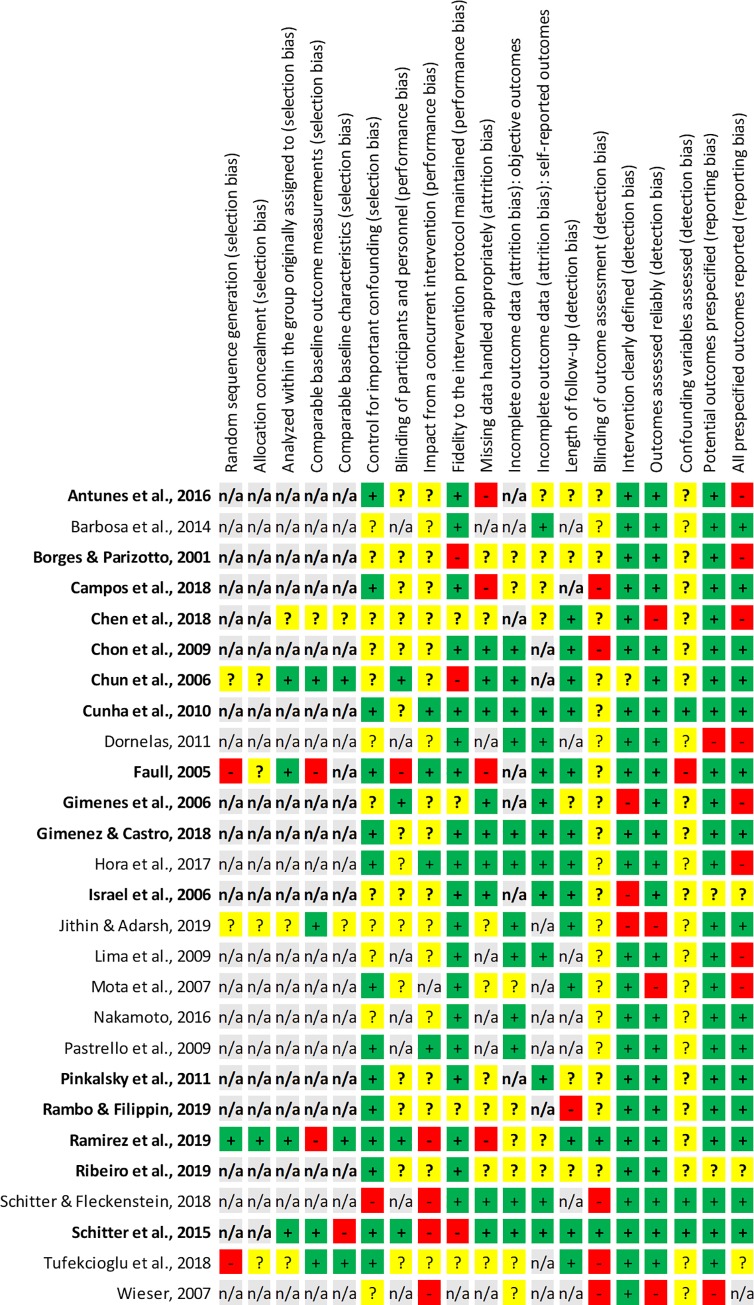
Detailed risk of bias graph. Review authors' judgements about each risk of bias item for each included study. Studies that were included in the meta-analyses are in **bold** print.

Unclear risk of bias was observed in a substantial number of items (see Fig 5 and Fig 6). This was partially inherent in the studies’ design (e.g. CRs), partially due to poor reporting. Two trials with randomized- and one trial with non-randomized controlled design were well documented, they presented overall low risk of bias [8, 9, 51]. When excluding items that were not applicable due to study design, some CSs [13, 54, 60, 62] and CRs [11, 35] were also identified as quite complete in their reporting–all presenting low risk of bias within the scope of their designs.

## Discussion

### Study selection, designs, and assessments

While WATSU has been in use since the 1980s, when it was developed, and mentioned in the primary scientific literature since the turn of the millennium, a systematic review of the literature on its applications, indications, and effects does not yet seem to exist. The studies assessed for risk of bias reflect the overall research on WATSU: carried out in all regions of the world, several languages and a broad spectrum of study designs, they assess both the physical and mental effects of WATSU. Since the search was highly sensitive, only 27 of 3,848 articles were found to meet the PICOS-criteria.

Secondary peer reviewed and grey literature on the topic were also identified and described narratively.

### Applications, indications, and general effects

In the studies included in the risk of bias assessment, WATSU was applied during childhood, adulthood and advanced age. Indications ranged from pain in premature newborns, the needs of cognitively impaired children, pregnancy related complaints, stress, fibromyalgia, hemiplegia to fall prevention. Being held and gently moved by someone in warm water may lead to the perception of a “safe harbor”, eliciting kinesthetic memories of the uterus and early childhood [80]. Social touch is considered a necessity during childhood and remains a resource throughout life [84–87]. Mere gentle physical contact with another human being was shown to attenuate the subjective perception of social exclusion and pain [88, 89]. Touch as therapeutic agent is being discussed in the literature with reference to c-tactile fibers, unmyelinated low-threshold afferents that respond particularly to velocities and temperatures of gentle skin-stroking caress [90, 91]. The idea that warm water could act as a whole-body stimulator for this type of fibers is intriguing, although not scientifically verified [92].

On the other hand, individuals might enjoy–and benefit from–company and solitude to differing degrees based on past experiences of abandonment or inundation [93, 94]. In this regard, certain responder profiles related to attachment style or traumatic life events might determine the success of WATSU and even the indication [95].

During inactive immersion, thermoneutral water seems to fade out of perception after a while–possibly because humans are not provided with hygroreceptors but instead learn to identify water by experience, with cool temperature being one key indicator of wetness [96]. In addition, with ears submerged, familiar noises are missing while unusual noises (e.g. heartbeat) attract the attention. The experience of a persistent lack of solid ground, being held passively and moved for long periods of time—everything that constitutes WATSU is unfamiliar for adults. Such interference with the normal inflow and outflow of stimuli and impulses is perfectly suited to generate altered states of consciousness [97]. One such phenomenon that can easily be quantified is altered perception of time, as reported regarding meditation or the flow phenomenon [98, 99]. When comparing perceived and actual duration of the WATSU sessions in their trials, Cunha et al., 2010, found overestimations (while lasting 36 ± 2 minutes, sessions were perceived to last for one hour or more by 74% of the participants) and Hora et al., 2017, underestimations (sessions lasting 40 ± 5 minutes were perceived as lasting 29.4 ± 1.9 minutes) [54, 62]. Such deviations from normal states of mind, as frequently described in the grey literature about WATSU, have been investigated in depth with respect to sensory isolation in flotation tanks, where individuals float in darkness on the surface of a thermoneutral saline solution [100–102]. Interestingly, despite the human contact in WATSU compared to isolation in flotation tanks, similar effects were reported.

#### Effect of WATSU on pain

The meta-analyses indicated a beneficial effect of WATSU on acute and chronic pain that was observed consistently. Neither important nor statistically significant heterogeneity was evident between the included trials. The effects were measured immediately pre- and post-intervention, but also after up to 16 weeks of treatment. The small effect size observed by Rambo & Filippin, 2019, could be explained by floor-effects [64].

In the subgroup analysis, the beneficial effect was pronounced in RCTs, with very low heterogeneity between the two included RCTs. Two RCTs (and one CT), well documented and with low risk of bias support this evidence, thus qualifying for level 1a according to CEBM. Considering unclear risk of bias in the other eight included studies, the small number of participants, the observed low and non- significant heterogeneity, and the effect size, the overall grade of the established evidence (according to GRADE) was estimated as low to moderate at the best.

Other systematic reviews reporting on moderate to high quality trials comparing aquatic exercise with no therapy indicated beneficial effects for musculoskeletal pain (Cohen’s d = -0.37 [95% CI = -0.56 to -0.18]) and pain in fibromyalgia (Cohen’s d = -0.61 [95% CI = -0.91 to -0.30]) [103, 104]. Pain reduction is a well-known phenomenon in hydrotherapy and subject to explanation models and hypotheses from relaxation after sensory overflow to the potential activation of unmyelinated c-tactile fibers [4, 9, 105]. Pain relieving effects sizes, e.g. in primary dysmenorrhea (moderate grade of evidence, Cohen’s d = -0.43 [95% CI = -0.7 to -0.15]) and labor (very low grade of evidence, raw mean difference in VAS pain (0–100) of 10.30 [95% CI = 4.69 to 15.91]) are also attributed to acupressure, one form of which is Shiatsu [106, 107].

### Effect of WATSU on physical function

The meta-analysis indicated a beneficial effect of WATSU on physical function during chronic conditions and in healthy individuals with substantial and statistically significant heterogeneity among the included trials. Fetal growth between measurements might have contributed to the small effect in the 2015 Schitter et al. study [9].

The subgroup analysis confirmed the effect with very low heterogeneity among the three included RCTs. Six well-documented studies, all with low risk of bias, are included in this analysis (among them two RCTs and one CT), thus overall qualifying for evidence level 1a according to CEBM. Considering unclear risk of bias in the other four included studies (among them another RCT), the small number of included participants, the observed overall heterogeneity, and the effect size, the grade of the established evidence (according to GRADE) was overall estimated as low to moderate at the best.

There is moderate evidence indicating beneficial effects for physical function (Cohen’s d = 0.32 [95% CI = 0.13 to 0.51) in musculoskeletal conditions comparing aquatic exercise with no therapy [103]. In the present meta-analysis, the construct of physical function was connected to lower muscular tone and stiffness, translating to less spasm and increased range of motion. These can be relevant preconditions for successful active exercises. In addition, passive proprioceptive training (as which WATSU could be interpreted) was observed to be surprisingly effective for motor learning, when compared with active exercise and visual demonstration [108].

### Mental effects of WATSU

The meta-analysis indicated beneficial mental effects of WATSU during chronic conditions and in healthy individuals that was observed consistently with neither important nor significant heterogeneity. The beneficial effect was confirmed by the RCT included in this analysis. One RCT and one CT, both well documented and with low risk of bias were included in this analysis, thus overall qualifying for evidence level 1b according to CEBM. Considering the unclear risk of bias in the other three included studies, the small number of included participants, the effect size, and the low heterogeneity, the grade of the established evidence (according to GRADE) was estimated as low to moderate at the best.

While feelings of wellbeing are often central in qualitative descriptions of WATSU, this does not seem to be fully transferable to the effect of WATSU on actual pathologies such as depression (a common parameter in this analysis) [9, 21, 35, 54, 62, 66]. Interestingly, Maczkowiak et al., 2007, reported very large effect sizes of WATSU related to BDI as a result of the combination of reception and in turn administration of WATSU by the study participants, who were clinically depressed [15]. One contributing factor to this result might be increased release of endogenous oxytocin (peptide hormone and neuropeptide which plays a role in social bonding) due to physical contact in combination with received signals of trust [109].

A systematic review on the topic did not report significant effect sizes of aquatic exercise on symptoms of depression in patients with fibromyalgia (Cohens’ d = -0.19 [95% CI = -0.88 to 0.50]) [104].

### Strengths and limitations

Strengths of this systematic review are the sensitive and very comprehensive search in 32 databases without any filters or language restrictions, and the consideration of secondary and grey literature. WATSU is a rather young therapy and embracing languages other than English proved to be very useful considering that only 29 of the 86 retrieved peer reviewed primary articles about WATSU and only 11 of the 27 articles assessed for risk of bias were written in English. The use of *Google Translate* enabled the inclusion of all accessible data. In addition, a pool of native speakers stood by to assist in case of ambiguity.

*Google Scholar* provided all but two of the 27 articles that were assessed for risk of bias. Moreover, 10 of these 27 articles were exclusively found by *Google Scholar* and in no other database. Therefore, *Google Scholar* was a valuable help to complete this search.

The use of *Google Scholar* for systematic reviews has been and is being discussed in the literature [110–113]. While clearly dominating other search engines employed in this review, *Google Scholar* presented other forms of incompleteness: e.g. defining a timeframe or filtering out patents caused the exclusion of interesting articles. Also not all articles listed as search results could actually be retrieved–on the one hand because *Google Scholar* assumed the researchers to actually be robots, on the other hand because only 100 pages (= 1,000 results) could be accessed, no matter the amount of estimated results displayed on top of the screen (e.g. “approximately 1,170 results” ended up as exactly 1,000). Even below this number the last pages with results could not be retrieved (therefore filtered “approximately 880 results” turned to suspicious 800). However, PubMed also reduced its 8 initial results for “WATSU” to 4 for no conceivable reason, once the filter was set on “humans”. Therefore, the question must be raised, whether the term “systematic review”–suggesting thorough retrieval through “exhaustive, comprehensive searching” [114]—can be justified under these conditions at all. This, however, is not a weakness specific to the systematic review reported here but holds true for any systematic literature search.

Due to limited number of included studies, publication bias was not statistically analyzed [36].

On a beginner’s level, motion sequences of WATSU are taught to help students by providing a framework–well educated and experienced practitioners, however, will abandon these sequences and follow the “free flow” where individually-oriented interaction between receiver and therapist is central [23]. Thus, a scientific approach to WATSU is challenging, because standardized procedures are contrary to some of its core principles–a strict execution of predefined motion sequences might be counterproductive for the effect of this form of therapy, in any case it would contradict the underlying idea [1]. Beyond that, the general conditions of the administration of WATSU varied greatly in the retrieved studies: water temperatures varied considerably when WATSU was applied as stand-alone therapy, and in articles that used WATSU to warm up or cool down, water temperatures were even reported to be as low as 25°C (77°F, 298.15 K) [115]. This might be due to pragmatic reasons, e.g. the availability of suitable pools with convenient depth and reasonable water temperature. However, 35°C are clearly defined and recommended as optimal by WATSU’s originator and the Worldwide Aquatic Bodywork Association (WABA) because water at this temperature is thermoneutral [1, 5–7]. Thermoneutrality is defined as the range of temperature that allows one hour of resting without changes in central body temperature. This condition was measured in water to be 35–35.5°C in men in winter in Rochester, New York, at room temperatures between 25 and 28°C (humidity not reported) [5]. The question remains whether this can be generalized, since adaptive thermal comfort was reported to change with seasonal outdoor temperatures [116]. Moreover, biological factors such as age, gender or ethnicity might be influential, as well as cultural habits (e.g. heating and cooling of indoor environments), and clients might become accustomed and conditioned to certain water and air temperatures over the course of their treatment series. Also, the interrelation of humidity and room temperatures could be of importance and might be worthwhile to report on in WATSU studies, as introduced by Chun et al., 2006, and Chon et al., 2009 [10, 13, 117]. Shortening WATSU sessions to 30 minutes due to receivers’ chilling in 32.7°C water temperature after 45 minutes was reported in the grey literature [66]. Consequently, multiple questions arise: whether an intervention at too low a temperature could or should still be considered “WATSU”, how essential the temperature (water and room) really is, what factors determine the “right” temperature and how temperature influences the treatment (from the first moment on, from when on).

Furthermore, timeframes of WATSU sessions when applied as stand-alone therapy varied in the assessed literature. A guiding rationale with respect to physiological changes (such as e.g. the relaxation response) that have been observed, or are assumed to have occurred or to have been satisfied after a certain period of time, seems to be lacking [118]. Also, guidelines concerning the duration of treatment series at given indications, or the frequency of WATSU sessions remain unclear. If there were a time necessary to integrate impressions, or to regenerate between one session and the next, this latency is not known. Dose-response issues are also a concern identified as a result of the meta-analyses, as they combine mere pre- and post-intervention values (short-term effects) with values assessed before and after entire treatment series. Only Ramirez et al., 2019, reported data from a longer (three-month) follow-up [51]. More studies with subsequent verification of the effectiveness and sustainability of the effects would be desirable.

In six studies, the topic of “side effects” was addressed, once positively (in a rehabilitation setting, an injured joint was once swollen after treatment). In general, one gets the impression that WATSU has virtually no negative or undesirable side effects, however, it will be essential to explore negative side effects and adverse events, as the applicability of WATSU in medical settings depends on this aspect.

This systematic review is limited by the quality of findings, as only seven of the studies assessed for risk of bias had control groups. The assessment revealed that not only the studies’ designs, but also poor reporting impeded accurate judgement in several studies. In subgroup analyses, however, the effect sizes of the meta-analyses were confirmed consistently with minimal heterogeneity, indicating that the inclusion of trials with lower methodological rigor led to an increase of noise without major over- or underestimation of the effect size as observed in RCTs. The weighted effect sizes provided in the current article warrant attempts to reproduce the reported results and may support future researchers in designing adequately powered RCTs about the effectiveness of WATSU.

### Summary, implications, and conclusions

In the literature retrieved in the present systematic review, WATSU was applied from childhood to advanced age. Indications included physical conditions (musculoskeletal, neurological) and mental issues (trauma, stress, depression, consciousness, emotional growth, spiritual experiences).

The implemented meta-analyses suggested beneficial effects of WATSU on pain, physical function, and mental issues. The level of this evidence is 1a (pain and physical function) and 1b (mental effects). Its quality according to GRADE was estimated to be low to moderate at the best since the number of study participants was small, and while the risk of bias was low in some of the analyzed studies, it was unclear in others due to study design or poor reporting. Nonetheless, considering the effect sizes and only one reported incident of negative side effects, WATSU can be confidently recommended for wellness purposes and cautiously for clinical applications in relation to the above-mentioned effects.

Investigations concerning the verification of the obtained results by experts could add further evidence. However, methodologically sound RCTs based on good clinical practice following recommended reporting guidelines to underpin frequency and magnitude of mentioned short- and long-term effects of WATSU will be needed in the future to determine WATSU’s proper position within the healthcare system. It is suggested that dose response, and responder profiles among patients with different pathologies as well as healthy individuals be considered. Dose-response issues are a concern identified as a result of the meta-analyses, as there are reports of mere pre- and post-intervention values (short-term effects) and values assessed pre-treatment series and post-treatment series. Furthermore, varying water temperatures and durations of both individual sessions as well as treatment series might depict practical reality, however, on behalf of comparability, gold standard definitions for WATSU (ideal setting, ideal timeframe) should be established.

The presented meta-analyses are suited to serve future researchers for designing trials and sample size calculations to further investigate the effect of WATSU on pain, physical function, and mental issues.

## Supporting information

S1 Checklist PRISMA 2009 Checklist. (PDF)

S1 Fig (TIF)

S2 Fig (TIF)

S3 Fig (TIF)

S1 Video (MP4)

S1 File (PDF)

S2 File (PDF)

S3 File (PDF)

S4 File (PDF)

S5 File (PDF)

S6 File (PDF)

S7 File (PDF)
