# Supplementary figures and images for: Applications, indications, and effects of passive hydrotherapy WATSU (WaterShiatsu)—A systematic review and meta-analysis
Source: PLoS One. 2020 Mar 13;15(3):e0229705. doi: 10.1371/journal.pone.0229705 (PMC7069616; doi:10.1371/journal.pone.0229705)

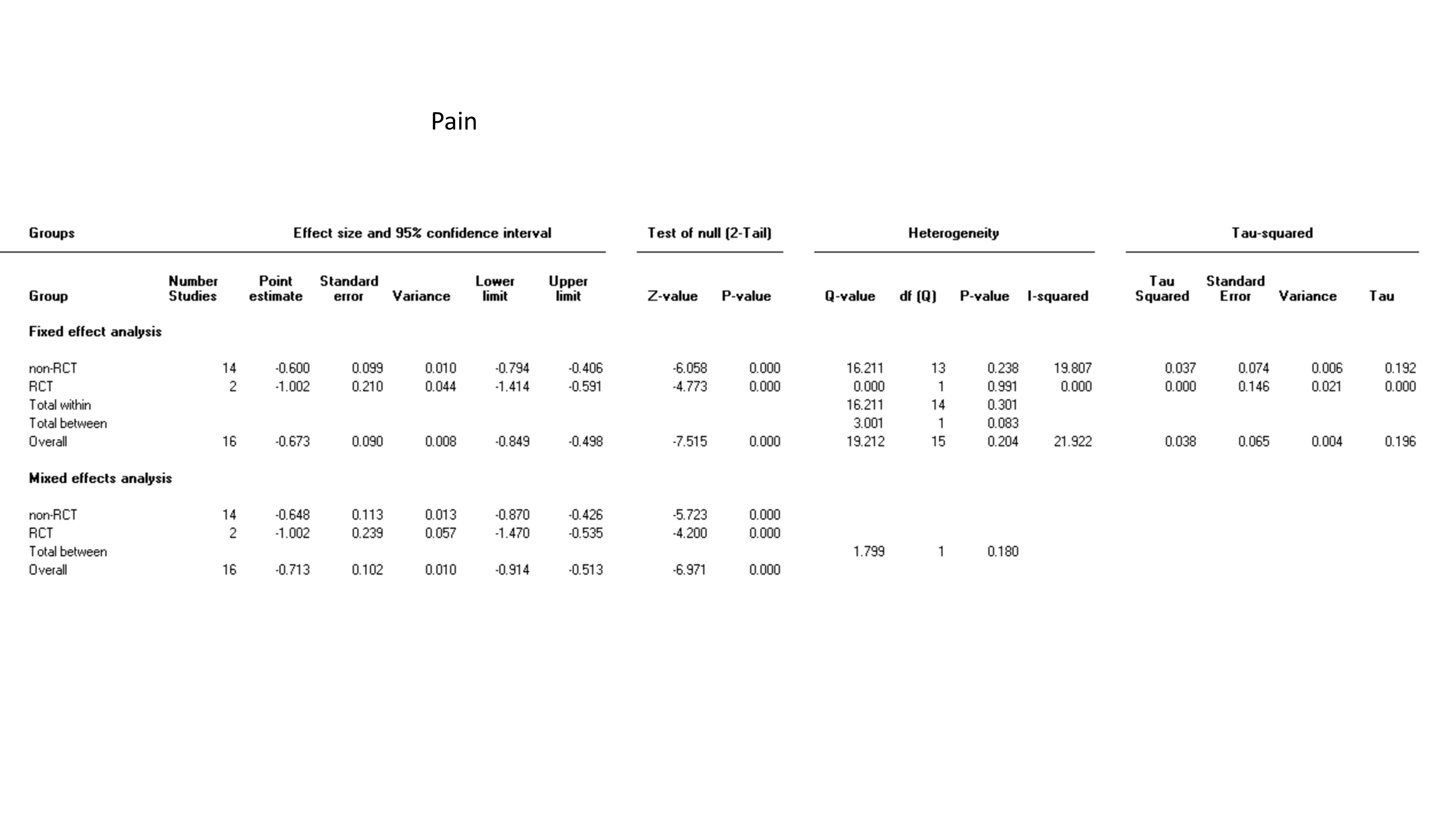

Supplement: S1 Fig — (TIF) [file pone.0229705.s002.tif]

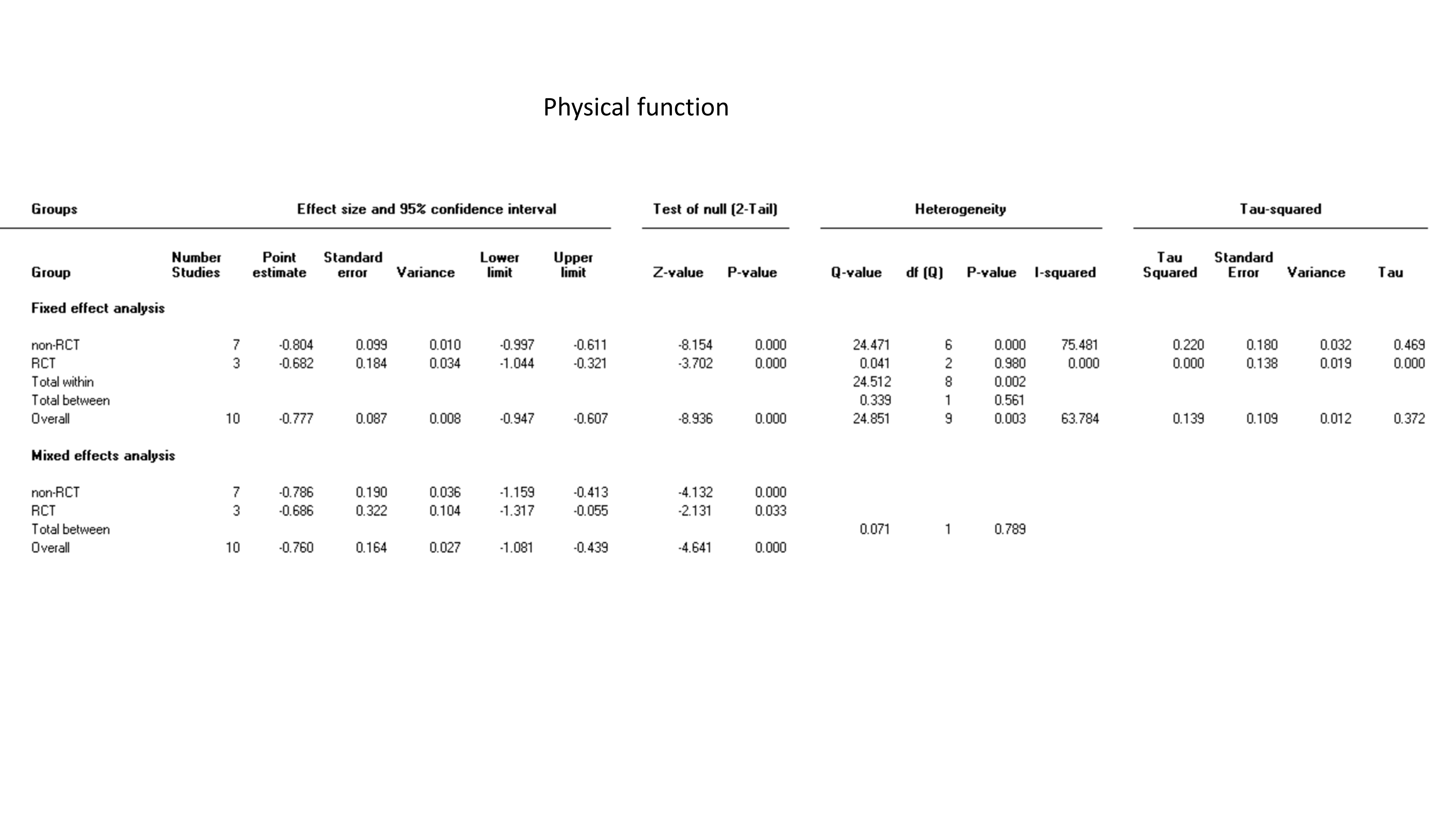

Supplement: S2 Fig — (TIF) [file pone.0229705.s003.tif]

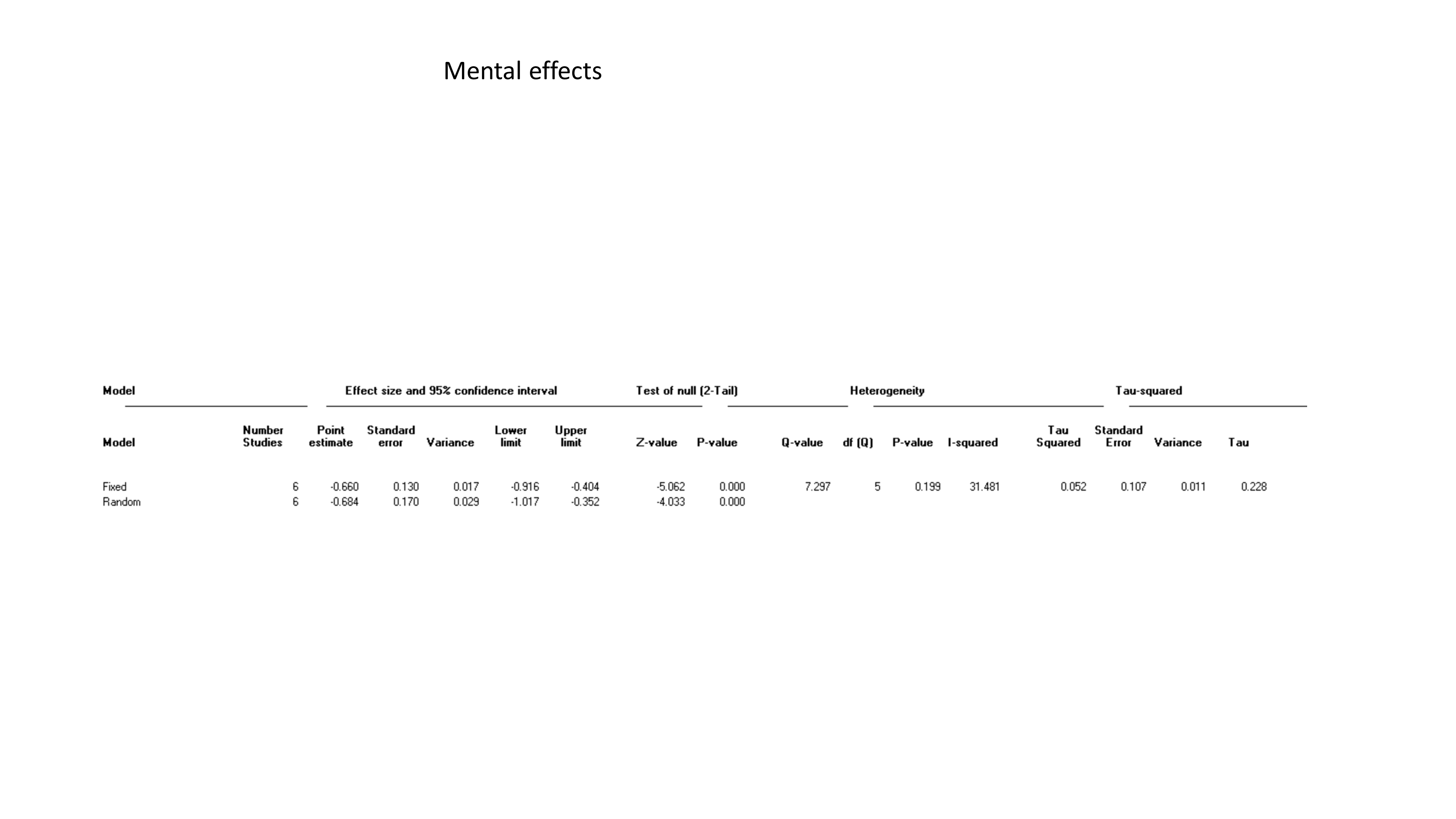

Supplement: S3 Fig — (TIF) [file pone.0229705.s004.tif]
