# Supplementary material for: Applications, indications, and effects of passive hydrotherapy WATSU (WaterShiatsu)—A systematic review and meta-analysis
Source: PLoS One. 2020 Mar 13;15(3):e0229705. doi: 10.1371/journal.pone.0229705 (PMC7069616; doi:10.1371/journal.pone.0229705)
Supplement: S1 File — (PDF) [file pone.0229705.s006.pdf]

Characteristics of studies

Characteristics of included studies

Antunes et al., 2016

**Methods**	Case series (CS)
**Participants**	17 female, 67.5 ±4.7 years old, clinical diagnosis of fibromyalgia. Elderly women with wheelchairs or those with waterborne diseases, open wounds, dermatological changes, abnormal blood pressure and any number of session absences were excluded.
**Interventions**	10 WATSU sessions in 5 weeks, 40 min.
**Outcomes**	pre and post intervention;<br>VAS pain: statistically lower in the second evaluation (p = 0.00059)<br>SF-36:<br>Evaluation -->Re-evaluation<br>Variables preMean/SD-->postMean/SD-->p<br>Physical function 38,2 ± 17,8 -->52,4 ± 21,3 -->0,00445*<br>Role physical 18,1 ± 23,2 -->45,6 ± 39,8 -->0,01347*<br>PAIN 28,8 ± 11,9 -->44,8 ± 12,8 -->0,00861*<br>General health 41,8 ± 17,6 -->47,0 ± 22,9 -->0,30663<br>Vitality 31,5 ± 20,4 -->53,5 ± 13,7 -->0,00044*<br>Social function 51,3 ± 27,4 -->61,0 ± 22,5 -->0,05037<br>Role emotional 23,5 ± 32,8 -->58,8 ± 41,7 -->0,02019*<br>Mental health 43,1 ± 16,9 -->59,1 ± 19,9 -->0,00748*<br>* P significant by wilcoxon test considering significance level of 5%.<br>At the beginning and at the end of each session, blood pressure was measured--> values not reported
**Notes**	39° C(trinta e nove graus Celsius!!), pH of 2.8, soft music.

Risk of bias table

**Bias**	**Authors' judgement**	**Support for judgement**
Random sequence generation (selection bias)	Unclear risk	n/a
Allocation concealment (selection bias)	Unclear risk	n/a
Analyzed within the group originally assigned to (selection bias)	Unclear risk	n/a
Comparable baseline outcome measurements (selection bias)	Unclear risk	n/a
Comparable baseline characteristics (selection bias)	Unclear risk	n/a
Control for important confounding (selection bias)	Low risk	criteria for in- and exclusion clearly stated

Blinding of participants and personnel (performance bias)	Unclear risk	insufficient information
Impact from a concurrent intervention (performance bias)	Unclear risk	insufficient information
Fidelity to the intervention protocol maintained (performance bias)	Low risk	apparently no deviations from protocol
Missing data handled appropriately (attrition bias)	High risk	3 dropouts, no Intention to treat reported "Elderly women with...any number of session absences were excluded."
Incomplete outcome data (attrition bias)<br>Objecitve Outcomes	Unclear risk	n/a
Incomplete outcome data (attrition bias)<br>Self-reported Outcomes	Unclear risk	insufficient information
Length of follow-up (detection bias)	Unclear risk	insufficient information
Blinding of outcome assessment (detection bias)	Unclear risk	insufficient information
Intervention clearly defined (detection bias)	Low risk	Detailed information about administered movements provided.
Outcomes assessed reliably (detection bias)	Low risk	VAS pain, validated,<br>SF-36 adapted and validated for Brazil
Confounding variables assessed (detection bias)	Unclear risk	insufficient information
Potential outcomes prespecified (reporting bias)	Low risk	prespecified outcomes declared
All prespecified outcomes reported (reporting bias)	High risk	At the beginning and at the end of each session, blood pressure was measured--> values not reported

Barbosa et al., 2014

**Methods**	Case report (CR)
**Participants**	1, female, 62 years, complaining of pain and fatigue in the area around the ear and no general body pain or fibromyalgia diagnoses. The patient also reported a high level of stress related to her daily activities and fatigue in an area of her face for more than a year as she wakes up, which is an indication of chronic pain. After clinical examination, it was observed pain to palpation of the masseter and anterior temporal and the presence of dental wear that associated to the patient’s report indicated a possible nocturnal parafunctional habit. RDC/TMD questionnaire verified that the patient sustained temporomandibular disorders and associated myofascial pain. "the patient was diagnosed with myofascial pain and in some way more susceptible to psychological problems as shown in this study."
**Interventions**	WATSU: 10 sessions, weekly, 40-60min

**Outcomes**	psychological aspects (anxiety and minor psychiatric disorders) and quality of life of a patient with temporomandibular disorders<br>pre-post (Series of treatment? Single treatment?):<br>Goldberg health questionnaire (GHQ) improvement: The second time the patient filled out the forms, there was a significant decrease of score values in the same dimensions, and the values were at normal levels Dying wish 2.36 to 1.27, Mental stress 2.91 to 1.30, Distrust in performance 2.95 to 1.25, Sleep disturbance 3.30 to 1.20, Psychosomatic disorders 3.38 to 1.38, General health 2.88 to 1.30<br>State-Trait Anxiety Inventory (STAI) decrease: Trait anxiety 64 to 34, State anxiety 50 to 32<br>WHOQOL-Brief (World Health Organization Quality of Life) increase: Physical domain 11 15, Psychological domain 12 to 14, Social domain 12 to 12, Environmental domain 11 to 14
**Notes**	Watsu therapy was effective in reducing anxiety and minor psychiatric disorders, as well as quality of life improvement of a patient with temporomandibular disorders, such as myofascial pain. 35 °

Risk of bias table

**Bias**	**Authors' judgement**	**Support for judgement**
Random sequence generation (selection bias)	Unclear risk	n/a
Allocation concealment (selection bias)	Unclear risk	n/a
Analyzed within the group originally assigned to (selection bias)	Unclear risk	n/a
Comparable baseline outcome measurements (selection bias)	Unclear risk	n/a
Comparable baseline characteristics (selection bias)	Unclear risk	n/a
Control for important confounding (selection bias)	Unclear risk	insufficient information
Blinding of participants and personnel (performance bias)	Unclear risk	n/a
Impact from a concurrent intervention (performance bias)	Unclear risk	insufficient information
Fidelity to the intervention protocol maintained (performance bias)	Low risk	apparently no deviations from protocol
Missing data handled appropriately (attrition bias)	Unclear risk	n/a

Incomplete outcome data (attrition bias)<br>Objecitve Outcomes	Unclear risk	n/a
Incomplete outcome data (attrition bias)<br>Self-reported Outcomes	Low risk	apparently no missing data
Length of follow-up (detection bias)	Unclear risk	n/a
Blinding of outcome assessment (detection bias)	Unclear risk	insufficient information
Intervention clearly defined (detection bias)	Low risk	35°C, 10 sessions, weekly, 40-60 min each. Detailed information about administered movements provided.
Outcomes assessed reliably (detection bias)	Low risk	RDC/TMD questionnaire. This questionnaire is a valid and reproducible instrument used worldwide for TMD diagnosis<br>WHOQOL-Brief: validated<br>STAI: validated<br>Goldbergs General Health Questionnaire: validated
Confounding variables assessed (detection bias)	Unclear risk	insufficient information
Potential outcomes prespecified (reporting bias)	Low risk	prespecified outcomes declared
All prespecified outcomes reported (reporting bias)	Low risk	all outcomes prespecified in methods-section reported

Borges & Parizotto, 2001

**Methods**	CS
**Participants**	18, mainly female, 20-60 years, various professions and schooling from first degree to upper course. 72% not performing physical exercise but having a workload of at least 8 hours. 55% complained of pain in the cervical region causing limitations in the rotation of the neck, abduction and external rotation of the shoulder. 94% of the patients complained of pain with intensity varying from 4 to 10 (average 6.9); 62% of them had already seen physiotherapists with unsatisfactory results. stress and pain
**Interventions**	5 times WATSU (time period NOT mentioned) for 50 minutes
**Outcomes**	Graphs blurred!!! Cannot be interpreted!!! Figure 4 missing!!!<br>blood pressure, frequency, heart rate and breathing, respiratory rate, questionnaire, VAS pre/post.<br>It was verified in the monitoring of arterial pressures, demonstrated by Figure 1, that few alterations occurred. Based on previous data on patients' blood pressure control, it was observed that the treatment favored the approximation of the normal pressure values of each experimental patient, in some cases encouraging the elevation of systolic pressure to 120 mmHg and diastolic pressure to 80 mmHg. Analyzing the data individually, it was observed that none of the participants presented hypertension, one with hypotension; no change was observed during the treatment.<br>According to Skinner [10], water heated to average values does little to interfere

	with blood pressure, and variations may occur in cases of hypertension. However, in the monitoring of respiratory frequencies, shown in figure 2, the tendency to decrease after the hydrotherapy sessions is observed. Such fact can be justified by the respiratory work performed at the beginning of the sessions, leading to a vasodilation produced by immersion in heated water. The respiratory rhythm check was performed inside the pool immediately after the sessions.<br><br>The heart rate also presented changes, reducing after the treatment as shown in Figure 3. The initial mean was 84 beats per minute (bpm) and the final 78 bpm, which equals less 6 bpm, representing a reduction Significant. Patients who presented heart rates above 90 bpm were smokers and pregnant, and there was change only in the case of smokers.<br><br>In the final questionnaire, pain was evaluated by the visual analogue scale, and figure 4 shows a significant decrease in the pain intensity of the patients after the treatment period, ranging from 0 to 5, mean was 2.2. Comparing the initial pain intensity that reached the mean of 6.9 with the final of 2.2, a considerable result is observed in the reduction of pain. It was observed that patients with joint limitation reported pains during the first sessions, and less in the fourth and fifth sessions. The reduction of pain associated with daily life guidelines and emotional balance contributed to a higher work performance, as reported by 95% of the participants.<br>At the end of the experimental sessions, 45% of participants decided to continue treatment.<br>Good sleep quality scores were obtained, of which 72% felt improved and 23% remained unchanged. At the end of each session, the patients reported that they were sleepy and that they wanted to stay in the pool until they slept.<br><br>In the applied questionnaire, it was observed, besides the decrease of the pain, information as the increase of the income in the work and improvement of the sleep. Watsu promotes greater flexibility, serenity, less fatigue, tension relief, willingness to perform activities of daily living, mood enhancement, irritability control, and some have verbally reported that Watsu benefits the body, mind and soul.
**Notes**	34-36 °C

Risk of bias table

Bias	Authors' judgement	Support for judgement
Random sequence generation (selection bias)	Unclear risk	n/a
Allocation concealment (selection bias)	Unclear risk	n/a
Analyzed within the group originally assigned to (selection bias)	Unclear risk	n/a

Comparable baseline outcome measurements (selection bias)	Unclear risk	n/a
Comparable baseline characteristics (selection bias)	Unclear risk	n/a
Control for important confounding (selection bias)	Unclear risk	insufficient information
Blinding of participants and personnel (performance bias)	Unclear risk	insufficient information
Impact from a concurrent intervention (performance bias)	Unclear risk	insufficient information
Fidelity to the intervention protocol maintained (performance bias)	High risk	timeframe of trial not reported
Missing data handled appropriately (attrition bias)	Unclear risk	insufficient information
Incomplete outcome data (attrition bias)<br>Objecitve Outcomes	Unclear risk	insufficient information
Incomplete outcome data (attrition bias)<br>Self-reported Outcomes	Unclear risk	insufficient information
Length of follow-up (detection bias)	Unclear risk	"at the beginning and end of each session" Parameters such as blood pressure and Respiratory rate were recorded. Standardized? In or out of the water? Time-frame of entire exposure unclear (some participants reported increase in income). Insufficient information.
Blinding of outcome assessment (detection bias)	Unclear risk	insufficient information
Intervention clearly defined (detection bias)	Low risk	yes, 5, 50min each, 34-36°C; detailed information about which movements were employed for how long etc.
Outcomes assessed reliably (detection bias)	Low risk	yes, blood pressure, frequency, heart rate and breathing, respiratory rate, questionnaire (VAS) pre/post: "At the beginning and end of each session" In or out of the water?
Confounding variables assessed (detection bias)	Unclear risk	"Work with Watsu was not associated with classical physiotherapy and many patients stopped using drugs (analgesics and tranquilizers) during the treatment period."
Potential outcomes prespecified (reporting bias)	Low risk	prespecified outcomes declared
All prespecified outcomes reported (reporting bias)	High risk	Table 4 (VAS pain) is mentioned, yet not depicted in the article. All figures are blurred to illegibility.

Campos et al., 2018

**Methods**	CS
**Participants**	11, female 18 to 65 years, employees of the various sectors of an University Hospital for at least one year, with a minimum workload of 20 hours per week on site; who had complaints of stress, difficulty sleeping and pain, linked to work in the hospital. Women who had contraindications to entry into the pool (dermatological lesions, urinary or fecal incontinence), with vestibular diseases or diseases that could compromise treatment in water; fear of water or history of trauma, or that did not fit the first session of technique recognition, were excluded.
**Interventions**	6 times WATSU in 6 weeks. Detailed description provided.
**Outcomes**	Improvement in systolic blood pressure (SBP), diastolic blood pressure (DBP), heart rate (HR), pain intensity, and flexibility:<br>SBP (mmHg) pre 116 (SD 1.7224) -->post 111 (SD 2,7386 0), p = 0.0058*,<br>DBP (mmHg) 73 (SD 2.0736) -->71 (SD 1.9664), p = 0.0407*,<br>HR (bpm) 82 (SD 4.0332) -->71 (SD 1.9408), p <0.0001*,<br>and pain intensity (p = 0.0005) (cm) 2,7 (SD 0.5164) -->1,0 (SD 0.9174), p = 0.0005*,<br>as well as improvement in the average flexibility (p = 0.0035) --> (cm) 11 (SD 1.8348) -->9 (SD 1.2111), p = 0.0035*
**Notes**	34° C, 45 min. "There was no interruption due to discomfort during the sessions."

Risk of bias table

**Bias**	**Authors' judgement**	**Support for judgement**
Random sequence generation (selection bias)	Unclear risk	n/a
Allocation concealment (selection bias)	Unclear risk	n/a
Analyzed within the group originally assigned to (selection bias)	Unclear risk	n/a
Comparable baseline outcome measurements (selection bias)	Unclear risk	n/a
Comparable baseline characteristics (selection bias)	Unclear risk	n/a
Control for important confounding (selection bias)	Low risk	criteria for in- and exclusion clearly stated
Blinding of participants and personnel (performance bias)	Unclear risk	insufficient information
Impact from a concurrent intervention (performance bias)	Unclear risk	insufficient information
Fidelity to the intervention protocol maintained (performance bias)	Low risk	apparently no deviations from protocol

Missing data handled appropriately (attrition bias)	High risk	"six did not start treatment due to unavailability of time and two interrupted treatment after two sessions of aquatic relaxation, also due to unavailability of time" --> no intention to treat reported
Incomplete outcome data (attrition bias)<br>Objecitve Outcomes	Unclear risk	insufficient information
Incomplete outcome data (attrition bias)<br>Self-reported Outcomes	Unclear risk	insufficient information
Length of follow-up (detection bias)	Unclear risk	n/a
Blinding of outcome assessment (detection bias)	High risk	"[...] the examiner, being the same responsible for the intervention."
Intervention clearly defined (detection bias)	Low risk	Detailed description provided.
Outcomes assessed reliably (detection bias)	Low risk	"Job Stress Scale (Alves et al., 2004), BP, HR, mapping of three most painful points, their respective pain intensities (VAS) and checking flexibility (the distance between the tip of the third finger and the floor, in which the patient remains standing with knees extended and arms and hands extended toward the floor in parallel and tilts his trunk forward) were verified at the beginning and end of the study."<br>"After five minutes of rest in a calm environment, BP was measured according to the recommendations of the Brazilian Society of Cardiology. HR per minute was measured by a pulse frequency meter following the BP in the pool outside (at rest), as well as at the end of the session (intervention effect) in the sitting position. Thus, the variables used for analysis of this study (SBP and DBP, HR, flexibility and pain intensity) were verified before and after each of the six relaxation sessions."
Confounding variables assessed (detection bias)	Unclear risk	insufficient information
Potential outcomes prespecified (reporting bias)	Low risk	prespecified outcomes declared
All prespecified outcomes reported (reporting bias)	Low risk	all outcomes prespecified in methods-section reported

Chen et al., 2018

**Methods**	CT
**Participants**	20 (10:10) Fibromyalgia patients, pain for at least two years, age 40-75 yrs. The study did not include women who had reservations and avoided staying in the water.

**Interventions**	intervention: 30 min WATSU, weekly, 8 weeks<br>control: 45 min instructed aquatic avtivities, weekly, 8 weeks
**Outcomes**	Sleep quality was evaluated using the Pittsburgh Sleep Quality Index (PSQI) questionnaire and the severity of pain was expressed using the 1-10 Visual Analogue Scale (VAS). Watsu treatments significantly reduced pain while sitting, walking,<br>driving, standing and lying, and it improved sleep quality in fibromyalgia patients. This beneficial effect was not observed in the group that participated in instructed aquatic activities.
**Notes**	34-35 °C duration of interventions: 30 min versus 45 min

Risk of bias table

Bias	Authors' judgement	Support for judgement
Random sequence generation (selection bias)	Unclear risk	n / a
Allocation concealment (selection bias)	Unclear risk	n / a
Analyzed within the group originally assigned to (selection bias)	Unclear risk	insufficient information
Comparable baseline outcome measurements (selection bias)	Unclear risk	data presented for VAS only
Comparable baseline characteristics (selection bias)	Unclear risk	not reported!
Control for important confounding (selection bias)	Unclear risk	insufficient information
Blinding of participants and personnel (performance bias)	Unclear risk	insufficient information
Impact from a concurrent intervention (performance bias)	Unclear risk	insufficient information
Fidelity to the intervention protocol maintained (performance bias)	Unclear risk	insufficient information
Missing data handled appropriately (attrition bias)	Unclear risk	insufficient information
Incomplete outcome data (attrition bias)<br>Objecitve Outcomes	Low risk	n / a
Incomplete outcome data (attrition bias)<br>Self-reported Outcomes	Unclear risk	insufficient information

Length of follow-up (detection bias)	Low risk	Patients were instructed to complete the questionnaires before going to bed, as follows: two nights prior to the scheduled treatment, on the eve of the scheduled treatment, and in the evening post-treatment, in every week.
Blinding of outcome assessment (detection bias)	Unclear risk	insufficient information
Intervention clearly defined (detection bias)	Low risk	Group 1) Watsu (30 minutes / once a week) The patient remained in a horizontal position in the water as she was passive and supported by the therapist in various areas of her body, giving slow movements during treatment and demanding deep breathing a feeling of calm and relaxation.<br>Group 2) Water-guided activity (45 minutes / once a week) The activity included exercises to strengthen muscular strength, improve joint mobility, balance and aerobic activity, and various aids such as elastic bands and palms were used to increase resistance<br>increase, as well as surfboards for balance.
Outcomes assessed reliably (detection bias)	High risk	Pittsburgh Sleep Quality Index (PSQI): valide for changes in 4 weeks, VAS pain
Confounding variables assessed (detection bias)	Unclear risk	insufficient information
Potential outcomes prespecified (reporting bias)	Low risk	apparently
All prespecified outcomes reported (reporting bias)	High risk	Questionnaires were answered weekly, yet only 1st and last week were reported. Results not properly reported: When considering baseline as first value, VAS-values in WATSU-group deteriorated in walking, drivin and standing at day1 (sum score of all 5 conditions: 6.33 at baseline to 6.32 at day1), while they improved in control-group (from 7.06 to 6.24). From baseline to day8, WATSU-group performed better than the control group (from 6.33 to 4.81 and from 7.06 to 6.08, resprectively). Adding these values, the control group performed slightly better (difference pre-post .0.90) than the WATSU-group (difference pre-post 0.76). However, the values of the days 2 to 7 are not presented, thus, this calculation does not reflect the full truth.<br>Baseline of PSQI not reported.

Chon et al., 2009

**Methods**	CS
**Participants**	3 patients with hemiplegia 8-20 months after stroke, 49-62 years
**Interventions**	Watsu consisted of 40 treatment sessions for 8 weeks (five times a week), 40 minutes each

**Outcomes**	tone assessment scale (TAS):physical assessment of muscle tone, composed of items assessing posture at rest, response to passive movement and associated response to active movement attempt (ranging from 0 (no muscle tone) to 40 (severe muscle tone)<br>Rivermead Visual Gait Assessment (RVGA):total score can be calculated by summing the total numbers of deviation scores, ranging from 0 (normal gait) to 59 (grossly abnormal gait)<br>Improvements in spasticity and ambulatory function after the Watsu approach were shown:<br>tone assessment scale scores for all three patients assessed before/after WATSU: before: 21 16 28 after: 9 5 10);<br>rivermead visual gait assessment scores for all three patients assessed before/after WATSU: before: 21 15 34 after: 9 7 10.<br>Measurements were performed before the initiation and after the completion of Watsu session.
**Notes**	Temperature: 93.2 °F

Risk of bias table

Bias	Authors' judgement	Support for judgement
Random sequence generation (selection bias)	Unclear risk	n/a
Allocation concealment (selection bias)	Unclear risk	n/a
Analyzed within the group originally assigned to (selection bias)	Unclear risk	n/a
Comparable baseline outcome measurements (selection bias)	Unclear risk	n/a
Comparable baseline characteristics (selection bias)	Unclear risk	n/a
Control for important confounding (selection bias)	Unclear risk	insufficient information
Blinding of participants and personnel (performance bias)	Unclear risk	insufficient information
Impact from a concurrent intervention (performance bias)	Unclear risk	insufficient information
Fidelity to the intervention protocol maintained (performance bias)	Low risk	apparently no deviations from protocol
Missing data handled appropriately (attrition bias)	Low risk	full data sets provided, only descriptive statistics
Incomplete outcome data (attrition bias)<br>Objecitve Outcomes	Low risk	data compared in a reasonable way

Incomplete outcome data (attrition bias)<br>Self-reported Outcomes	Unclear risk	n/a
Length of follow-up (detection bias)	Low risk	standardized
Blinding of outcome assessment (detection bias)	High risk	objective outcomes seem to have been evaluated by therapist, no sufficient information otherwise
Intervention clearly defined (detection bias)	Low risk	34°C, Watsu consisted of 40 treatment sessions for 8 weeks (five times a week), delivered underwater or at water surface level. Detailed description provided.
Outcomes assessed reliably (detection bias)	Low risk	TAS: Acceptable levels of agreement were not achieved for items reflecting posture at rest and associated reactions<br>RVGA: is both a valid and sensitive measure of gait impairment in patients with neurological disease (Lord et al., 1998)
Confounding variables assessed (detection bias)	Unclear risk	insufficient information
Potential outcomes prespecified (reporting bias)	Low risk	prespecified outcomes declared
All prespecified outcomes reported (reporting bias)	Low risk	all outcomes prespecified in methods-section reported

Chun et al., 2006

**Methods**	RCT; single subject alternating design (cross)
**Participants**	3 Stroke patients with hemiplegia for 23-31 months, 55-64 years old
**Interventions**	Each subject alternately participated several times in three conditions: a range of motion exercises on the mat and in the pool, and relaxation exercises (WATSU) in the pool for 40 minutes
**Outcomes**	the tone assessment scale (TAS):physical assessment of muscle tone, composed of items assessing posture at rest, response to passive movement and associated response to active movement attempt<br>The findings showed a therapeutic effect of submerged relaxation exercise on reducing spasticity for all subjects. The effect of submerged relaxation exercise on decreasing muscle tone was maintained for one hour measurements after the submerged relaxation exercise, although the mean TAS score assessed one hour after intervention was higher than that assessed immediately.<br>TAS: 0 (no muscle tone) to 40 (significant muscle tone); The changes in TAS scores of baseline and treatment duration of the subjects are shown in Table 2, Figure 1 and Figure 2. The baseline average score of the subject 1 was 12.67 points, but the average score at the time of performing the range of motion exercise on the mat was 9.40 and the average score after 1 hour was 11.13 points. The average score immediately after the procedure was 7.80 and the average score after 1 hour was 9.80. The average score immediately after the underwater relaxation exercise was 5.67 and the mean score after 1 hour was 7.93. The measured values after the test showed lower tendency than the measured values of the baseline. The mean score of the baseline score of the

	subject 2 was 18.12 points, but the average score immediately after performing the range of motion exercise on the mat was 14.92 and the average score after 1 hour was 16.58 points. The average score at the end of the exercise session was 10.25 and the mean score at the end of the exercise session was 12.00. The mean score at the end of the exercise session was 12.25 Values were lower than baseline measurements. The baseline average score of the subjects 3 was 17.44, but the mean score at the time of performing the range of motion exercise in the mat was 15.11 and the mean score at the 1 hour after the exercise was 16.67 points. The mean score immediately after the procedure was 12.67 and the mean score after 1 hour was 14.33. The mean score immediately after the hydrodynamic exercise was 9.33 and the mean score after 1 hour was 12.78. Subsequent measurements showed a lower tendency than baseline measurements.<br><br>All subjects showed lower scores in underwater exercises than in matt exercises, with the lowest scores in aquatic relaxation exercises (WATSU).<br>The scores measured at 1 hour after treatment were higher than those measured immediately after treatment. Points measured at 1 hour after treatment showed lower values of water relaxation among three treatment methods.
**Notes**	Temperature: 34 °C, time-frame of treatment-series not reported

Risk of bias table

Bias	Authors' judgement	Support for judgement
Random sequence generation (selection bias)	Unclear risk	insufficient information
Allocation concealment (selection bias)	Unclear risk	insufficient information
Analyzed within the group originally assigned to (selection bias)	Low risk	apparently
Comparable baseline outcome measurements (selection bias)	Low risk	yes (cross design, each person experienced all conditions and was compared with herself)
Comparable baseline characteristics (selection bias)	Low risk	yes
Control for important confounding (selection bias)	Unclear risk	order of treatments not reported
Blinding of participants and personnel (performance bias)	Low risk	In order to reduce the pollution variables by the researchers, the treatment and measurement of the subjects were consistently conducted by different researchers during the experiment.
Impact from a concurrent intervention (performance bias)	Unclear risk	insufficient information
Fidelity to the intervention protocol maintained (performance bias)	High risk	timeframe of trial not reported

Missing data handled appropriately (attrition bias)	Low risk	apparently no missing data
Incomplete outcome data (attrition bias)<br>Objecitve Outcomes	Low risk	apparently no missing data
Incomplete outcome data (attrition bias)<br>Self-reported Outcomes	Unclear risk	n/a
Length of follow-up (detection bias)	Low risk	"All measurements were performed immediately after the end of each exercise and were performed in an evaluation room located in the veterinary room"
Blinding of outcome assessment (detection bias)	Unclear risk	insufficient information
Intervention clearly defined (detection bias)	Unclear risk	yes, detailed information about all three involved conditions presented; no: time-frame not reported, not clear, whether the three interventions were on the same day or in the same week.
Outcomes assessed reliably (detection bias)	Low risk	yes
Confounding variables assessed (detection bias)	Unclear risk	insufficient information
Potential outcomes prespecified (reporting bias)	Low risk	prespecified outcomes declared
All prespecified outcomes reported (reporting bias)	Low risk	all outcomes prespecified in methods-section reported

Cunha et al., 2010

**Methods**	CS: Experimental study without control
**Participants**	30 healthy women, not familiar with WATSU
**Interventions**	1 session of WATSU each, 40min
**Outcomes**	medium blood pressure (BP), double product,<br>variations in cardiac heart rate (HR),<br>flexibility F1 (photogrammetric method),<br>and verbal report (VR)<br>4 open questions about: perceived time of session, sensations experienced during Watsu, feelings right after the sessions, interest about joining a program of 10 sessions of WATSU.<br>Results: statistical significant difference in the medium of BPM double product and HR between the moments in soil, before and soon after the immersion and, soon after and five minutes after the immersion and, before and after the intervention indicating that intervention didn't mean physical effort and provided relaxation. It was gets better of flexibility. The average differences of MAP, DP and FC variation between the soil moments before the immersion and soon after the immersion were, respectively, 8.6%, - 12.4% and - 8.9% (all with p <0.005).

	The comparison between the measurements collected five minutes after the immersion showed that the values tended to approximate the measurements collected before the immersion in the soil. Significant effects of immersion in the cardiovascular system, as expected as described in the literature. The mean difference in MAP, PD, HR between the before and after moments of the intervention were, respectively, 3.7%, - 1.4%, - 2.9% (with p <0.005), demonstrating that the intervention did not mean any physical effort for the individuals and still provided a small but significant reduction of these parameters.<br><br>The mean difference of variation of MAP, SD, HR between the moments at rest before and after intervention, collected in the soil, were, respectively, 2.1%, - 0%, - 0.9% (all with p> 0.005), demonstrating that there was no statistically significant alteration in these parameters, due to the immersion and the associated intervention, that is, the values after the intervention returned to basal values.<br>The mean difference between the variation of the right external malleolus distance and the styloid process of the right ulna before and after the intervention was? 21.4%, with p <0.005, indicating that the Watsu session affected the flexibility providing an average decrease of 15.2 ± 7.8 cm in the distance evaluated.<br>The Watsu session, which lasted 36 ± 2 minutes, was perceived to last approximately 50 minutes per 8 (26.5%) of the 30 participants, lasting one hour for 10 (33.5%) of the 30 women, As having lasted over an hour for 12 people (40%).<br><br>Verbal feedback not assessed by means of an established procedere (questions used before in similar trial), however, comprehensible analysis: "The analysis of the reports obtained with the application of the questionnaire was evaluated after its organization into categories and expressed in the form of absolute and relative frequency, except for the first question that was related to perceived time of intervention." (comparable to the Mayring Triangulation Model). 1. How long have you believed the session lasted? 2. Discuss the feelings, situations, feelings and thoughts experienced during the Watsu session. 3. How do you feel at the moment (after the session)? 4. Are you interested in participating in a 10 session Watsu program? Regarding the second question 2 (Talk about the sensations experienced during the Watsu session), four categories of response were found: relaxation of the mind, relaxation of the body, sleepiness or sleep and well being, being the frequency of absolute responses, respectively 24, 27, 6, and 27. Each participant cited at least one of the categories and a maximum of three. Most reported relaxation of body and mind associated with the sense of well-being (25 participants - 80%). The fact that six women (20%) slept was clinically indicative of deep relaxation. There was no report of discomfort in relation to the session. Concerning the third question 3 (How do you feel at the moment, after the session), you found 7 categories of answers: Is the frequency of absolute responses, respectively of 21, 16, 21, 14, 8, 11, 23. The most mentioned behaviors - tranquility, lightness and stretching - are Indicative of relaxation and improvement in muscular tension level. In the last question, when asked if there would be interest in participating in a program of 10 sessions of Watsu, all 30 participants answered yes, and of these, 19 mentioned that they were interested in the technique and that the willingness to undergo a treatment would be in the Dependence on cost, although it has never been touched on the subject matter payment.Question nr.2 brought forward 4 categories: relaxation of mind,

	relaxation of body, drowsiness or sleep and well-being. Question nr.3 brought forward 7 categories: happy, calm, rested, light, sleepy, centered, elongated. Each participant told at least a state of relaxation and satisfaction.
**Notes**	Temperature: 35 ºC

Risk of bias table

Bias	Authors' judgement	Support for judgement
Random sequence generation (selection bias)	Unclear risk	n/a
Allocation concealment (selection bias)	Unclear risk	n/a
Analyzed within the group originally assigned to (selection bias)	Unclear risk	n/a
Comparable baseline outcome measurements (selection bias)	Unclear risk	n/a
Comparable baseline characteristics (selection bias)	Unclear risk	n/a
Control for important confounding (selection bias)	Low risk	The use of the variation calculation makes it possible to compare the data in a group of patients despite differences in age; inclusion and exclusion criteria clearly stated, same hour for all patients (9:00-10:00); absence of musculoskeletal, neuromotor or cardiovascular dysfunction assessed by functional examination performed by experienced physiotherapist
Blinding of participants and personnel (performance bias)	Unclear risk	insufficient information. Data were collected by physiotherapist specifically trained for this function.
Impact from a concurrent intervention (performance bias)	Low risk	PA and HR were measured after five minutes of rest in the sitting posture in the pool area (at rest) after water immersion until the xiphoid process (Effect of immersion), after five minutes in immersion (adaptation to immersion, and baseline measurement to evaluate the effect of the intervention) at the end of the session (effect of the intervention), in the pool with water until the xiphoid process and logo.<br>After leaving the pool, in the sitting position (return to normal). This collection routine was described by CANDELORO (2007) and allows to distinguish the changes caused by the immersion caused by the intervention.
Fidelity to the intervention protocol maintained (performance bias)	Low risk	apparently no deviations
Missing data handled appropriately (attrition bias)	Low risk	insufficient information, but attrition is not likely an issue in a single session pre-post-design

Incomplete outcome data (attrition bias)<br>Objecitve Outcomes	Low risk	apparently no missing data
Incomplete outcome data (attrition bias)<br>Self-reported Outcomes	Low risk	apparently no missing data
Length of follow-up (detection bias)	Low risk	standardized
Blinding of outcome assessment (detection bias)	Unclear risk	insufficient information
Intervention clearly defined (detection bias)	Low risk	session: average of 36 ± 2 minutes. Details about involved maneuvers provided. Temperature: 35 °C
Outcomes assessed reliably (detection bias)	Low risk	Verbal feedback not assessed by means of an established procedere (questions used before in similar trial), however, comprehensible analysis: "The analysis of the reports obtained with the application of the questionnaire was evaluated after its organization into categories and expressed in the form of absolute and relative frequency, except for the first question that was related to perceived time of intervention." (Triangulation Model, Mayring?). blood pressure parameters, heart rate, flexibility. Flexibility, assessed using the photographic test of the anterior flexion of the trunk a from sitting, or seat and reach test. The photograph was taken with a 5.0-inch Sony® digital still camera and the Distance measurement obtained using CAD 2000 software (CANDELORO;CAROMANO, 2007). For this, anatomical reference points (right external malleolus and styloid process of the right ulna) were performed and the flexibility test was performed with photographic image collection before and after the session (WATSON, 1998). The right external malleolus and styloid process of the right ulna were measured. The data collection was performed by trained physiotherapist (CAROMANO et al., 1995, TAKAHASHI et al., 1995).
Confounding variables assessed (detection bias)	Low risk	"The use of the variation calculation makes it possible to compare the data in a group of patients despite differences in age."
Potential outcomes prespecified (reporting bias)	Low risk	prespecified outcomes declared
All prespecified outcomes reported (reporting bias)	Low risk	all outcomes prespecified in methods-section reported

Dornelas, 2011

**Methods**	CR
**Participants**	1 patient, 23 years, with Jarcho-Levin syndrome after corrective surgery of the spine / paraplegia
**Interventions**	15 sessions of Watsu (3 times a week, 5 weeks, 40min), after that sessions of Hydrokinesioterapy and then Halliwick, in succession
**Outcomes**	"We obtained measures of range of motion, flexibility (goniometer), muscle strength and sensitivity in lower limbs (ASIA-Scale),<br>modified Ashworth scale for muscle tone, activities of daily life (ADL) questionnaire with MIF-Questionnaire (functional independence measure, MIF) in18 items about personal care, sphincter control, mobility, locomotion,communication and social cognition.<br>RESULTS of WATSU-phase:<br>In the pre-treatment, MIF accounted for 57.1%, with the lowest scores being in basic daily activities (bathing, dressing room, transfers and locomotion), and in the clinical examination, the values were obtained Of: Range of Motion (ADM - active) by goniometry: 75º and 55º in flexion of the hips / knees, respectively;<br>Grade 2 on lower limbs of Muscle Tone by the Modified Ashworth Scale;<br>Sensitivity change (grade 1) and Muscular Strength (grade 2 in the main muscle groups of the lower limbs), according to the ASIA scale;<br>Pain in the column (4 + / 5 +).<br>In the first sessions, hydrotherapy prioritized pain relief, since it was the factor that limited the patient to the other activities. The sessions were based on the Watsu technique with the purpose of promoting relaxation, muscular re-education and ventilation, through stretches performed in Zen Shiatsu. Thus, after 15 sessions, MIF was reapplied and the patient achieved a significant improvement, scoring 62.7% (scoring more in relation to the transfers and assisting more in the locker room).<br>She presented pain reduction (2 + / 5 +), which allowed her to perform independently in the postural changes (decubitus changes) and began to sit with support for 10 minutes.<br>The Watsu technique was chosen primarily because of what it provides and what the patient Presented initially. This method, which is based on stretches performed in Zen Shiatsu, has allowed for relaxation and ventilation re-education and, consequently, pain relief. Subsequently, the use of Hydrokinesiotherapy contributed to the improvement of the patient's functional capacity, such as, postural changes, WMD and muscular strength."
**Notes**	Temperature: 34 ºC, Author was contacted to clarify data (with no response)

Risk of bias table

**Bias**	**Authors' judgement**	**Support for judgement**
Random sequence generation (selection bias)	Unclear risk	n/a
Allocation concealment (selection bias)	Unclear risk	n/a

Analyzed within the group originally assigned to (selection bias)	Unclear risk	n/a
Comparable baseline outcome measurements (selection bias)	Unclear risk	n/a
Comparable baseline characteristics (selection bias)	Unclear risk	n/a
Control for important confounding (selection bias)	Unclear risk	insufficient information
Blinding of participants and personnel (performance bias)	Unclear risk	n/a
Impact from a concurrent intervention (performance bias)	Unclear risk	insufficient information
Fidelity to the intervention protocol maintained (performance bias)	Low risk	apparently
Missing data handled appropriately (attrition bias)	Unclear risk	n/a
Incomplete outcome data (attrition bias)<br>Objecitve Outcomes	Low risk	apparently no missing data
Incomplete outcome data (attrition bias)<br>Self-reported Outcomes	Low risk	apparently no missing data
Length of follow-up (detection bias)	Unclear risk	n/a
Blinding of outcome assessment (detection bias)	Unclear risk	insufficient information
Intervention clearly defined (detection bias)	Low risk	15 sessions of WATSU, 34°C
Outcomes assessed reliably (detection bias)	Low risk	valid and reliable measures used;<br>however: unclear how pain was assessed.
Confounding variables assessed (detection bias)	Unclear risk	insufficient information
Potential outcomes prespecified (reporting bias)	High risk	"Pain" is not mentioned in the methods section of the paper, it is also unclear which scale was employed for this parameter, yet it was reported
All prespecified outcomes reported (reporting bias)	High risk	Several outcomes not reported for each phase of treatment. MIF-Score: only 5 items with the lowest grades pre-treatment are reported (out of 18 possible items).

Faull, 2005

**Methods**	RCT: cohort study, cross-over design
**Participants**	17 females diagnosed with Fibromyalgia, 13 completed study, only those analysed: 26 to 65 years, mean of 46.3 (SD = 12.27) years. Average length of time with FMS was 4.3 years (SD = 1.3). Participants resided in the Bay of Plenty, East Coast, Hawkes Bay, Taranaki and Auckland regions of New Zealand and had received secondary school level qualifications, three had university qualifications. Main occupations were predominantly home and/or childcare, but four participants were employed as professionals in various fields
**Interventions**	Each treatment block consisted of four sessions over 2 weeks, comprising of two sessions per week with a 2-day gap between sessions. The first treatment block was followed by 3 weeks of no treatment before commencement of the second treatment block, 45min. Sufficient information provided to recognize the therapy.
**Outcomes**	MOS SF-36 physical function, bodily pain, vitality, social function, role physical, general health, role emotional, mental health<br>Means and standard deviations of SF-36 subscale scores for treatments. SF-36 subscale scores mean (and standard deviation) for groups<br>Start of Watsu / Completion of Watsu / Start of Aix / Completion of Aix<br>Physical function 47.308a (21.274) 63.847b (23.377) 56.154a (19.701) 60.000a (23.094)<br>Bodily pain 35.923a (19.350) 55. 077b (14.186) 42.692a (15.129) 42.308a (13.979)<br>Vitality 31.154a (16.975) 51.154b (19.912) 38.077a (20.365) 34.231a (19.879)<br>Social function 53.846a (21.881) 69.231b (22.018) 75.000a (17.678) 66.346a (23.599)<br>Role physical 41.539a (36.020) 61.692a (31.047) 47.692a (35.155) 41.539a (27.642)<br>General health 47.692a (23.859) 51.539a (15.191) 49.231a (19.023) 52.308a (19.538)<br>Role emotional 63.462a (33.253) 77.077a (27.723) 65.385a (37.553) 67.308a (41.313)<br>Mental health 63.692a (17.007) 74.000a (22.891) 69.231a (15.525) 71.385a (15.987)<br>Different subscripts (i.e., a then b) between means at start and completion of treatments indicate a significant difference<br>(P < .05) of SF-36 subscale score before and after treatment.
**Notes**	Temperature: 32−35 °C

Risk of bias table

Bias	Authors' judgement	Support for judgement
Random sequence generation (selection bias)	High risk	Reportedly no sequence generation. Participants were randomly assigned to receive either Watsu or Aix as their first treatment by placement in alternative treatments in the order that they were recruited.

Allocation concealment (selection bias)	Unclear risk	insufficient information
Analyzed within the group originally assigned to (selection bias)	Low risk	all participants underwent both conditions
Comparable baseline outcome measurements (selection bias)	High risk	carry-over effect described, baseline outcome measures provided only regarding treatment-effects (including carry-over), no absolute baseline-values of the two groups reported. --> only within-group measurements used in meta-analyses.
Comparable baseline characteristics (selection bias)	Unclear risk	n/a
Control for important confounding (selection bias)	Low risk	Counterbalancing was included to control order effects. Sequence effects were not considered problematic.<br>One had participated in a 3-week inpatient rehabilitation programme at QE Health, which included Aix massage, the other participants had no previous association with QE Health or the study treatments.
Blinding of participants and personnel (performance bias)	High risk	"To address possible confounding variables of varying peer group interaction, support, and use of QE Health FMS and related health information, the researcher introduced all the participants to each other, provided a room for informal group meetings, familiarized all participants with the QE Health library and arranged free access to the library resources for all participants for the duration of the study."
Impact from a concurrent intervention (performance bias)	Low risk	One had participated in a 3-week inpatient rehabilitation programme at QE Health, which included Aix massage,the other participants had no previous association with QE Health or the study treatments.
Fidelity to the intervention protocol maintained (performance bias)	Low risk	apparently no deviations
Missing data handled appropriately (attrition bias)	High risk	documented: 4 drop-outs in aix-massage before watsu treatment, not included in analysis. No intention to treat reported
Incomplete outcome data (attrition bias)<br>Objecitve Outcomes	Unclear risk	n/a
Incomplete outcome data (attrition bias)<br>Self-reported Outcomes	Low risk	apparently no missing (4 drop-outs in aix-massage befor watsu treatment, not included in entire analysis)
Length of follow-up (detection bias)	Low risk	Clearly defined: Each treatment block consisted of four sessions over 2 weeks, comprising of two sessions per week with a 2-day gap between sessions. The first treatment block was followed by 3 weeks of no treatment before commencement of the second treatment block

Blinding of outcome assessment (detection bias)	Unclear risk	insufficient information
Intervention clearly defined (detection bias)	Low risk	Clearly defined, 4 times, 32−35°C, 45min; Supported mainly by the forearms placed predominantly under the small of the back and/or the head, the therapist moves the participant through the water in flowing, rhythmical motions which includes intermittent gentle massage and stretching. No conversation takes place between the participant and therapist during therapy. The proximity between the participant and the therapist ranges from full arms length to close cradling.
Outcomes assessed reliably (detection bias)	Low risk	SF-36 validated in people with FMS
Confounding variables assessed (detection bias)	High risk	carry-over effect described, no further information
Potential outcomes prespecified (reporting bias)	Low risk	Clear declaration of outcomes
All prespecified outcomes reported (reporting bias)	Low risk	all outcomes prespecified in methods-section reported

Gimenes et al., 2006

**Methods**	CS
**Participants**	10 women with Fibromyalgia who did not participate in any previous treatment were included in the study. There was no age restriction, ranging from 40 to 82 years (± 53.37). Randomly referred by rheumatologists and orthopedists to the Clínica Escola do Centro Universitário São Camilo
**Interventions**	4 months treatment of Watsu: detailed enumeration of maneuvres. How many sessions???<br>(dance Breath, breath swing, concertina, accordion more cervical traction, rotating concertina, rotation of the leg inside, outside leg rotation, algae I and II, wobble arm- leg and sliding the column,open saddle and massage scapular. All movements (except the seals) were performed bilaterally.)
**Outcomes**	Two evaluations were performed, initial and final in relation to pain and depressive state, using visual analog scale (VAS)<br>Geriatric Depression- reduced version (GDS -15) (t-Test Sample Pared):<br>Initial Evaluation:(Mean ± SD)7.8 ±2.53 range: 5-10<br>FINAL Evaluation: (Mean ± SD)4.2 ±2.48 range: 1-8, p = 0.0055<br><br>Of the patients analyzed, initially 98.75% [sic!] complained of pain with intensity varying from level 2.5 to 10 (Chart 1), according to the VAS (± 5.82). After the Watsu treatment period, a significant reduction in pain intensity was evidenced. In the second evaluation, referred pain was between level 0 and 5.<br>VAS pain: presented on a table with patients per rating: [note: absolute numbers according to figure 1: pre: 2.5;5;4;10;6;8;0;8;9;5.7 --> mean 5.82) // post: 0;3;0;0;2;3;3;4;2;5 --> mean: 2.2]

**Notes**	Temperature: not mentioned, time of sessions not mentioned, amount of sessions not mentioned.

Risk of bias table

Bias	Authors' judgement	Support for judgement
Random sequence generation (selection bias)	Unclear risk	n/a
Allocation concealment (selection bias)	Unclear risk	n/a
Analyzed within the group originally assigned to (selection bias)	Unclear risk	n/a
Comparable baseline outcome measurements (selection bias)	Unclear risk	n/a
Comparable baseline characteristics (selection bias)	Unclear risk	n/a
Control for important confounding (selection bias)	Unclear risk	insufficient information, "As selection criteria, the patients were randomly referred by rheumatologists and orthopedists to the Clínica Escola do Centro Universitário São Camilo."
Blinding of participants and personnel (performance bias)	Low risk	yes: Both the screenings and the application of the protocol were performed by therapists blind to the study, duly trained to apply both the technique and the questionnaires.
Impact from a concurrent intervention (performance bias)	Unclear risk	insufficient information
Fidelity to the intervention protocol maintained (performance bias)	Unclear risk	insufficient information
Missing data handled appropriately (attrition bias)	Low risk	there was one missing data at baseline - no note, whether this was the only one. The missing data was calculated as zero, which in this case was to the disadvantage of effect.
Incomplete outcome data (attrition bias)<br>Objecitve Outcomes	Unclear risk	n/a
Incomplete outcome data (attrition bias)<br>Self-reported Outcomes	Low risk	apparently all patients reassessd in VAS and GDS-15
Length of follow-up (detection bias)	Unclear risk	insufficient information "4 months", no details about the procedure of data acquisition.
Blinding of outcome assessment (detection bias)	Unclear risk	insufficient information

Intervention clearly defined (detection bias)	High risk	how many sessions were delivered, duration of sessions, water temperature? Enough information about maneuvres is provided to assume that the article should be included.
Outcomes assessed reliably (detection bias)	Low risk	however, validity not reported
Confounding variables assessed (detection bias)	Unclear risk	insufficient information
Potential outcomes prespecified (reporting bias)	Low risk	prespecified outcomes stated
All prespecified outcomes reported (reporting bias)	High risk	all outcomes prespecified in methods-section reported, however: not clearly arranged, absolute values of the "fitnogram" missing.

Gimenez & Castro, 2018

**Methods**	CS
**Participants**	3 adolescents with cerebral palsy
**Interventions**	7 times WATSU in 8 weeks.
**Outcomes**	"...bringing benefits not only in sleep quality and spasticity as well as pain reduction."<br>Ashworth Scale: mean/SD pre 1.4/0.6 post 0.9/0.7<br>PSQI: mean/SD pre 7.3/5.5, post 9/10.8
**Notes**	33-35° C, 30min

Risk of bias table

**Bias**	**Authors' judgement**	**Support for judgement**
Random sequence generation (selection bias)	Unclear risk	n/a
Allocation concealment (selection bias)	Unclear risk	n/a
Analyzed within the group originally assigned to (selection bias)	Unclear risk	n/a
Comparable baseline outcome measurements (selection bias)	Unclear risk	n/a
Comparable baseline characteristics (selection bias)	Unclear risk	n/a
Control for important confounding (selection bias)	Low risk	criteria for in- and exclusion clearly stated
Blinding of participants and personnel (performance bias)	Unclear risk	insufficient information

Impact from a concurrent intervention (performance bias)	Unclear risk	insufficient information
Fidelity to the intervention protocol maintained (performance bias)	Low risk	Documented: Problems with pool heater reported, thus less WATSU-sessions provided (planned: 9 sessions)
Missing data handled appropriately (attrition bias)	Low risk	Full report on all participants, including drop-out (ITT)
Incomplete outcome data (attrition bias)<br>Objecitve Outcomes	Low risk	apparently no missing data
Incomplete outcome data (attrition bias)<br>Self-reported Outcomes	Low risk	apparently no missing data
Length of follow-up (detection bias)	Low risk	reasessment in last session
Blinding of outcome assessment (detection bias)	Unclear risk	insufficient information
Intervention clearly defined (detection bias)	Low risk	Detailed description provided: "opening sequence, breath swing, free movement, accordion, rotary accordion and inward and outward rotation."
Outcomes assessed reliably (detection bias)	Low risk	Modified Ashworth Scale,<br>Pittsburgh Sleep Quality Index (PSQI)<br>sleep only qualitatively reported
Confounding variables assessed (detection bias)	Unclear risk	insufficient information
Potential outcomes prespecified (reporting bias)	Low risk	prespecified outcomes stated
All prespecified outcomes reported (reporting bias)	Low risk	all outcomes prespecified in methods-section reported

Hora et al., 2017

**Methods**	CS: mixed methods, quase-experimental, trial without control group
**Participants**	36 healthy students from the Centro Universitário Tiradentes (UNIT-AL)
**Interventions**	WATSU, 40-45min, WATSU-positions reported.
**Outcomes**	Quantitative: There was no SBP (systolic blood pressure) difference between “before” (102,35±10,46) and «after» (98,53±15,98) Watsu. The difference between SBP and DBP was observed between “repose 1” (before entering the pool) (SBP 116,47±10,41, DBP 76,47±8,48) and «after» (before leaving the pool) (SBP 98,53±15,98, DBP 62,65±13,09). There was no change in the HR «before» (73,65±12,15) and «after» (71,32±12,71); but the absolute values were lower than «repose 1» (86,24±13,49).<br>Conclusion: The variations observed in the physiological variables seem to be more related to the water temperature than directly to the Watsu session.<br>Qualitative: "How long do you believe the session lasted? Reality: 40 ± 5 min, perceived 29.4 ± 1.9 min.<br>Talk about the sensations, situations, feelings and thoughts experienced during

	the Watsu session: relaxation (61%), peace (30%), tranquility (25%), lightness (24%), wellbeing (11%).<br>Was there any movement, position or support that you liked the most? seaweed (33%)<br>How do you feel at that moment (after the session)? 72.22% of participants reported feeling of relaxation; 19.44% lightness; and 16.67% of tranquility.<br>Are you interested in participating in a 10-session Watsu program? 100% yes<br>The analysis of the subjective reports obtained with the application of the adapted questionnaire was performed after organization in categories and demonstrated as a percentage, with the exception of the first question that referred to the perceived time of intervention."<br>Conclusion: Watsu, applied only once, seems to influence the sensation of body relaxation.
**Notes**	34 °C "There was no report of malaise or negative feelings about the session."

Risk of bias table

Bias	Authors' judgement	Support for judgement
Random sequence generation (selection bias)	Unclear risk	n/a
Allocation concealment (selection bias)	Unclear risk	n/a
Analyzed within the group originally assigned to (selection bias)	Unclear risk	n/a
Comparable baseline outcome measurements (selection bias)	Unclear risk	n/a
Comparable baseline characteristics (selection bias)	Unclear risk	n/a
Control for important confounding (selection bias)	Low risk	in- and exclusion criteria clearly stated
Blinding of participants and personnel (performance bias)	Unclear risk	insufficient information
Impact from a concurrent intervention (performance bias)	Low risk	not likely, healthy participants
Fidelity to the intervention protocol maintained (performance bias)	Low risk	Deviation reported (measurement of pain was limited due to 86% of the respondents not declaring any pain of musculoskeletal origin).
Missing data handled appropriately (attrition bias)	Low risk	insufficient information, but attrition is not likely an issue in a single session pre-post-design
Incomplete outcome data (attrition bias)<br>Objecitve Outcomes	Low risk	insufficient information, but attrition is not likely an issue in a single session pre-post-design

Incomplete outcome data (attrition bias)<br>Self-reported Outcomes	Low risk	insufficient information, but attrition is not likely an issue in a single session pre-post-design
Length of follow-up (detection bias)	Low risk	standardized conditions: "Upon arrival for the session, the participants remained for 5 minutes at rest and after that time the Vital signs of Blood Pressure (BP) and Heart Rate (HR) were checked and recorded. Then the participant was directed to enter the pool. As soon as the individual immersed, the vital signs (PA and FC) were again verified and after that the same was instructed to stay for 5 minutes immersed in the height of the xiphoid process for later gauging of the signs already mentioned above. After these procedures the Watsu session was started.<br>After the end of the session, the patient's BP and HR were checked, and the patient was instructed to leave the pool and remain at rest for five minutes for the last measurement."
Blinding of outcome assessment (detection bias)	Unclear risk	insufficient information
Intervention clearly defined (detection bias)	Low risk	"Each session had duration of 40 ± 5 minutes, and it was possible to perform with two individuals concomitantly. The movements chosen to perform the technique were: dance of the breath, swing of the breath, offering in spiral, lullaby, accordion associated with the traction of the column, rotating concertina, rotation with the inside leg, rotation with the outside leg, algae, massage along the back of the individual, curling the spine and waiter (WABA BRASIL, 2003)."
Outcomes assessed reliably (detection bias)	Low risk	"Blood Pressure (BP) and Heart Rate (HR). For verification of the physiological parameters, a Premium Aneroid Sphygmomanometer and Littimann Classic II Stethoscope were used for BP measurement, and an Onyx 9500-Nonin Pulse Oximeter was used to measure HR."
Confounding variables assessed (detection bias)	Unclear risk	insufficient information
Potential outcomes prespecified (reporting bias)	Low risk	prespecified outcomes stated
All prespecified outcomes reported (reporting bias)	High risk	VAS on pain was not reported: "However, in the first moment ("respouso1"), it was observed that the majority (86.11%) did not present any report of a painful complaint of musculoskeletal origin. This fact limited the continuity in evaluating this variable in the different moments of analysis of the other study variables."

Israel et al., 2006

**Methods**	CS
**Participants**	Three male patients with clinical diagnosis of ankylosing spondylitis, 25 to 60 yers of age
**Interventions**	15 WATSU sessions of 30 min
**Outcomes**	pain decreased in all patients (measured by Numeric Rating Scale)
**Notes**	34 °C

Risk of bias table

Bias	Authors' judgement	Support for judgement
Random sequence generation (selection bias)	Unclear risk	n/a
Allocation concealment (selection bias)	Unclear risk	n/a
Analyzed within the group originally assigned to (selection bias)	Unclear risk	n/a
Comparable baseline outcome measurements (selection bias)	Unclear risk	n/a
Comparable baseline characteristics (selection bias)	Unclear risk	n/a
Control for important confounding (selection bias)	Unclear risk	insufficient information
Blinding of participants and personnel (performance bias)	Unclear risk	insufficient information
Impact from a concurrent intervention (performance bias)	Unclear risk	insufficient information
Fidelity to the intervention protocol maintained (performance bias)	Low risk	apparently no deviations from study protocol
Missing data handled appropriately (attrition bias)	Low risk	apparently no missing data
Incomplete outcome data (attrition bias) Objecitve Outcomes	Unclear risk	n/a
Incomplete outcome data (attrition bias) Self-reported Outcomes	Low risk	apparently no missing values
Length of follow-up (detection bias)	Low risk	pre-post
Blinding of outcome assessment (detection bias)	Unclear risk	insufficient information
Intervention clearly defined (detection bias)	High risk	time-frame of treatment-series not reported

Outcomes assessed reliably (detection bias)	Low risk	NRS pain
Confounding variables assessed (detection bias)	Unclear risk	insufficient information
Potential outcomes prespecified (reporting bias)	Unclear risk	insufficient information
All prespecified outcomes reported (reporting bias)	Unclear risk	insufficient information: "Among the main results were pain reduction in the NRS"

Jithin & Adarsh, 2019

**Methods**	RCT
**Participants**	20 school level swimmers (age 10-14 years)
**Interventions**	"The watsu water relaxation training group underwent 7 weeks of relaxation training program on 3 days per week."<br>Control group not declared.
**Outcomes**	heart rate, blood pressure and body temperature. "The result of the study indicated that there was a significant acute and also long term effect of watsu water relaxation training on the selected physiological variables."
**Notes**	no ethic statement. Temperature only in description of WATSU, not in specific study. Duration of sessions not specified.

Risk of bias table

**Bias**	**Authors' judgement**	**Support for judgement**
Random sequence generation (selection bias)	Unclear risk	insufficient information, "randomly assigned"
Allocation concealment (selection bias)	Unclear risk	insufficient information
Analyzed within the group originally assigned to (selection bias)	Unclear risk	insufficient information, no flow-chart provided
Comparable baseline outcome measurements (selection bias)	Low risk	Data reported in Tables 1, 3 and 5
Comparable baseline characteristics (selection bias)	Unclear risk	insufficient information
Control for important confounding (selection bias)	Unclear risk	insufficient information
Blinding of participants and personnel (performance bias)	Unclear risk	insufficient information
Impact from a concurrent intervention (performance bias)	Unclear risk	insufficient information.

Fidelity to the intervention protocol maintained (performance bias)	Low risk	apparently no deviations from study protocol
Missing data handled appropriately (attrition bias)	Unclear risk	insufficient information
Incomplete outcome data (attrition bias)<br>Objecitve Outcomes	Low risk	apparently no missing data
Incomplete outcome data (attrition bias)<br>Self-reported Outcomes	Unclear risk	n/a
Length of follow-up (detection bias)	Low risk	"tested just before the training and quickly after the training for both groups" just before the 7 weeks or at every treatment? However, the same for both groups
Blinding of outcome assessment (detection bias)	Unclear risk	insufficient information
Intervention clearly defined (detection bias)	High risk	"WATSU", no further details. It is not clear, what the control group did. Values are obviously lower in both groups.
Outcomes assessed reliably (detection bias)	High risk	"Sphygmomanometer and stethoscope is used for blood pressure, stopwatch and manual counting is used for heart rate assessment, and thermometer is used for measuring body temperature". Manual counting invites errors. Values are suspiciously high at baseline in both groups (Heart rate mean 165.7 SD 10.08 in experimental versus 170.4/9.39 in control group. Was this at rest??)
Confounding variables assessed (detection bias)	Unclear risk	insufficient information
Potential outcomes prespecified (reporting bias)	Low risk	prespecified outcomes stated
All prespecified outcomes reported (reporting bias)	Low risk	all outcomes prespecified in methods-section reported

Lima et al., 2009

**Methods**	CR
**Participants**	1 woman, 21 years, moderate Asthma
**Interventions**	10 sessions of Watsu (twice weekly for 60 min. during 5 weeks)
**Outcomes**	FVC, FEV1, FEV1 / FVC, PEF, Kakizaki Method<br>Quality of Life in Asthma (QQV)<br>Beck Depression Inventory (BDI)<br>and chest cirtometry<br>Spielberger (STAI-State-Trait-Anxiety-Inventory) in the first, sixth and tenth session in order to assess immediate anxiety before and after treatment.<br>Each session: SO2, heart rate, respiratory rate and peripheral oxygen saturation were measured for pre and post-intervention control.<br>At the end of the 10 treatment sessions, a new Pulmonary Function Test was

	performed and the QQV, STAI, BDI and thoracic circometry questionnaires were again applied.<br>Results:<br>In the values of thoracic cirtometry, pre and posttreatment, there was an increase in the axillary measurement: first expiration 2cm, inspiration 3cm and 2nd expiration 5cm. [note: the last measurement is NOT in accordance with the graph in the paper, which shows approximately 3 cm!!]<br>In the xiphoid measurement: first expiration 7cm, inspiration 5.5cm and 2nd expiration 6.5cm.<br>In umbilical measurement: only 0.5 cm at the 2nd expiration (Figure 1).<br><br>In spirometric values, pre and posttreatment, a 300ml increase in FEV1 (12%) can be observed; An increase of 8.29L in FEV1 / FVC (11%); Increase of 102.1 L / min in PEF (39%). FVC showed a small decrease (-0.3%) (Table 1).<br><br>In the Inventário Ansiedade IDATE-ESTADO AIE (State-Anxiety-Inventory STAI), there was a 9 point decrease in the first session. At the sixth session, there was a drop of 14 points and in the tenth session, a decrease of 11 points. In all three results, the patient changed the anxiety state score to non-anxiety at the end of the session.<br>In the pre and post-treatment values (day 1 and day 45, respectively) of the Ansiedade IDATE-TRAÇO AIT (Trait-Anxiety), there was an increase of 13 points, and in the BDI [which version??], an increase of only 2 points.<br>For the Qualidade de Vida na Asma QQV (QoL), in the same period, it can be observed in the domains presented: a reduction of 6.5% in the limitation of physical activities due to the symptoms of asthma, a 33.4% decrease in the frequency and severity of symptoms, an increase of 16, 7% in adherence to treatment, an increase of 8.3% in the socioeconomic domain and a decrease of 7.1% in the psycho-social domain.<br><br>Measurements of heart rate, respiratory rate and saturation, before and after session, did not present clinical alterations.<br>Considering the results obtained in the present study, the application of the Watsu Method suggests to be beneficial in relation to the thoracic mobility, ventilation, state of anxiety and quality of life of the asthmatic individual.
**Notes**	Temperature: ±34 °C, all provided values recalculated

Risk of bias table

**Bias**	**Authors' judgement**	**Support for judgement**
Random sequence generation (selection bias)	Unclear risk	n/a
Allocation concealment (selection bias)	Unclear risk	n/a
Analyzed within the group originally assigned to (selection bias)	Unclear risk	n/a

Comparable baseline outcome measurements (selection bias)	Unclear risk	n/a
Comparable baseline characteristics (selection bias)	Unclear risk	n/a
Control for important confounding (selection bias)	Unclear risk	insufficient information
Blinding of participants and personnel (performance bias)	Unclear risk	n/a
Impact from a concurrent intervention (performance bias)	Unclear risk	insufficient information
Fidelity to the intervention protocol maintained (performance bias)	Low risk	apparently no deviations from protocol
Missing data handled appropriately (attrition bias)	Unclear risk	n/a
Incomplete outcome data (attrition bias)<br>Objecitve Outcomes	Low risk	apparently no missing data
Incomplete outcome data (attrition bias)<br>Self-reported Outcomes	Low risk	apparently no missing data
Length of follow-up (detection bias)	Unclear risk	n/a
Blinding of outcome assessment (detection bias)	Unclear risk	insufficient information
Intervention clearly defined (detection bias)	Low risk	yes ±34°C, 10 sessions, 60min, twice weekly
Outcomes assessed reliably (detection bias)	Low risk	yes, however: we do not know, which version of BDI was employed.
Confounding variables assessed (detection bias)	Unclear risk	insufficient information
Potential outcomes prespecified (reporting bias)	Low risk	prespecified outcomes stated
All prespecified outcomes reported (reporting bias)	High risk	no absolute values reported concerning the questionnaires, "Measurements of heart rate, respiratory rate and saturation, before and after session, did not present clinical alterations."

Mota et al., 2007

**Methods**	CS
**Participants**	28 healthy students of Vale do Paraíba University, UNIVAP, São José dos Campos – SP, male and female
**Interventions**	2 Sessions of WATSU, 40min, pre-post-measuring

**Outcomes**	activity of the sympathetic and parasympathetic nervous system based on the analysis of heart rate variability HRV (BioExpress Heart Rhythm Instruments, Incorporation, the USA)<br>An imbalance of the autonomic nervous system was observed, with activation of the sympathetic nervous system in 100% of the evaluated ones. In the physical aptitude test all the subjects obtained low levels of functioning of the physiological systems and reserve of adaptation.<br><br>Most of the patients analyzed in this study were classified into categories that indicate pathological conditions and / or imbalance of the sympathetic nervous system, except one, which was classified in category 3, which also corresponds to a category of sympathetic activation.<br><br>With regard to the myocardial chronotropic reaction, it was observed that sixteen individuals (57%) were in categories 4 and 5, which represent mild and moderately reduced chronotropic reactions, respectively, progressing to categories 3 and 4 corresponding respectively to the near normality And slightly reduced. Category 4, which indicates a reduction in parasympathetic nervous system activity associated with an increase in the sympathetic nervous system, concentrated 21 subjects (75%) before the intervention, representing most of them.<br>There was an 8% increase in the level of activity of the sympathetic nervous system and a 68% increase in the values of the parasympathetic level after the Watsu Therapy. When we performed the paired t-student test for the SNPS values before and after intervention with Watsu therapy we observed a statistically significant reduction with a value of $p < 0.02$ for a significance level of 0.05 in a confidence interval of 95%:<br>SNPS TOTAL / SNS TOTAL<br>Pré -0,89 / 1,42<br>Pós -0,28 / 1,53<br>p = 0,02 / ns<br><br>57% of the subjects analyzed in this study showed an improvement in the level of functioning of the physiological systems, and only five subjects had the evaluation of the physiological systems above the average level before Watsu therapy. After the intervention, we observed that nine individuals started to present the above average levels for the functioning of the physiological systems.<br><br>All subjects analyzed in this study were classified by Software Health-Express in the region of the phytogram indicating the levels of functioning of the physiological and reserve systems of adaptation, according to figure 2.<br>Figure 2 - Phytogram of physiological systems functioning levels and Health-Express adaptation reserve, obtained in a test performed with a subject involved in this study.<br><br>A statistically significant increase ($p < 0.007$) was observed when performing the paired t-test for the functioning levels of the physiological and reserve systems of adaptation before and after Watsu Therapy.
**Notes**	33-34 °C

Risk of bias table

Bias	Authors' judgement	Support for judgement
Random sequence generation (selection bias)	Unclear risk	n/a
Allocation concealment (selection bias)	Unclear risk	n/a
Analyzed within the group originally assigned to (selection bias)	Unclear risk	n/a
Comparable baseline outcome measurements (selection bias)	Unclear risk	n/a
Comparable baseline characteristics (selection bias)	Unclear risk	n/a
Control for important confounding (selection bias)	Low risk	clear in- and exclusion criteria: "musculoskeletal, neurological, cardiorespiratory or vestibular alterations were adopted as exclusion criteria. In addition, individuals with chlorine allergy or those who did not consent to participate in the study were also excluded."
Blinding of participants and personnel (performance bias)	Unclear risk	insufficient information
Impact from a concurrent intervention (performance bias)	Unclear risk	n/a
Fidelity to the intervention protocol maintained (performance bias)	Low risk	apparently no deviations
Missing data handled appropriately (attrition bias)	Unclear risk	insufficient information
Incomplete outcome data (attrition bias)<br>Objecitve Outcomes	Unclear risk	insufficient information
Incomplete outcome data (attrition bias)<br>Self-reported Outcomes	Unclear risk	n/a
Length of follow-up (detection bias)	Low risk	standardized procedures described
Blinding of outcome assessment (detection bias)	Unclear risk	insufficient information
Intervention clearly defined (detection bias)	Low risk	yes "Subjects were submitted to two 40-minute sessions of Watsu therapy, the first to adapt to the technique"
Outcomes assessed reliably (detection bias)	High risk	No validity of NerveExpress reported
Confounding variables assessed (detection bias)	Unclear risk	insufficient information

Potential outcomes prespecified (reporting bias)	Low risk	prespecified outcomes declared
All prespecified outcomes reported (reporting bias)	High risk	all outcomes mentioned in the methods section reported, however: not clearly arranged, absolute values of the "fitnogram" missing.

Nakamoto, 2016

**Methods**	CR
**Participants**	f, 63 yrs, history of falls
**Interventions**	10 times WATSU, twice a week. Detailed description provided
**Outcomes**	There was an increase of 15 points, changing the volunteer's score from predisposition to fall risk to no fall risk according to the Berg-Balance-Scale assessment (from 39 to 54).
**Notes**	33-35° C, 60 min

Risk of bias table

**Bias**	**Authors' judgement**	**Support for judgement**
Random sequence generation (selection bias)	Unclear risk	n/a
Allocation concealment (selection bias)	Unclear risk	n/a
Analyzed within the group originally assigned to (selection bias)	Unclear risk	n/a
Comparable baseline outcome measurements (selection bias)	Unclear risk	n/a
Comparable baseline characteristics (selection bias)	Unclear risk	n/a
Control for important confounding (selection bias)	Unclear risk	insufficient information
Blinding of participants and personnel (performance bias)	Unclear risk	n/a
Impact from a concurrent intervention (performance bias)	Unclear risk	insufficient information
Fidelity to the intervention protocol maintained (performance bias)	Low risk	apparently no deviations from protocol
Missing data handled appropriately (attrition bias)	Unclear risk	n/a
Incomplete outcome data (attrition bias)<br>Objecitve Outcomes	Low risk	apparently no missing data

Incomplete outcome data (attrition bias)<br>Self-reported Outcomes	Unclear risk	n/a
Length of follow-up (detection bias)	Unclear risk	n/a
Blinding of outcome assessment (detection bias)	Unclear risk	insufficient information
Intervention clearly defined (detection bias)	Low risk	Detailed description provided: dance/balancing breathing, slow movements, accordion, releasing the back, exploring movements, explore door I, explore door II, quiet, seaweed position: with the right shoulder, turn and pull, sweep under shoulder, spine pull , curling the back, side saddle, open saddle or closed saddle, head move lift wave, side sandwich A, side sandwich B, final position on the wall.
Outcomes assessed reliably (detection bias)	Low risk	Berg Balance Scale
Confounding variables assessed (detection bias)	Unclear risk	insufficient information
Potential outcomes prespecified (reporting bias)	Low risk	prespecified outcomes declared
All prespecified outcomes reported (reporting bias)	Low risk	all outcomes prespecified in methods-section reported

Pastrello et al., 2009

**Methods**	CR
**Participants**	one 4 year 4 month old first twin, male, diagnosed with spastic tetraplegia cerebral palsy (CID G 80.0) and Gross Motor Function Classification System level V. first twin.
**Interventions**	Stage I: 2x land/week for 8 weeks<br>Stage II: 2x WATSU, 30min, plus 1x land/week for 8 weeks
**Outcomes**	GMFM (Gross Motor Function Measure), dimensions A and B (lie, roll, sit; physical function, mobility). Assessment took place three times: the first assessment in the beginning of the study; the second assessment after the intervention on the floor; and the third assessment after Watsu therapy was applied associated to the floor intervention.<br>In both Dimensions, changes in performance were not observed during Stage I. During the third assessment, a significant increase was observed in comparison to the performances on assessments I and II as Dimension A [according to figura 1 approximately from 70 to 85], and a notsignificant increase in B [35 to 38].<br>Additional information from Conference-proceedings:<br>In the first evaluation, as presented in Table 1, the child presented in Dimension A a total of hits equal to 36 points out of 51 possible, resulting in 70% of achievement. In dimension B, a total of 21 successful points of 60 possible, totaling a 35% utilization (Table 2). The same values were found in the second evaluation for both A and B.<br>In the last evaluation the child presented a score of 45 points out of a total of 51

	possible points, resulting in a score relative to 88% of achievement in dimension A, and in dimension B, presented a score of 23 points out of a total of 60 possible, resulting In a score relative to 38% of achievement, according to Tables 1 and 2, respectively.<br>When comparing the performances of the beginning and the end of the treatment, involving both Dimension A and Dimension B, there is an evolution in the child's performance of 52.5%, keeping 52.5% in the second evaluation, and to 63% at the end.
**Notes**	"Watsu therapy was capable of assisting in motor rehabilitation of a child with spastic tetraplegia cerebral palsy."<br>Temperature: 33 °C

Risk of bias table

Bias	Authors' judgement	Support for judgement
Random sequence generation (selection bias)	Unclear risk	n/a
Allocation concealment (selection bias)	Unclear risk	n/a
Analyzed within the group originally assigned to (selection bias)	Unclear risk	n/a
Comparable baseline outcome measurements (selection bias)	Unclear risk	n/a
Comparable baseline characteristics (selection bias)	Unclear risk	n/a
Control for important confounding (selection bias)	Low risk	"The inclusion criteria were not to have cognitive / visual impairment and to accept the liquid medium; As exclusion, to have previously received the Watsu method, to be inserted in another program of physiotherapeutic intervention and to change the dose of the muscle relaxant during the study."
Blinding of participants and personnel (performance bias)	Unclear risk	n/a
Impact from a concurrent intervention (performance bias)	Low risk	Multimodal approach (aquatic therapy plus floor physical therapy in stage II). In the first Stage, the amount of land-based therapy was twice as high and did not lead to results at all. The child as though by chance could have experienced a developmental milestone just at the time of Stage II.
Fidelity to the intervention protocol maintained (performance bias)	Low risk	apparently no deviations
Missing data handled appropriately (attrition bias)	Unclear risk	n/a

Incomplete outcome data (attrition bias)<br>Objecitve Outcomes	Low risk	apparently no missing data
Incomplete outcome data (attrition bias)<br>Self-reported Outcomes	Unclear risk	n/a
Length of follow-up (detection bias)	Unclear risk	n/a
Blinding of outcome assessment (detection bias)	Unclear risk	insufficient information: "We also measured the gross motor function of Dimensions A (lying down and rolling) and B (sitting) with the GMFM scale before Stage I, after Stage I and after Stage II, always performed with the same criteria and by the same evaluator." blinded?
Intervention clearly defined (detection bias)	Low risk	session 30 min, temperature: 33°
Outcomes assessed reliably (detection bias)	Low risk	GMFM (Gross Motor Function Measure), dimensions A and B, performed with the same criteria and by the same evaluator
Confounding variables assessed (detection bias)	Unclear risk	insufficient information
Potential outcomes prespecified (reporting bias)	Low risk	prespecified outcomes declared
All prespecified outcomes reported (reporting bias)	Low risk	all outcomes prespecified in methods-section reported

Pinkalsky et al., 2011

**Methods**	CS
**Participants**	5 patients with fibromyalgia, female, age 50 to 68
**Interventions**	5 individual Watsu sessions, 60 minutes, once weekly
**Outcomes**	BEGINNING and END of treatment series:<br>FIQ (physical function, overall impact and symptoms) pre (7.21 ± 1.21). After the treatment FIQ (4.54 ± 1.67) [roughly: pre: min: 5.2, max: 8.2, 25%: 6, median: 7.6, 75%: 8.1; post: min: 2.6, max: 6.1, 25%: 2.8, median: 5.1, 75%:6], not significant<br>and<br>WHOQOL-brief (Physical health, mental health, personal social relationships, environment) to assess quality of life. improvement in all four WHOQOL-bref domains, significant<br>Physical aspect p = 0.0033 [roughly: pre: min: 10, max 15, 25%: 11, median: 12, 75%:14; post: min: 17, max: 25, 25%:17.5, median: 19, 75%: 22]<br>personal relationships p = 0.0123 [roughly: pre: min: 4, max: 12, 25%: 5.5, median: 9, 75% 11.5; post: min: 8, max: 13, 25%: 8]<br>before and after EACH session:<br>VAS pain<br>"After the five sessions of Watsu, all patients reported Significant decrease in pain (p = 0.0043)." "Before 3 to 8 (± 5.40) (mean?)" [according to Graph 1: 5.4

	mean, SD ± 2.1].
**Notes**	Temperature: 34 °C

Risk of bias table

Bias	Authors' judgement	Support for judgement
Random sequence generation (selection bias)	Unclear risk	n/a
Allocation concealment (selection bias)	Unclear risk	n/a
Analyzed within the group originally assigned to (selection bias)	Unclear risk	n/a
Comparable baseline outcome measurements (selection bias)	Unclear risk	n/a
Comparable baseline characteristics (selection bias)	Unclear risk	n/a
Control for important confounding (selection bias)	Low risk	clear in- and exclusion criteria: "The inclusion criteria were: Patients of both sexes, who presented the diagnosis of fibromialgia, medical referral and dermatological attestation To enter the swimming pool of the institution. The criteria Exclusion criteria were: patients who lacked more than once without justification, had some contraindication to the practice of hydrotherapy, presented some cognitive deficit or neurological, or were practicing another activity In therapeutic pool."<br>The sessions were individual, applied by the same therapist.
Blinding of participants and personnel (performance bias)	Unclear risk	insufficient information
Impact from a concurrent intervention (performance bias)	Unclear risk	insufficient information, no other hydrotherapy allowed
Fidelity to the intervention protocol maintained (performance bias)	Low risk	clear treatment protocol stated (treatment procedure)
Missing data handled appropriately (attrition bias)	Unclear risk	insufficient information
Incomplete outcome data (attrition bias)<br>Objecitve Outcomes	Unclear risk	n/a
Incomplete outcome data (attrition bias)<br>Self-reported Outcomes	Low risk	no drop-outs "zero absences, even under unfavorable climatic conditions."
Length of follow-up (detection bias)	Unclear risk	insufficient information

Blinding of outcome assessment (detection bias)	Unclear risk	insufficient information
Intervention clearly defined (detection bias)	Low risk	60 min, temperature 34°C, detailed description
Outcomes assessed reliably (detection bias)	Low risk	FIQ<br>WHOQOL-short<br>VAS pain
Confounding variables assessed (detection bias)	Unclear risk	insufficient information
Potential outcomes prespecified (reporting bias)	Low risk	prespecified outcomes declared
All prespecified outcomes reported (reporting bias)	Low risk	all outcomes prespecified in methods-section reported

Rambo & Filippin, 2019

**Methods**	CS
**Participants**	15 premature newborns
**Interventions**	10 min of WATSU, pre and post measurements
**Outcomes**	Table 2 - Respiratory rate per minute (RR), heart rate per minute(HR), peripheral oxygen saturation (Spo2), mean arterial pressure (MAP), pain and sleep at the four assessment times (n = 15) Measurements 1 and 2 are pre and post painful procedure on day 1; 3 and 4 are post painful procedure = pre WATSU and post WATSU on day 2<br>Variables N Mean SD Median Min Max Teste estatístico p<br>RR1 15 49,6 14,75 44 33 73 Friedman 0,002*<br>RR2 15 57,6 14,69 57 30 85<br>RR3 15 48,33 11,35 43 36 70<br>RR4 15 49,47 10,38 53 34 65<br>HR1 15 149,67 13,42 145 129 173 Friedman <0,001*<br>HR2 15 162,8 16,58 161 143 201<br>HR3 15 157,4 16,08 155 140 192<br>HR4 15 148,8 16,34 145 132 196<br>SpO2 1 15 96,53 2,33 97 92 100 Friedman <0,001*<br>SpO2 2 15 92 4,77 94 83 98<br>SpO2 3 15 95,81 1,82 96 93 98<br>SpO2 4 15 96,47 2,75 97 88 100<br>MAP1 15 62,93 16,54 59 39 89 Friedman 0,481<br>MAP2 15 65,13 16,14 61 41 87<br>MAP3 15 64,26 13,56 66 35 86<br>MAP4 15 62,07 12,09 66 38 81<br>Sleep1 15 1,4 0,63 1 1 3 Friedman <0,001*<br>Sleep2 15 4,07 1,79 4 1 6<br>Sleep3 15 1,4 0,91 1 1 4<br>Sleep4 15 3,2 0,77 3 2 5<br>Pain1 15 4,4 1,51 4 3 7 Friedman <0,001*<br>Pain2 15 6,13 2,64 5 2 12

	Pain3 15 4 1,07 4 3 6<br>Pain4 15 3,73 1,79 4 2 9
**Notes**	37° C, 10 min

Risk of bias table

Bias	Authors' judgement	Support for judgement
Random sequence generation (selection bias)	Unclear risk	n/a
Allocation concealment (selection bias)	Unclear risk	n/a
Analyzed within the group originally assigned to (selection bias)	Unclear risk	n/a
Comparable baseline outcome measurements (selection bias)	Unclear risk	n/a
Comparable baseline characteristics (selection bias)	Unclear risk	n/a
Control for important confounding (selection bias)	Low risk	criteria for in- and exclusion clearly stated
Blinding of participants and personnel (performance bias)	Unclear risk	insufficient information
Impact from a concurrent intervention (performance bias)	Unclear risk	insufficient information
Fidelity to the intervention protocol maintained (performance bias)	Unclear risk	insufficient information
Missing data handled appropriately (attrition bias)	Unclear risk	insufficient information
Incomplete outcome data (attrition bias)<br>Objecitve Outcomes	Unclear risk	insufficient information
Incomplete outcome data (attrition bias)<br>Self-reported Outcomes	Unclear risk	n/a
Length of follow-up (detection bias)	High risk	Time points of measurements are not identical:<br>1st day<br>1st assessment of vital signs: HR, RF, SpO2, MAP, sleep and pain.<br>2nd Painful procedure<br>3rd Reassessment: HR, RF, SpO2, sleep and pain.<br><br>2nd day<br>1st painful procedure<br>2nd Evaluation of vital signs: HR, RF, SpO2, MAP, sleep

		and pain.<br>3rd Aquatic Physiotherapy<br>4th Reassessment: FC, FR, SpO2, MAP, sleep and pain.<br>While data of collection 2 and 3 are both after the painful procedure (day 1 and day 2), they differ considerable. No explaination is provided by the authors.
Blinding of outcome assessment (detection bias)	Unclear risk	insufficient information
Intervention clearly defined (detection bias)	Low risk	The intervention lasted 10 minutes and was performed in a bathtub with warm water around 37º monitored with a thermometer, the NB was immersed to the shoulders, with a cap to avoid excessive heat loss. Rhythmic and slow movements were performed, associated with lower and upper limb girdle and elongation mobilizations based on the Watsu hydrotherapy method.16 In some moments, the NB was positioned in a flexor pattern to provide relaxation and safety. Therapy with the NB was completed in the incubator or warm crib positioned supine and flexed limbs close to the body.
Outcomes assessed reliably (detection bias)	Low risk	Evaluation form included information on the study variables, HR, respiratory rate (RR), mean arterial pressure (MAP), peripheral O2 saturation (SpO2), pain and sleep. To check vital signs (HR, MAP and SpO2), a Dash 4000 multiparameter GE monitor was used. To verify RF, abdominal movement was observed for 1 minute. Pain was assessed using the Premature Infant Pain Profile (PIPP) scale, which consists of a multidimensional measure of seven pain items, which has been widely used to assess acute pain in premature infants14. Finally, sleep was assessed using the Brazelton adapted sleep-wake rating scale, coded from 1 to 6: 1 = deep sleep, 2 = light sleep, 3 = sleepiness, 4 = inactive alertness, 5 = alert with activity, 6 = crying, with only one possible alternative for each assessment. All evaluation was performed in the morning between 7h and 7h 15min.insuf
Confounding variables assessed (detection bias)	Unclear risk	insufficient information
Potential outcomes prespecified (reporting bias)	Low risk	prespecified outcomes declared
All prespecified outcomes reported (reporting bias)	Low risk	all outcomes prespecified in methods-section reported

Ramirez et al., 2019

**Methods**	RCT
**Participants**	30 patients of juvenile idiopathic arthritis from the Teletón Santiago Institute, 8 to 18 years of age (3.17 ± 3.02 years for the watsu group and 12.68 ± 3.00 years for the conventional hydrotherapy group, p = 0.880)
**Interventions**	This study considered two parallel groups, previously randomized, where the experimental group: received a Watsu therapy protocol and the<br>control group: received a protocol of conventional hydrotherapy.<br>Both protocols lasted 10 sessions, once a week, with a duration of 45 minutes per session.
**Outcomes**	"With regard to health-related quality of life, measured through PedsQL4.0, watsu therapy presented a statistically significant difference in the physical functioning sub-dimension at the baseline level (p = 0.028) and a statistically significant improvement tiva post-intervention (p = 0.041) compared to conventional hydrotherapy. In the intra-group evaluation, watsu therapy showed significant differences in the improvement in the sub-dimension of psychosocial health between the baseline assessment and follow-up (p = 0.021). Conventional hydrotherapy did not show significant intra-group differences in the sub-dimensions or in the overall score of quality of life (table 3)."<br>"Functional health status and pain sensation In the application of the Childhood Health Assessment Questionnaire (CHAQ), the Watsu therapy obtained statistically significant improvements in the post-intervention evaluation of the disability index (discapilidad) (p = 0.015), index of discomfort (malestar) (p = 0.031), index of state of health (estado de salud) (p = 0.013) and total score CHAQ (p 0 = 0.003) in comparison to conventional hydrotherapy. In the intra-group evaluation of each CHAQ index, no statistically significant differences were observed in the disability index in the watsu or hydrotherapy group (Table 4). In the index of malaise, watsu therapy obtained a statistically significant improvement in the post-intervention evaluation (p = 0.007), on the contrary, in the<br><br>index of health status, watsu therapy obtained a statistical deterioration. Significantly between the post-intervention and follow-up evaluation (p = 0.012). On the other hand, the health status index of conventional hydrotherapy showed statistically significant improvements between post- intervention evaluation and follow-up (p = 0.033) and deterioration between baseline and follow-up (p = 0.017). Finally, in the total value of CHAQ, watsu therapy showed a statistically significant improvement in the functional health status between the baseline and post-intervention evaluations (p = 0.004) and a statistically significant deterioration of it. variable between post-intervention and follow-up evaluations (p = 0.039) (Table 4)."<br>"Articular ranges of movement In the 10-joints Global Range of Motion Scale (GROMS) evaluation, there were no statistically significant differences between the watsu and hydrotherapy groups (Pre-intervention p = 0.794, Post-intervention p = 0.190 and follow-up p = 0.383). Intragroup, watsu therapy significantly improved the joint range between baseline and post-intervention evaluation (p = 0.023) (table 4)."
**Notes**	"No adverse events related to therapeutic interventions were reported by the study participants or their legal guardians." Temperature ? 45 min

Risk of bias table

Bias	Authors' judgement	Support for judgement
Random sequence generation (selection bias)	Low risk	A simple randomization method was carried out in groups of 10 patients, with a 1: 1 allocation ratio, after accepting to participate in the study.
Allocation concealment (selection bias)	Low risk	Allocation concealment mechanism: The personal information of the patient was hidden by the use of an identification code (ID), then assigned to the watsu groups and conventional hydrotherapy using a random number generator. (nosetup.org). This information was placed in sealed envelopes, which were given to the patient and / or legal guardian.
Analyzed within the group originally assigned to (selection bias)	Low risk	according to flow-chart
Comparable baseline outcome measurements (selection bias)	High risk	Declared: With regard to health-related quality of life, measured through PedsQL4.0, watsu therapy presented a statistically significant difference in the physical functioning sub-dimension at the baseline level (p = 0.028) compared to conventional hydrotherapy.
Comparable baseline characteristics (selection bias)	Low risk	The study participants presented homogeneity between the watsu and hydrotherapy groups, in terms of age and subtype of JIA.The majority of the participants in the study were female (76.1%), although there were statistically significant differences (p = 0.038) in the grouping of patients according to gender in the study groups, concentrating a greater number of males in the watsu group
Control for important confounding (selection bias)	Low risk	in- and exclusion criteria clearly stated
Blinding of participants and personnel (performance bias)	Low risk	The sequence of random assignment and interventions was made by the methodological team of the Research and Development Directorate (DIDE) Telethon, the registration of participants was carried out by the main researcher. The evaluators who carried out each measurement of results (previous, subsequent and follow-up of the intervention) were blinded to the randomization and assignment of the participants in the study groups. However, given the obvious differences in interventions (watsu and conventional hydrotherapy), the patients and professionals who administered the therapies were not blinded.
Impact from a concurrent intervention (performance bias)	High risk	the continuity of their routine treatments was accepted, as scheduled physiotherapy on land, occupational therapy and changes in their pharmacological dosage due to ethical considerations.

Fidelity to the intervention protocol maintained (performance bias)	Low risk	apparently no deviations
Missing data handled appropriately (attrition bias)	High risk	"The data were analyzed on the basis of "intention to treat", so that the dependent variables of all randomized patients were included in the analysis." "It was understood as a completed treatment, the presence of 60% or more adherence to the total treatment programmed in each patient." --> according to flow-chart, not all randomized participants were included in analyses.
Incomplete outcome data (attrition bias)<br>Objecitve Outcomes	Unclear risk	insufficient information
Incomplete outcome data (attrition bias)<br>Self-reported Outcomes	Unclear risk	insufficient information
Length of follow-up (detection bias)	Low risk	procedure clearly described
Blinding of outcome assessment (detection bias)	Low risk	The evaluators who carried out each measurement of results (previous, subsequent and follow-up of the intervention) were blinded to the randomization and assignment of the participants in the study groups.<br>The professionals responsible for making the measurements were a psychologist and 2 kinesiologists trained in the use of each assessment instrument.
Intervention clearly defined (detection bias)	Low risk	yes, details provided
Outcomes assessed reliably (detection bias)	Low risk	HRQOL, functional health status, pain sensation and articular range of movement: PedsQL 4.0, CHAQ, GROMS. Psychometric properties stated.
Confounding variables assessed (detection bias)	Unclear risk	insufficient information
Potential outcomes prespecified (reporting bias)	Low risk	prespecified outcomes declared
All prespecified outcomes reported (reporting bias)	Low risk	all outcomes prespecified in methods-section reported

Ribeiro et al., 2019

**Methods**	CS
**Participants**	non-probabilistic sampling for convenience, 4 patients mean age 60.5 yrs.
**Interventions**	9 times 60 min WATSU in 5 weeks
**Outcomes**	"Regarding quality of life, a reduction in the score was observed after Watsu session, which means a better perception of the quality of life but without statistic significant (38.75 to 36.5, p=0.18). When evaluated the anxiety, it was possible to notice an increase in scores after the Watsu sessions, reflecting an increase in anxiety in these patients, but without statistical significance (17.25 to 21.75, p =

	0.58). For depression levels, even showing that patients were classified with moderate to severe depressive symptoms, a reduction of the means obtained was observed (23 to 21.25, p = 0.7). Leading to believe that there was a tendency to improve for this variable (Table 2)."<br>"In the instrumental activities of daily life, it was possible to observe an increase in the scores obtained, reflecting an increase in the independence to carry out the activities (19.50 to 20.75, p = 0.43). In the basic daily life activity, the patients are semi-dependent (7.25 to 8.25, p = 0.25). Finally, in the analysis of the balance it was possible to observe that even after the Watsu sessions the patients present a high risk of falling (20.25 to 18.25, p = 0.39) as shown in table 3."
**Notes**	35° C

Risk of bias table

Bias	Authors' judgement	Support for judgement
Random sequence generation (selection bias)	Unclear risk	n/a
Allocation concealment (selection bias)	Unclear risk	n/a
Analyzed within the group originally assigned to (selection bias)	Unclear risk	n/a
Comparable baseline outcome measurements (selection bias)	Unclear risk	n/a
Comparable baseline characteristics (selection bias)	Unclear risk	n/a
Control for important confounding (selection bias)	Low risk	non-probabilistic sampling for convenience. in- and exclusion criteria stated
Blinding of participants and personnel (performance bias)	Unclear risk	insufficient information
Impact from a concurrent intervention (performance bias)	Unclear risk	insufficient information
Fidelity to the intervention protocol maintained (performance bias)	Low risk	apparently no deviations
Missing data handled appropriately (attrition bias)	Unclear risk	insufficient information
Incomplete outcome data (attrition bias)<br>Objecitve Outcomes	Unclear risk	insufficient information
Incomplete outcome data (attrition bias)<br>Self-reported Outcomes	Unclear risk	insufficient information
Length of follow-up (detection bias)	Unclear risk	insufficient information

Blinding of outcome assessment (detection bias)	Unclear risk	insufficient information
Intervention clearly defined (detection bias)	Low risk	It was used the basic movements of the technique, as follows: Dance of the breath, offering slow, releasing the column, offering with one leg, offering with both legs, accordion, accordion with rotation, rotation with the leg from within, rotation with leg from outside, accompany the movement holding a leg (inner), algae (shoulder support), quietude and heart lullaby.
Outcomes assessed reliably (detection bias)	Low risk	BAI, BDI, Katz Index, Tinneti's Scale, PDQ-39
Confounding variables assessed (detection bias)	Unclear risk	insufficient information
Potential outcomes prespecified (reporting bias)	Unclear risk	prespecified outcomes declared
All prespecified outcomes reported (reporting bias)	Unclear risk	all outcomes prespecified in methods-section reported

Schitter & Fleckenstein, 2018

**Methods**	CR
**Participants**	A 52-year-old woman survived a severe motorcycle accident in which she sustained several fractures on the right side of her body, including ribs, the pelvis, and the femur.
**Interventions**	After discharge from stationary care, she independently scheduled 8 weekly WATSU sessions
**Outcomes**	Quantitative and qualitative data obtained from the patient's diary and the therapist's notes<br>Quantitative Outcomes<br>Of the 8 scheduled WATSU sessions, 6 were delivered. Sessions 2 and 3 were cancelled by the patient in agreement with her physician due to elevated inflammation markers. The self-estimated general, emotional, mental, and physical conditions, as reported at the beginning of each session, continuously improved while the reported medication intake decreased and the patient continuously progressed in most functional areas that she had defined as difficult in the PSFS. No systematic changes of the swelling of the knee were observed (Online Supplemental Table 1, Online Supplemental Material; www.karger.com/?DOI=487768).<br>Qualitative Outcomes<br>The patient's diary of the concerned time frame contained information concerning the WATSU sessions and observations related<br>to them (expectations, transfer effects; in total, 186 mentions) as well as information concerning physiotherapeutic and osteopathic<br>treatments, other medical interventions, the overall progress during the week, and events that were of specific significance to the<br>patient (in total, 149 mentions). Results from the analysis of qualitative data on the patient's description of her experiences, subdivided according to their content

	into the categories (1) emotional aspects, (2) mental aspects, and (3) physical aspects are presented in Online Supplemental Table 2 (Online Supplemental Material; www.karger.com/?DOI=487768). The relative frequencies of all categories derived from the patient's diary are presented in figure 1. With respect to WATSU, the patient's notes centered around emotional release, reconciliation with her body, and trunk mobilization (followed by ameliorated breath). She mentioned that WATSU added a certain quality of secureness to her rehabilitation experience and helped her to shift focus from problems and paresthesia to positive emotions and pleasant body perceptions, e.g. by re-introducing the experience of unimpeded motion and relaxation. As a result, she ascribed WATSU lasting beneficial effects on her body image. Notes not related to WATSU mainly described the daily progress and setbacks, and the duration and intensity of trainings/interventions. Reflection of the treatments based on the notes of the therapist and comments from the patient diary containing crucial additional information are summarized in Online Supplemental Table 3 (Online Supplemental Material; www.karger. com/?DOI=487768).
**Notes**	35 °C Adverse finding: swelling of knee after first session

Risk of bias table

Bias	Authors' judgement	Support for judgement
Random sequence generation (selection bias)	Unclear risk	n/a
Allocation concealment (selection bias)	Unclear risk	n/a
Analyzed within the group originally assigned to (selection bias)	Unclear risk	n/a
Comparable baseline outcome measurements (selection bias)	Unclear risk	n/a
Comparable baseline characteristics (selection bias)	Unclear risk	n/a
Control for important confounding (selection bias)	High risk	"2 and 3 were cancelled by the patient in agreement with her physician due to elevated inflammation markers."
Blinding of participants and personnel (performance bias)	Unclear risk	n/a
Impact from a concurrent intervention (performance bias)	High risk	"rehabilitative outpatient program. It initially contained physiotherapy 2–3 times a week, as prescribed by the surgeon who provided aftercare, and autonomously added WATSU on a weekly basis. From week 13 post accident onwards, the patient also chose to utilize monthly osteopathic consultations."

Fidelity to the intervention protocol maintained (performance bias)	Low risk	deviations reported: "2 and 3 were cancelled by the patient in agreement with her physician due to elevated inflammation markers."
Missing data handled appropriately (attrition bias)	Low risk	all data presented as supplementary material
Incomplete outcome data (attrition bias)<br>Objecitve Outcomes	Low risk	all data presented as supplementary material
Incomplete outcome data (attrition bias)<br>Self-reported Outcomes	Low risk	all data presented as supplementary material
Length of follow-up (detection bias)	Unclear risk	n/a
Blinding of outcome assessment (detection bias)	High risk	notes of the therapist who delivered WATSU
Intervention clearly defined (detection bias)	Low risk	yes, videos in supplementary material
Outcomes assessed reliably (detection bias)	Low risk	however: NRS, PSFS, circumference knee: validity not reported. Diary of patient and notes of therapist
Confounding variables assessed (detection bias)	Low risk	reported: circumference knee, medication, other treatments
Potential outcomes prespecified (reporting bias)	Low risk	prespecified outcomes declared
All prespecified outcomes reported (reporting bias)	Low risk	all outcomes prespecified in methods-section reported

Schitter et al., 2015

**Methods**	CT
**Participants**	Treatment: Nine healthy pregnant women at gestational week ≥ 34 were included<br>Control: eight women in a passive control group
**Interventions**	WATSU was performed on days 1 and 4 of the study, accompanied by ultrasound examinations<br>The administered motion sequence "WATSU-Transition-Flow" [9] was adapted for women in their third trimester of pregnancy and was followed closely. 60min
**Outcomes**	physiological and psychometric as well as qualitative data<br>medium term: Days 1 and 8:<br>PSS (Perceived Stress Scale)<br>SF-36 (quality of life in domains of physical function, bodily pain, vitality, social function, role physical, general health, role emotional, mental health)<br>medium term, within intervention group:<br>Variables (a) At baseline (day 1) At follow-up (day 8) /Δ value P (b, *sig)<br>PSS score (c) 14.8 (±5.0) 12.7 (±5.3) /– 2.1 (±2.2) 0.027∗<br>SF-36 main scales (d)<br>(i) Physical component 47.0 (±6.8) 45.3 (±11.0) /– 1.7 (±6.7) 0.735<br>(ii) Mental component 46.4 (±11.6) 50.0 (±12.3) /+3.3 (±4.0) 0.018∗

	SF-36 sub scalesd<br>(i) Physical function 58.9 (±11.4) 61.1 (±18.7) /+2.2 (±10.9) 0.527<br>(ii) Role physical 72.2 (±49.1) 66.7 (±43.3) /– 5.6 (±48.1) 1.00<br>(iii) Bodily pain 72.7 (±49.2) 70.4 (±26.3) /– 2.2 (±4.1) 0.157<br>(iv) General health 81.3 (±10.8) 79.7 (±17.4) /– 2.4 (±13.8) 0.786<br>(v) Vitality 50.6 (±18.3) 55.6 (±18.3) /+5.0 (±9.0) 0.114<br>(vi) Social function 76.4 (±22.1) 79.2 (±21.7) /+2.8 (±8.3) 0.317<br>(vii) Role emotional 63.0 (±38.9) 70.4 (±38.9) /+7.4 (±22.2) 0.317<br>(viii) Mental health 67.1 (±16.9) 72.0 (±18.6) /+4.9 (±8.4) 0.125<br>a: All continuous data are presented as mean (±SD).<br>b: values refer toWilcoxon test. ∗ P > 0.05; CI 95%.<br>c: PSS, Perceived Stress Scale; positive mean change values represent an increase in perceived stress.<br>d: SF-36, Medical Outcomes Study 36-Item Short-Form Health Survey; positive mean change values represent an increase in health related quality of life.<br>medium term, between groups: participants in the intervention group reported reduction in perceived stress from day 1 to day 8 (P = 0.036, Cohen's f = 0.57):<br>Variables (a) ∆ values From baseline (day 1) to follow-up (day 8), P (b) Cohen's f<br>Intervention group / Control group:<br>PSS score (c) – 2.1 (±2.2) +.9 (±3.2) 0.036∗ 0.57<br>SF-36 main scales (d)<br>(i) Physical component – 1.6 (±6.6) +.2 (±6.4) 0.500<br>(ii) Mental component +3.3 (±4.0) +3.1 (±3.6) 0.923<br>a: All continuous data are presented as mean (SD).<br>b: P-values refer to Mann-Whitney test. ∗ P > 0.05; CI 95%.<br>c: PSS, Perceived Stress Scale; positive mean change values represent an increase in perceived stress.<br>d: SF-36, Medical Outcomes Study 36-Item Short-Form Health Survey; positive mean change values represent an increase in health related quality of life.<br>Days 1 and 4, pre/post:<br>Stress and Pain Related Visual Analogue Scales (VAS)<br>Multidimensional Mood Questionnaire (MDMQ)<br>within intervention group:<br>Variables (a) Before WATSU After WATSU /∆ value P (b)<br>VAS stressfulness (mm) (c)<br>(i) At day 1 27 (±28) 5 (±9) /– 21 (±22) 0.028∗<br>(ii) At day 4 16 (±15) 2 (±2) /– 14 (±14) 0.012∗<br>VAS pain (mm)c<br>(i) At day 1 9 (±12) 2 (±3) /– 8 (±9) 0.028∗<br>(ii) At day 4 12 (±10) 1 (±1) /– 11 (±11) 0.012∗<br>MDMQ mood scale score (d)<br>(i) At day 1 16.9 (±2.8) 18.7 (±2.6) /+1.8 (±1.9) 0.042∗<br>(ii) At day 4 14.7 (±6.8) 19.3 (±1.0) /+4.7 (±6.2) 0.027∗<br>MDMQ alertness scale score (d)<br>(i) At day 1 13.9 (±3.2) 16.0 (±2.4) /+2.1 (±3.6) 0.122<br>(ii) At day 4 10.7 (±5.0) 16.3 (±2.4) /+5.7 (±4.7) 0.007∗<br>MDMQ calmness scale score (d)<br>(i) At day 1 13.7 (±3.0) 18.0 (±2.9) /+4.3 (±3.0) 0.012∗<br>(ii) At day 4 12.8 (±5.6) 18.3 (±1.7) /+5.6 (±5.8) 0.015∗<br>a: All continuous data are presented as mean (±SD).<br>b: P-values refer toWilcoxon test. ∗ P > 0.05; CI 95%.

	c: VAS, Visual Analog Scale; positive mean change values represent an increase in actual stress (actual pain).<br>d: MDMQ, Multidimensional-Mood-Questionnaire; positive mean change values represent an increase in mood, alertness, and calmness.<br>between groups:<br>Variables (a) Δ values Intervention group before and after WATSU,<br>Control group before and after 2 hours waiting period, P (b) Cohen's f<br>VAS stressfulness (mm) (c)<br>(i) At day 1 − 21 (±22) − 3 (±11) 0.090<br>(ii) At day 4 − 14 (±14) +1 (±12) 0.021∗ 0.61<br>VAS pain (mm) (c)<br>(i) At day 1 − 8 (±9) − 1 (±4) 0.037∗ 0.51<br>(ii) At day 4 − 11 (±11) +3 (±10) 0.005∗ 0.72<br>MDMQ mood scale score (d)<br>(i) At day 1 +1.8 (±1.9) +0.5 (±2.51) 0.175<br>(ii) At day 4 +4.7 (±6.2) − 0.1 (±1.13) 0.013∗ 0.56<br>MDMQ alertness scale score (d)<br>(i) At day 1 +2.1 (±3.6) +0.1 (±1.2) 0.200<br>(ii) At day 4 +5.7 (±4.7) +0.4 (±1.2) 0.001∗ 0.79<br>MDMQ calmness scale score (d)<br>(i) At day 1 4.6 (±3.0) − 0.3 (±1.9) 0.002∗ 0.96<br>(ii) At day 4 5.6 (±5.8) − 0.5 (±2.2) 0.008∗ 0.71<br>a: All continuous data are presented as mean (SD).<br>b: P-values refer to Mann-Whitney test. ∗ P > 0.05; CI 95%.<br>c: VAS, Visual Analog Scale; positive mean change values represent an increase in actual stress (actual pain).<br>d: MDMQ, Multidimensional-Mood-Questionnaire; positive mean change values represent an increase in mood, alertness, and calmness.<br>Days 1, 4 and 8, pre/post:<br>Ultrasound Examination of fetal position and assessment of amniotic fluid volume: Four participants with lowbaseline values (≤ 3.8 cm,mean 3.6 cm ± 0.1 cm) showed an average increase in amniotic fluid to 5.9 cm ± 1.5 cm on day 8. For one participant, the values remained practically unchanged (from5.5 cmto 5.6 cm), and in four cases that presented values ≥ 5.6 cm (6.8 cm ± 0.8 cm) at baseline, the amniotic fluid value decreased to amean value of 5.02 cm ± 0.7 cm. Similarly, the pulsatility indexes (PI) obtained from the umbilical artery and from both maternal vessels were within normal ranges for the respective gestational age and no significant changes were observed comparing pre- and posttreatment values (P-values ≥ 0.23). No signs of adverse reactions were detectable in ultrasound measures at any point in the study.<br>Due to optimal tonus of the uterus, four of the participants in the intervention group underwent cephalic version on day 9 of the study, with two attempts being successful. Of the eight fetuses in breech position in the intervention group, one spontaneously presented in cephalic position on day 4. The two fetuses in breech in the control group remained in this position up to day 8 and one attempt for cephalic version failed.<br>Qualitative data indicate that WATSU was appreciated as enjoyable and deeply relaxing.

**Notes**	Temperature: 35 °C "No adverse events were reported, and all of the WATSU-treatments were carried out as scheduled."

Risk of bias table

Bias	Authors' judgement	Support for judgement
Random sequence generation (selection bias)	Unclear risk	n/a
Allocation concealment (selection bias)	Unclear risk	n/a
Analyzed within the group originally assigned to (selection bias)	Low risk	yes, flow chart provided
Comparable baseline outcome measurements (selection bias)	Low risk	"Group and baseline characteristics did not differ significantly between the two study groups"
Comparable baseline characteristics (selection bias)	High risk	Not statistically evaluated: Intervention group with 8 women with breech position compared to 2 in control group
Control for important confounding (selection bias)	Low risk	in- and exclusion criteria clearly stated: "Interested healthy women with a singleton pregnancy in the 34th or greater week of gestation underwent a telephonescreening. Exclusion criteria were any pathological findings during pregnancy, neurological deficits resulting from low back pain,WATSU-treatment within the past fourweeks, and poor language skills.Women reporting a breech presentation were included in the study if they did not plan external cervical version." 4 different therapists provided the treatments.<br>Possibly confounding: at the end of pregnancy, discomfort may pronounce prarallel to fetal growth (however, in both groups)
Blinding of participants and personnel (performance bias)	Low risk	Statistician and outcome assessors blinded
Impact from a concurrent intervention (performance bias)	High risk	All participants were free to maintain additional medical and/or therapeutic treatments during the study.
Fidelity to the intervention protocol maintained (performance bias)	High risk	deviations from protocol disclosed: "The original study design implied ultrasound examinations on the control group as well to assess the natural course of changes in the amount of amniotic fluid [59]. In fact, only one woman in the control group agreed to follow this procedure."
Missing data handled appropriately (attrition bias)	Low risk	"Quantitative data were analyzed based on intention to treat. For missing data, the last value was carried forward"
Incomplete outcome data (attrition bias)<br>Objecitve Outcomes	Low risk	missing data disclosed: Ultrasound Examination; in control-group only one person measured

Incomplete outcome data (attrition bias)<br>Self-reported Outcomes	Low risk	"Quantitative data were analyzed based on intention to treat. For missing data, the last value was carried forward."
Length of follow-up (detection bias)	Low risk	standardized procedure reported
Blinding of outcome assessment (detection bias)	Low risk	assessors of ultra-sound and statistician blinded
Intervention clearly defined (detection bias)	Low risk	clearly described, "The administered motion sequence “WATSU-Transition-Flow” [9] was adapted for women int heir third trimester of pregnancy and was followed closely." Sessions lasted 60 minutes and took place in the morning from 9:00 a.m. to 10:00 a.m.
Outcomes assessed reliably (detection bias)	Low risk	yes
Confounding variables assessed (detection bias)	Low risk	Baseline assessment clearly described, Following the session, participants were asked to drink 500mL of water to compensate for body fluid loss due to increased diuresis
Potential outcomes prespecified (reporting bias)	Low risk	prespecified outcomes declared
All prespecified outcomes reported (reporting bias)	Low risk	all outcomes prespecified in methods-section reported

Tufekcioglu et al., 2018

**Methods**	RCT
**Participants**	34 f and m, age 8.30±.31, obese with BMI above 30, no other chronic health issues, non-smokers, no alcohol
**Interventions**	Intervention: WATSU (n=13) compared to<br>Control: mere immersion in shoulder deep water (n=11) and no intervention (=10). 30 minutes, 12-week of watsu and immersion therapies<br>Each watsu therapy session started with a brief verbal description of the procedure. W.A.B.A. (Worldwide Aquatic Bodywork Association) certified professional watsu practitioner applied watsu-2 therapy protocol that includes stretches, trunk rotation, joint mobilization, vertebral traction/lengthening, deep breathing and pressure point work and rocking techniques in water.<br>Immersion group spent the same time immersed without movement and/or receiving any therapy in shoulder depth warm water. Immersion and watsu therapies were performed for 30 minutes, twice a week for 12 weeks, between 5:00-7:00 pm in an indoor swimming pool. Temperatures of the pool water and air were 32°C and 29°C. Post-intervention: The aforementioned procedure for the baseline data collection was performed one day after the experimental therapy protocol.
**Outcomes**	A significant inverse correlation was found between HRV values and both therapeutic interventions. 12-week of watsu and immersion therapies decreased LF and HF. The collective results of HF showed significant negative effect (13.01±1.36, 9.99±1.11) and HF laying supine value (20.62±2.22) was the highest (p<0.05).

	In detail:<br>No significant differences were observed in LF N.U. of each group: Watsu=29.84±1.80, immersion=31.63±1.96, control=30.35±2.05 (p>0.05). The LF value showed an insignificant increase in Immersion group cycling (non locomotor) and Control group laying supine (horizontal) position (p>0.05). The pre to posttest changes are similar for the groups in each circumstances.<br>There was no difference between the groups VLF ms2 (nu) values: Watsu®=58.13±2.71, immersion=57.78±2.95, Control=57.72±3.09 (p>0.05). Collective result of VLF ms2 (nu) increased from 53.65±2.06 to 62.09±1.90 (p<0.05). This is similar in each group (p>0.05). There was a gradual increase in order of four collective recording conditions VLF ms2: 50.53±2.46, 58.56±1.84, 60.24±2.74, 62.16±2.53 respectively. The only significance was observed between horizontal and other recording conditions (p<0.05). These changes are similar in each group.<br>VLF ms2 result of each groups in each conditions are similar (p>0.05)
**Notes**	32 °C, BP measured but not reported

Risk of bias table

**Bias**	**Authors' judgement**	**Support for judgement**
Random sequence generation (selection bias)	High risk	insufficient information, different sizes of groups (n=10, n=11, n=13) are suspicious, no drop-outs reported
Allocation concealment (selection bias)	Unclear risk	insufficient information
Analyzed within the group originally assigned to (selection bias)	Unclear risk	insufficient information
Comparable baseline outcome measurements (selection bias)	Low risk	"No significant differences (p>0.05) were seen in the baseline recordings of HF N.U. values of each groups (Watsu=11.99±1.68, Immersion=10.54±1.83, Control=11.99±1.92)."
Comparable baseline characteristics (selection bias)	Low risk	Group Age (year) Height (cm) Weight (kg) BMI (w/h2)<br>Watsu (n=13) 18.16±.27 171.31±6.54 108.05±20.52 36.58±5.58<br>Immersion (n=11) 18.34±.34 170.27±5.39 110.40±23.61 38.01±8.03<br>Control (n=10) 18.45±.26 154.46±53.96 102.16±15.17 34.86±3.34<br>Total (N=34) 18.30±.31 166.02±29.60 107.08±19.90 36.54±5.96
Control for important confounding (selection bias)	Low risk	"Possible influences of diet and energy intake were not examined in this study." However: "Low power filter were performed in order to eliminate the noise in the recordings and then HRV indices in frequency domain were obtained using Kubios HRV 2.2 (bio-signal analyzing software)."

Blinding of participants and personnel (performance bias)	Unclear risk	"Both participants and assessors were not blinded."
Impact from a concurrent intervention (performance bias)	Unclear risk	insufficient information
Fidelity to the intervention protocol maintained (performance bias)	Unclear risk	insufficient information, different sizes of groups are suspicious
Missing data handled appropriately (attrition bias)	Unclear risk	insufficient information, different sizes of groups are suspicious, no drop-outs reported
Incomplete outcome data (attrition bias)<br>Objecitve Outcomes	Unclear risk	insufficient information, different sizes of groups are suspicious, no drop-outs reported
Incomplete outcome data (attrition bias)<br>Self-reported Outcomes	Unclear risk	n/a
Length of follow-up (detection bias)	Low risk	post-measurement one day after completion of 12 week intervention period
Blinding of outcome assessment (detection bias)	High risk	"Both participants and assessors were not blinded."
Intervention clearly defined (detection bias)	Low risk	yes
Outcomes assessed reliably (detection bias)	Low risk	yes<br>"We applied the following same procedure one day before and after the experimental therapy period. BP (blood pressure) was measured after 10 minutes rest in a silent room, right before HRV data recordings. A baseline HRV was recorded 5 minutes in each circumstances: 1. Laying supine (horizontal). 2. Sitting (vertical). 3. Walking (locomotor). 4. Cycling (non locomotor). All participants maintained food intake routine, avoided exercise and caffeine consumption during the previous 24 hours preceding the measurements.<br>Participants consumed 500 mL of water 2 hours before the measurements. Polar H7 heart rate sensor and digital standardized HRV signal processing software called HRV+ were used to record the R-R intervals in millisecond. iPad-2 tablet pc that fully supports Bluetooth Smart (Bluetooth 4.0) were used for screening R-R intervals in ms. A Landice, model L-7 treadmill was used for locomotor HRV recordings at 4.5 km/hr walking speed. A Monark, model 686 cycle ergometer was used for non-locomotor HRV recordings at 50 W pedaling resistance and at 55 rpm. The obtained ECG data files were then transmitted to a personal computer as txt files. The frequency domain indices of HRV (VLFMS2, LF and HF N.U.) were calculated.<br><br>The measurements were initiated after participants confirmed positive feelings of four or more points on the

		5-point scale. The following steps reinforced quality control of data collection: 1. Spectra 360 Electrode Gel was used on the skin before attaching the Polar H7 heart rate sensor for the continuity of precise signal detection. 2. Low power filter were performed in order to eliminate the noise in the recordings and then HRV indices in frequency domain were obtained using Kubios HRV 2.2 (bio-signal analyzing software)."
Confounding variables assessed (detection bias)	Unclear risk	insufficient information "Low power filter were performed in order to eliminate the noise in the recordings and then HRV indices in frequency domain were obtained using Kubios HRV 2.2 (bio-signal analyzing software)."
Potential outcomes prespecified (reporting bias)	Low risk	prespecified outcomes declared
All prespecified outcomes reported (reporting bias)	Unclear risk	Blood pressure measured but not reported in detail.

Wieser, 2007

**Methods**	CS, narratively
**Participants**	19 participants, 9 students were classified as autistic; the other 10 students were classified as severe and profound multiple handicap.<br>Ages of the 19 project participants ranged from 6 to 21 years of age.
**Interventions**	twice a week, alternating once in the gym and once in the pool, for 30 minutes. In the gym:group work with whole class. In addition to adapted physical education, many of the children also receive speech therapy, physical therapy and occupational therapy.<br>WATSU: not follow a specific sequence, but I always began by loosening the hips and spine. Five basic modifications to the WATSU techniques over the course of the project. Floatation devices when necessary to maintain head above water for those students who lacked head control or who made sudden unpredictable head movements. Quiet, soothing talk and eye contact to make a connection with the student. Usually began the session with the Under Head, or Seaweed position; most of the time the session started from a vertical position rather than a horizontal position. Although I used continuous movement, speed of movement varied with responses of the child. Finally, I did not use a specified sequence of WATSU movements. Rather, using various WATSU positions and transitions I developed an interactive “dance” with each student.
**Outcomes**	Outcomes not measured, only descriptive, qualitative: Overall, the children became more flexible, and showed greater range of motion. The children appeared calmer,less aggressive, and demonstrated an increased focus and willingness to try new activities. very beneficial for the children.
**Notes**	Temperature: "warm". No firm conclusions can be drawn to effects of WATSU.

Risk of bias table

Bias	Authors' judgement	Support for judgement
Random sequence generation (selection bias)	Unclear risk	n/a
Allocation concealment (selection bias)	Unclear risk	n/a
Analyzed within the group originally assigned to (selection bias)	Unclear risk	n/a
Comparable baseline outcome measurements (selection bias)	Unclear risk	n/a
Comparable baseline characteristics (selection bias)	Unclear risk	n/a
Control for important confounding (selection bias)	Unclear risk	insufficient information
Blinding of participants and personnel (performance bias)	Unclear risk	n/a
Impact from a concurrent intervention (performance bias)	High risk	WATSU is one of several therapies in the reported setting
Fidelity to the intervention protocol maintained (performance bias)	Unclear risk	n/a
Missing data handled appropriately (attrition bias)	Unclear risk	n/a
Incomplete outcome data (attrition bias) Objecitve Outcomes	Unclear risk	likely, narrative summary only
Incomplete outcome data (attrition bias) Self-reported Outcomes	Unclear risk	n/a
Length of follow-up (detection bias)	Unclear risk	n/a
Blinding of outcome assessment (detection bias)	High risk	no, personal observations recalled
Intervention clearly defined (detection bias)	Low risk	(temperature not stated)
Outcomes assessed reliably (detection bias)	High risk	no
Confounding variables assessed (detection bias)	Unclear risk	insufficient information
Potential outcomes prespecified (reporting bias)	High risk	no
All prespecified outcomes reported (reporting bias)	Unclear risk	n/a

Footnotes
