# Supplementary material for: Applications, indications, and effects of passive hydrotherapy WATSU (WaterShiatsu)—A systematic review and meta-analysis
Source: PLoS One. 2020 Mar 13;15(3):e0229705. doi: 10.1371/journal.pone.0229705 (PMC7069616; doi:10.1371/journal.pone.0229705)
Supplement: S2 File — (PDF) [file pone.0229705.s007.pdf]

Applications, Indications, and Effects of Passive Hydrotherapy WATSU

References to studies

Included studies

Antunes et al., 2016

Antunes MD, Vertuan MP, Miquilin A, Leme DEdC, Morales RC, Oliveira DVd. Effects of Watsu on the quality of life and pain in elderly woman with fibromyalgia [Efeitos do Watsu na qualidade de vida e quadro doloroso de idosas com fibromialgia]. Consciência e Saúde 2016;15(4):636-641. [DOI: 10.5585/ConsSaude.v15n4.6756]

Barbosa et al., 2014

Barbosa GAS, Rocha COM, Resende CMBMd, Sales KVdM. Effect of Watsu therapy on psychological aspects and quality of life of patients with temporomandibular disorder: case report [Efeito da terapia Watsu sobre os aspectos psicológicos e a qualidade de vida em pacientes com disfunções temporo-mandibulares: relato de caso]. Revista Gaúcha de Odontologia 2014;62(3):309-13. [DOI: 10.1590/1981-863720140003000122409]

Borges & Parizotto, 2001

Borges RM, Parizotto NA. Physiological analysis in stressed patients submitted to Watsu water therapy [Análise dos efeitos fisiológicos em pacientes comestresse submetidos à técnica Watsu]. Fisioterapia Brasil 2001;2(1):33-40. [Other: ISSN: 1518-9740]

Campos et al., 2018

Campos NSd, Landim ACLP, Teixera MP, Rodrigues JM, Grosskreutz TdS, Schrader EP. Aquatic relaxation and the control of occupational stress in workers of a university hospital [Relaxamento aquático no controle do estresse ocupacional em trabalhadoras de um hospital universitário]. In: Revista Científica JOPEF. Vol. 25. 2018. [Other: ISSN 1806-1508]

Chen et al., 2018

Chen O, Grabarnick A, Pilz-Burstein R. The effect of Watsu treatments on pain indices and on the quality of sleep in women diagnosed as fibromyalgia patients. Journal of the Israeli Physical Therapy Society (JIPTS) 2018;20(2):14-24.

Chon et al., 2009

Chon SC, Oh DW, Shim JH. Watsu approach for improving spasticity and ambulatory function in hemiparetic patients with stroke. Physiotherapy Research International 2009;14(2):128-36. [DOI: 10.1002/pri.421]

Chun et al., 2006

Chun S-C, Yoon S-I, Oh D-W, Shim J-H, Lee G-W, An C-S. The effect of submerged relaxation exercise on muscle tone in persons with hemiparesis. Physical Therapy Korea 2006;13(3):84-91.

Cunha et al., 2010

Cunha MGd, Carvaho EVd, Caromano FA. Effects of single session of Watsu [Efeitos da sessão única de WATSU]. Cadernos de Pós-Graduação em Distúrbios do Desenvolvimento 2010;10(1):103-9.

Dornelas, 2011

Dornelas LdF. Hydrotherapy techniques in a case of Jarcho-Levin [Técnicas de Hidroterapia em um caso de Jarcho-Levin]. Revista Neurociencia 2011;19(1):85-9.

Faull, 2005

Faull K. A pilot study of the comparative effectiveness of two water-based treatments for fibromyalgia syndrome: Watsu and Air massage. Journal of Bodywork & Movement Therapies 2005;9(3):202-10. [DOI: 10.1016/j.jbmt.2004.12.001]

Gimenes et al., 2006

Gimenes OR, Santos EC, Silva TJPV. Watsu in the treatment of fibromyalgia: pilot study [Watsu no Tratamento da Fibromialgia: Estudo Piloto]. Revista Brasileira de Reumatologia 2006;46(1):75-6.

Gimenez & Castro, 2018

Gimenez MdF, Castro NHSd. Effect of watsu technique on the quality of sleep and spasticity in teenagers with cerebral paralysis: Case series [Efeito da técnica de watsu na qualidade do sono e espasticidade em adolescentes com paralisia cerebral: Série de casos]. Unisanta Health Science 2018;2(1):42-50.

Hora et al., 2017

Hora CRB, Santos LdS, Lima MIdS, Gusmão TMRd, Ribeiro ASC. Physiological variations and subjective feelings of disciplines from the tiradentes - al university center submitted to a single watsu session [Variações fisiológicas e sensaçõessubjetivas de discentes, do centro universitário tiradentes - al, submetidos a uma única sessão de watsu]. Ciências Biológicas e de Saúde Unit | Aracaju 2017;4(1):31-42. [Other: ISSN ELETRÔNICO 2316-3143]

Israel et al., 2006

Israel VL, Arruda ES, Paolini AP, Barbosa AD, Shmeil A, Flach CRC. Physical Therapy: Principles of the WATSU in Patients with Ankylosing Spondylitis. In: Karagülle MZ, Dönmez A, editor(s). 35th Congress of the International Society of Medical Hydrology & Climatology. Istanbul, 2006.

Jithin & Adarsh, 2019

Jithin TA, Adarsh M. Effect of watsu water relaxation training on selected physiological variables among school level swimmers. International Journal of Scientific Research 2019;8(8):35-36. [Other: PRINT ISSN No. 2277 - 8179]

Lima et al., 2009

Lima AARd, Pereira KdS, Vinhas R. Effect of the Watsu method in moderate asthmatic patient - case report [Efeitos do método Watsu em paciente asmático moderado - relato de caso]. Revista Neurociencias 2009;17(3):283-6.

Mota et al., 2007

Mota NM, Silva VH, Lima JS, Oliveira LVF. Study of autonomic nervous activity by analysis of variability of cardiac frequency in subjects submitted to WATSU therapy [Estudo da Atividade Nervosa Autonômica por Meio daAnálise da Variabilidade da Freqüência Cardíaca emSujeitos Submetidos à Terapia Watsu]. Revista UniVap 2006;14(26):1343-1346.

Nakamoto, 2016

Nakamoto BV. Watsu® therapy efficacy as a guide for prevention of elderly falls: A case study [A eficácia da terapia de watsu® como indicação para prevenção de quedas a idosos: Um estudo de caso]. In: II Brazilian

and International Congress of Aquatic Physiotherapy - ABFA. Sao Caetano do Sul - SP - Brazil, 2016.

Pastrello et al., 2009

Pastrello FHH, Garcão DC, Pereira K. Watsu method as complementary resort in the physiotherapy treatment of spastic cerebral palsy tetraparetic: study of the case [Método Watsu como recurso complementar no tratamento fisioterapêutico de uma criança com paralisia cerebral tetraparética espástica: estudo de caso]. Fisioterapia em Movimento 2009;22(1):95-102. [Other: ISSN 0103-5150]

Pinkalsky et al., 2011

Pinkalsky A, Thuler PAT, Brech GC, Cunha MCB. The benefits of Watsu in the treatment of chronic pain and quality of life of patients with fibromyalgia [Os benefícios do Watsu no tratamento da dor crônica e qualidade de vida de pacientes fi bromiálgicos]. Fisiotherapia Brasil 2011;12(1):4-8. [Other: ISSN 1518-9740]

Rambo & Filippin, 2019

Rambo DC, Filippin NT. Effects of aquatic physical therapy in preterm infants admitted to the neonatal intensive care unit [Efeitos da fisioterapia aquática em prematuros internados na unidade de terapia intensiva neonatal]. In: 6. Congresso internacional em saúde. Ijuí, Rio Grande do Sul, Brasil.

Ramirez et al., 2019

Ramírez NP, Cares PN, Peñailillo PSM. Effectiveness of watsu therapy in patients with juvenile idiopathic arthritis. A parallel, randomized, controlled and single-blind clinical trial [Efectividad de la terapia watsu en pacientes con artritis idiopática juvenil. Un ensayo clínico controlado paralelo, aleatorio y simple ciego]. Revista Chilena de Pediatría 2019;90(3):283-292. [DOI: 10.32641/rchped.v90i3.886]

Ribeiro et al., 2019

Ribeiro IR, Lima FLC, Rosa TB, Santos RM, Costa SAF, Souza DM, Dias GAS, Santos CAS. The effects of the watsu method on functional capacity, anxiety and depression in patients with parkinson disease. Archives in Biosciences & Health 2019;1(1):113-124.

Schitter & Fleckenstein, 2018

Schitter AM, Fleckenstein J. Passive Hydrotherapy WATSU® for Rehabilitation of an Accident Survivor: A Prospective Case Report. Complementary Medicine Research 2018;25(4):263-268. [Other: 10.1159/000487768]

Schitter et al., 2015

Schitter AM, Nedeljkovic M, Baur H, Fleckenstein J, Raio L. Effects of passive hydrotherapy WATSU (WaterShiatsu) in the third trimester of pregnancy: Results of a controlled pilot study. Evidence-based complementary and alternative medicine: eCAM 2015;1-10. [DOI: 10.1155/2015/437650]

Tufekcioglu et al., 2018

Tufekcioglu E, Erzeybek MS, Kaya T, Ozan G. The Effect of 12-Week Passive Aquatic Bodywork on Sympathovagal Balance of Obese Youth. Journal of Education and Training Studies 2018;6(2):166-176. [DOI: 10.11114/jets.v6i2.2963]

Wieser, 2007

Wieser A. WATSU for children with severe and profound disabilities. Aquatic Therapy Journal 2007;9(2):9-13.
