# Supplementary material for: Applications, indications, and effects of passive hydrotherapy WATSU (WaterShiatsu)—A systematic review and meta-analysis
Source: PLoS One. 2020 Mar 13;15(3):e0229705. doi: 10.1371/journal.pone.0229705 (PMC7069616; doi:10.1371/journal.pone.0229705)
Supplement: S3 File — (PDF) [file pone.0229705.s008.pdf]

Applications, Indications, and Effects of Passive Hydrotherapy WATSU

Characteristics of studies

Characteristics of excluded studies

Araújo & Assis, 2018x

**Reason for exclusion**	Mixed aquatic interventions, no conclusions can be drawn to WATSU. For details see "Characteristics of studies awaiting classification"

Becker & Lynch, 2017x

**Reason for exclusion**	Mixed aquatic interventions, no conclusions can be drawn to WATSU. For details see "Characteristics of studies awaiting classification"

Bonetti et al., 2010x

**Reason for exclusion**	Mixed aquatic interventions, no conclusions can be drawn to WATSU. For details see "Characteristics of studies awaiting classification"

Cantos & Souza, 2013x

**Reason for exclusion**	Mixed aquatic interventions, no conclusions can be drawn to WATSU. For details see "Characteristics of studies awaiting classification"

Cantos et al., 2008x

**Reason for exclusion**	Mixed aquatic interventions, no conclusions can be drawn to WATSU. For details see "Characteristics of studies awaiting classification"

Cantos et al., 2008xa

**Reason for exclusion**	Mixed aquatic interventions, no conclusions can be drawn to WATSU. For details see "Characteristics of studies awaiting classification"

Cantos et al., 2013x

**Reason for exclusion**	Mixed aquatic interventions, no conclusions can be drawn to WATSU. For details see "Characteristics of studies awaiting classification"

Chang et al., 2009x

**Reason for exclusion**	Mixed aquatic interventions, no conclusions can be drawn to WATSU. For details see "Characteristics of studies awaiting classification"

Costa et al., 2017x

**Reason for exclusion**	Mixed aquatic interventions, no conclusions can be drawn to WATSU. For details see "Characteristics of studies awaiting classification"

Degani & Villa, 2005x

**Reason for exclusion**	Mixed aquatic interventions, no conclusions can be drawn to WATSU. For details see "Characteristics of studies awaiting classification"

Elsner et al., 2009x

**Reason for exclusion**	Mixed aquatic interventions, no conclusions can be drawn to WATSU. For details see "Characteristics of studies awaiting classification"

Eo & Lee, 2011x

**Reason for exclusion**	Mixed aquatic interventions, no conclusions can be drawn to WATSU. For details see "Characteristics of studies awaiting classification"

Ferreira & Matsutani, 2006x

**Reason for exclusion**	Mixed aquatic interventions, no conclusions can be drawn to WATSU. For details see "Characteristics of studies awaiting classification"

Gimenes et al., 2007x

**Reason for exclusion**	Mixed aquatic interventions, no conclusions can be drawn to WATSU. For details see "Characteristics of studies awaiting classification"

Gimenes et al., 2008x

**Reason for exclusion**	Mixed aquatic interventions, no conclusions can be drawn to WATSU. For details see "Characteristics of studies awaiting classification"

Gimenes et al., 2008xa

**Reason for exclusion**	Mixed aquatic interventions, no conclusions can be drawn to WATSU. For details see "Characteristics of studies awaiting classification"

Gonçalves et al., 2009x

**Reason for exclusion**	Mixed aquatic interventions, no conclusions can be drawn to WATSU. For details see "Characteristics of studies awaiting classification"

Jacintho et al., 2008x

**Reason for exclusion**	Mixed aquatic interventions, no conclusions can be drawn to WATSU. For details see "Characteristics of studies awaiting classification"

Jakaitis & Guazelli, 2005x

**Reason for exclusion**	Mixed aquatic interventions, no conclusions can be drawn to WATSU. For details see "Characteristics of studies awaiting classification"

Kakihara & Neves, 2005x

**Reason for exclusion**	Mixed aquatic interventions, no conclusions can be drawn to WATSU. For details see "Characteristics of studies awaiting classification"

Kim & Lee, 2015x

**Reason for exclusion**	Mixed aquatic interventions, no conclusions can be drawn to WATSU. For details see "Characteristics of studies awaiting classification"

Kim et al., 2009x

**Reason for exclusion**	Mixed aquatic interventions, no conclusions can be drawn to WATSU. For details see "Characteristics of studies awaiting classification"

Kwangmin et al., 2016x

**Reason for exclusion**	Mixed aquatic interventions, no conclusions can be drawn to WATSU. For details see "Characteristics of studies awaiting classification"

Lee & Kim, 2015x

**Reason for exclusion**	Mixed aquatic interventions, no conclusions can be drawn to WATSU. For details see "Characteristics of studies awaiting classification"

Lotan & Barmatz, 2009x

**Reason for exclusion**	Mixed aquatic interventions, no conclusions can be drawn to WATSU. For details see "Characteristics of studies awaiting classification"

Maczkowiak et al., 2007x

**Reason for exclusion**	Mixed aquatic interventions, no conclusions can be drawn to WATSU. For details see "Characteristics of studies awaiting classification"

Martins et al., 2015x

**Reason for exclusion**	Mixed aquatic interventions, no conclusions can be drawn to WATSU. For details see "Characteristics of studies awaiting classification"

Masselli et al., 2009x

**Reason for exclusion**	Mixed aquatic interventions, no conclusions can be drawn to WATSU. For details see "Characteristics of studies awaiting classification"

Mazetto & Navarro, 2007x

**Reason for exclusion**	Mixed aquatic interventions, no conclusions can be drawn to WATSU. For details see "Characteristics of studies awaiting classification"

Melo et al., 2012x

**Reason for exclusion**	Mixed aquatic interventions, no conclusions can be drawn to WATSU. For details see "Characteristics of studies awaiting classification"

Mesquita et al., 2007x

**Reason for exclusion**	Mixed aquatic interventions, no conclusions can be drawn to WATSU. For details see "Characteristics of studies awaiting classification"

Nascimento et al., 2012x

**Reason for exclusion**	Mixed aquatic interventions, no conclusions can be drawn to WATSU. For details see "Characteristics of studies awaiting classification"

Navarro et al., 2006x

**Reason for exclusion**	Mixed aquatic interventions, no conclusions can be drawn to WATSU. For details see "Characteristics of studies awaiting classification"

Navarro et al., 2006xa

**Reason for exclusion**	Mixed aquatic interventions, no conclusions can be drawn to WATSU. For details see "Characteristics of studies awaiting classification"

Nogueira et al., 2017x

**Reason for exclusion**	Mixed aquatic interventions, no conclusions can be drawn to WATSU. For details see "Characteristics of studies awaiting classification"

Oh et al., 2015x

**Reason for exclusion**	Mixed aquatic interventions, no conclusions can be drawn to WATSU. For details see "Characteristics of studies awaiting classification"

Orsini et al., 2009x

**Reason for exclusion**	Mixed aquatic interventions, no conclusions can be drawn to WATSU. For details see "Characteristics of studies awaiting classification"

Park et al., 2015x

**Reason for exclusion**	Mixed aquatic interventions, no conclusions can be drawn to WATSU. For details see "Characteristics of studies awaiting classification"

Park et al., 2016x

Reason for exclusion	Mixed aquatic interventions, no conclusions can be drawn to WATSU. For details see "Characteristics of studies awaiting classification"

Pattman et al., 2013x

Reason for exclusion	Mixed aquatic interventions, no conclusions can be drawn to WATSU. For details see "Characteristics of studies awaiting classification"

Romeiro & Navarro, 2011x

Reason for exclusion	Mixed aquatic interventions, no conclusions can be drawn to WATSU. For details see "Characteristics of studies awaiting classification"

Santana et al., 2005x

Reason for exclusion	Mixed aquatic interventions, no conclusions can be drawn to WATSU. For details see "Characteristics of studies awaiting classification"

Santos & Facci, 2009x

Reason for exclusion	Mixed aquatic interventions, no conclusions can be drawn to WATSU. For details see "Characteristics of studies awaiting classification"

Santos et al., 2018x

Reason for exclusion	Mixed aquatic interventions, no conclusions can be drawn to WATSU. For details see "Characteristics of studies awaiting classification"

Silva & Navarro, 2006x

Reason for exclusion	Mixed aquatic interventions, no conclusions can be drawn to WATSU. For details see "Characteristics of studies awaiting classification"

Silva et al., 2006x

Reason for exclusion	Mixed aquatic interventions, no conclusions can be drawn to WATSU. For details see "Characteristics of studies awaiting classification"

Smeeding & Osguthorpe, 2005x

Reason for exclusion	Mixed interventions, no conclusions can be drawn to WATSU. For details see "Characteristics of studies awaiting classification"

Smeeding et al., 2010x

Reason for exclusion	Mixed interventions, no conclusions can be drawn to WATSU. For details see "Characteristics of studies awaiting classification"

Smeeding et al., 2011x

**Reason for exclusion**	Mixed interventions, no conclusions can be drawn to WATSU. For details see "Characteristics of studies awaiting classification"

Taketa et al., 2018x

**Reason for exclusion**	Mixed aquatic interventions, no conclusions can be drawn to WATSU. For details see "Characteristics of studies awaiting classification"

Tanoue et al., 2009x

**Reason for exclusion**	Mixed aquatic interventions, no conclusions can be drawn to WATSU. For details see "Characteristics of studies awaiting classification"

Tonieto et al., 2015x

**Reason for exclusion**	Mixed aquatic interventions, no conclusions can be drawn to WATSU. For details see "Characteristics of studies awaiting classification"

Tüfekçioğlu 2009x

**Reason for exclusion**	Only one position of WATSU, no conclusions can be drawn to WATSU. For details see "Characteristics of studies awaiting classification"

Useros-Olmo & Collado-Vázquez, 2010x

**Reason for exclusion**	Mixed aquatic interventions, no conclusions can be drawn to WATSU. For details see "Characteristics of studies awaiting classification"

Useros-Olmo et al., 2018x

**Reason for exclusion**	Mixed interventions, no conclusions can be drawn to WATSU. For details see "Characteristics of studies awaiting classification"

Vogtle et al., 1998x

**Reason for exclusion**	Mixed aquatic interventions, no conclusions can be drawn to WATSU. For details see "Characteristics of studies awaiting classification"

Zanella 2011x

**Reason for exclusion**	Mixed aquatic interventions, no conclusions can be drawn to WATSU. For details see "Characteristics of studies awaiting classification"

Footnotes

Characteristics of studies awaiting classification

Araújo & Assis, 2018

**Methods**	Case series
**Participants**	2 patients diagnosed with Amyotrophic Lateral Sclerosis
**Interventions**	The hydrokinesiotherapy program lasted 16 weeks, with 45-minute sessions twice a week in a heated pool at 33ºC.<br>Twenty aquatic physiotherapy sections were performed, in the first instance the appropriate orientations were made and then the exercises were applied, observing each movement and energy expenditure of this patient closely, the following exercises were performed:<br>Stretching was done using passive physical force of water and actively stretching high by taking the lower limbs, upper and lower limbs. In the first three sections, the patient's adaptation to the swimming pool, space recognition and breathing exercises were performed through circle gait, adduction and abduction against water force, step balance and relaxation exercises using Watsu techniques.
**Outcomes**	ALS Functional Rating Scale
**Notes**	Mixed aquatic interventions, no conclusions can be drawn to WATSU.

Becker & Lynch, 2017

**Methods**	Case report
**Participants**	54 year old female, end stage of dementia, non-verbal, seated in a wheelchair, dependent on 2-person assist for all transfers and activities of daily living (ADLs.) She had been either non-responsive or actively resistive for both ADLs and transfers in the 6 months prior to assessment.
**Interventions**	17 one hour therapy sessions over 19 weeks in warm water therapy pool
**Outcomes**	ability to tread water for 15 minutes, transfers improved to moderate to-maximum assist from seated, ambulation improved to 1000’ with minimum-to-moderate assist of 2 persons. Communication increased to appropriate “yes,” “no,” and “OK” appropriate responses, occasional “thank you” and “very nice.”
**Notes**	WATSU was applied in the beginning of the intervention, temperature 89-90°F

Bonetti et al., 2010

**Methods**	Case series
**Participants**	480 participants, patients with dislipidemia, cardiovascular problems and asymtomatic patients but with at least one risk factor for coronary disease, age 20- 89, 30 of them had WATSU
**Interventions**	aquatic therapy was group therapy on ongoing basis (WATSU was combined with halliwick and biodanza)
**Outcomes**	primarily leveraged structured questionnaires, verbal reports, and clinical and biochemical analysis. results reported narratively only
**Notes**	temperature not reported, likely 30°C as in Cantos 2008 and 2008a

Cantos & Souza, 2013

**Methods**	Case series
**Participants**	2 patients with diabetes
**Interventions**	Watsu, Halliwick and Biodanza. Unclear if techniques were mixed in one session or in succession, probably WATSU in groups
**Outcomes**	Laboratory assessment (GLI, CT, TG, HDL-c, LDL-c, URCA,CREA,HbA1c), clinical and nutritional care, the emotional state of patients.<br>both patients had individual assessments before and after (in one patient:) LIPP-Scale, SIMON-Scale, NAHAS-Scale,ALVES-Scale; all scales for stress-levels<br>The evaluation of the physical condition was performed with Boucher questionnaire<br>Cohen Perceived Stress Scale
**Notes**	Temperature: "adequate"

Cantos et al., 2008

**Methods**	Case series
**Participants**	20, 50-70 years, psychological stress in chronic deseases: anxiety, fatigue, palpitation, sweating, exaggerated feelings of nervousness and frightenings, cold hands, in the one in the stomach, change of appetite, sleeplessness, apathy, nausea, giddiness, to think about one alone subject and extreme irritability. These symptoms had improvements in 30% of the cases told throughout the therapy. The state of stress was evaluated by a second questionnaire according Lipp (1996). For the answers pointed before and after the treatment an improvement in 70% of the participants, whose more representative elements could have been noticed by the control biggest of the memory, for the good disposal and reinvigoration to if raising, for the fall of anxiety and minor was perceived fear of the loss control, of the acts, words, thoughts and feelings.<br>fibromyalgia, osteoarthritis, cardiovascular problems, diabetes, Dyslipidemias, labyrinthitis, in addition to bone surgeries.
**Interventions**	WATSU, Biodanza, Halliwick IN GROUP-SETTING! weekly, 12 months
**Outcomes**	The symptoms more observed by the answers of the questionnaire elaborated for Lipp (1999) in the related group had been: anxiety, fatigue, palpitation, sweating, exaggerated feelings of nervousness and frightenings, cold hands, in the one in the stomach, change of appetite, sleeplessness, apathy, nausea, giddiness, to think about one alone subject and extreme irritability. These symptoms had improvements in 30% of the cases told throughout the therapy. The state of stress was evaluated by a second questionnaire according Lipp (1996). For the answers pointed before and after the treatment an improvement in 70% of the participants, whose more representative elements could have been noticed by the control biggest of the memory, for the good disposal and reinvigoration to if raising, for the fall of anxiety and minor was perceived fear of the loss control, of the acts, words, thoughts and feelings.
**Notes**	30° C

Cantos et al., 2008a

**Methods**	Case series
**Participants**	10
**Interventions**	WATSU, Biodanza, Halliwick in group setting
**Outcomes**	Lipp Distress Scale, Cholesterol (LDL, HDL, Trigyceride) laboratory (reported)
**Notes**	30°C

Cantos et al., 2013a

**Methods**	Case series: report of program
**Participants**	22 cardiovascular
**Interventions**	One weekly aquatic group therapy session is provided by the program 2011. Group therapy with Watsu and halliwick? Aquatic therapy named as "Watsu/Halliwick" - The Biodanza-program
**Outcomes**	narrative
**Notes**	likely 30°C as in Cantos 2008 and 2008a

Chang et al., 2009

**Methods**	Case series
**Participants**	female hemiplegic patients
**Interventions**	aquatic exercise program 16 weeks
**Outcomes**	the Motor Assessment Scale (MAS)
**Notes**	not clear if Watsu was part of program in each session or as session as whole<br>Temperature: ?

Costa et al., 2017

**Methods**	RCT
**Participants**	10 stroke survivors, >25 years (mean 56), 3 male and 7 female, mini-mental-scores >10; ≥ 51 in Fugl-Meyer functionality score; stroke mean 30 month ago, two groups (water and floor)
**Interventions**	Intervention group: 10 times aquatic therapy of which 10 minutes were WATSU.
**Outcomes**	6 min walking test: After intervention significant increase in the number of turns [12.80 ± 2.78 (p = 0.01)] in both groups;<br>stroke specific quality of life scale (SS-QOL): [196.20 ± 42.92 (p = 0.046)] only significant change after hydrotherapy protocol, when compared to the baseline condition.
**Notes**	Temperature: ?

Degani & Villa, 2005

**Methods**	Case study
**Participants**	1 patient with juvenile idiopathic arthritis
**Interventions**	Intervention was a mixture of techniques of Bad Ragaz, Halliwick, Aquastretching and Watsu. 12 months (3 times a week)
**Outcomes**	The results indicated an increased ROM<br>in almost all joints, and an improvement in HRQL, including functional capacity, physical aspect, pain, general health, vitality, social function, and mental health. As just aquatic physical therapy was applied during this period of 12 months beside drug therapy, it can be concluded that increased ROM and improved HRQL, such as reduced pain and decreased difficulty experienced in performing daily tasks, were due to the aquatic therapy program.
**Notes**	Temperature: 33 ºC

Elsner et al., 2009

**Methods**	Case series
**Participants**	3 patients with mastectomy
**Interventions**	Combined techniques in 60 min. group sessions, 10 sessions total (3 weekly sessions). The final 10 minutes were intended for relaxation, where uses Watsu techniques.All meetings were held in groups.
**Outcomes**	SF-36
**Notes**	Temperature:33 - 35°C

Eo & Lee, 2011

**Methods**	CT
**Participants**	pregnant women (N=20, Aquatic exercises n=10, control group: n=10)
**Interventions**	Watsu amongst exercises, actually a fitness-program
**Outcomes**	Fitness factors were measured functional reach test(FRT), balance and endurance in respective group by water exercise. Investigation of delivery used questionnaire after childbirth. FRT of the exercise group was significantly increased but not in control group($p<.05$). Pregnant woman’s weight was significantly increased in respective group($p<.01$). Birth weight of fetal and time of delivery was decresed in exercise group($p<.05$).
**Notes**	31°C

Ferreira & Matsutani, 2006

**Methods**	Case series
**Participants**	8 patients with fibromyalgia

**Interventions**	"10 individual sessions of exercises of relaxation application of the method Bad Ragaz and Watsu, which consists of a relaxation technique in the water through passive movement, led by physiotherapist" Mixture of Bad Ragaz and Watsu, however, relaxation techniques only. "In the second session, the patient started hydrotherapy. The purpose of these first sessions was to perform aquatic adaptation that consisted of immersion exercises, breathing control, flotation, walking, besides the use of floats: neck float, pelvic float and ankle floats of the brand Floty®. During the 4th session until the 9th, some adaptation exercises were performed, previously described and then the Bad Ragaz and Watsu method." WATSU seems to have been applied, yet it is totally unclear to what extent, whether in every session or only once in a while for a entire session etc. No conclusions can be drawn.
**Outcomes**	pain for Visual Analogical Scale (VAS)<br>painful tender points at palpation<br>Fibromyalgia Impact Questionnaire (FIQ)<br>flexibility for the test of in the distance of the third finger-ground<br>Only 6 patients analysed, 2 Drop outs
**Notes**	Temperature: 33º - 35ºC

Gimenes et al., 2007

**Methods**	Case series
**Participants**	14 patients with herniated disc
**Interventions**	The hydrokinesiotherapy protocol was applied twice a week, lasting 40 minutes each session, for 12 weeks.<br>Aquatic activities involved 5 minutes of warm-up with gait associated with changes of direction, 25 minutes of strengthening the muscles of the trunk and lower limbs, and 10 minutes of muscle relaxation with ball massage in the region of greatest referred pain, passive stretching of the trunk and lower limbs muscles or application of the Watsu relaxation method.<br>The equipment used was aquatic floats of different densities; rubber balls, stereo, cd's with songs of different rhythms; in a semi-enclosed pool with 7x8m side bars, 1.40m deep
**Outcomes**	Numeric rating scale pain: $p = 0.0157$; numeric rating scale Quality of Life: $p = 0.3305$
**Notes**	water temperature ranging from 32ºC to 34ºC. Same project as Gimenes et al., 2008

Gimenes et al., 2008

**Methods**	Case series
**Participants**	20 patients with more than 60 years of age, both male and female, without cognitive compromising and that did not practice regular physical activity for a year
**Interventions**	The program of aquatic physiotherapy (group therapy) was applied two times a week, in 45- minute sessions, during 12 weeks, consisting of 10 minutes of heating, 20 minutes of aerobic exercises and relaxation in the 15 final minutes.

**Outcomes**	Systolic Arterial Pressure (SAP) and Diastolic Arterial Pressure (DAP)
**Notes**	Intervention was group therapy (10 patients), watsu was only part of relaxation "relaxation in the final 15 minutes with Watsu method and / or Tai-Chi"<br>Temperature: not mentioned

Gimenes et al., 2008a

**Methods**	Case series
**Participants**	14 patients, 30-60 yrs, medical diagnosis of lumbar disc herniation (>1 yr), do not engage in any other physical activity (except aquatic physical therapy)
**Interventions**	The hydrokinesiotherapy protocol was applied twice a week, lasting 40 minutes each session, for 12 weeks. This protocol consisted of performing aquatic activities that involved 5 minutes of warm-up with gait associated with changes of direction, 25 minutes of strengthening the muscles of the trunk and lower limbs, and 10 minutes of muscle relaxation with ball massage in the region of greatest referred pain. in the day, passive stretching of the trunk and lower limbs muscles or application of the Watsu relaxation method.<br>The equipment used was aquatic floats of different densities; rubber balls, stereo, cd's with songs of different rhythms; in a semi-enclosed pool with 7x8m side bars, 1.40m deep, water temperature ranging from 32ºC to 34ºC.
**Outcomes**	The evaluations were performed on the first and last day of activities, by therapists blind to the study objectives, properly trained to apply both therapies and questionnaires.<br>Pain assessment was performed using the Visual Numerical Scale, Life satisfaction was made through a growing chart in vertical columns numbered from 1 to 10 in which the patient indicated the value equivalent to his life satisfaction, being 1 little satisfied and 10 very satisfied with life.<br>The t - Student test was adopted for the analysis and there were statistically significant improvements in the pain variable and a clinical improvement in the life satisfaction, despite the lack of statistical significance.<br>Pre X Post P Statistical Significance p <0.05<br>pain 0.0157 Yes<br>life satisfaction 0.3305 No<br>p-Value = measures the amount of evidence the sample provides
**Notes**	only p-values reported

Gonçalves et al., 2009

**Methods**	Case report
**Participants**	female, 35 years; onset of pain 3 years ago, three surgeries on thoracic outlet: twice to remove the cervical rib and once to remove fibrosis caused by the first surgical procedure. limitation of movements of upper limbs, neck, head, hip, pain in the chest region, left upper limb and trapezius region, muscular tension in shoulder and neck region and difficulties activities of daily living.
**Interventions**	mixed program including WATSU, 22 times (each 45 minutes) in 11 weeks

**Outcomes**	VAS<br>"In baseline gait evaluation, the patient lacked several important components for adequate ambulation; after the treatment, the patient presented upper limb oscillation, knee and hip flexion, and dissociation of scapular and pelvic girdle.. In the initial evaluation of the analogue pain scale, where 0 is no pain at all and 10 is maximum pain, the patient presented 8, and after treatment this degree of pain became 3, demonstrating how effective the therapy was in the pain and overall picture of this patient."<br>Increased range of cervical motion (goniometry):<br>Rotação pre 09° / 09° (right / left) post 10° 12°<br>Inclinação pre 12° 10º post 22° 22°<br>Flexão pre 10° 10° post 22° 22°<br>Extensão pre 09° 09° post 21° 21°<br>Increased strength (Oxford scale):<br>Bíceps pre 3 / 2 (right / left) post 4 4+<br>Tríceps pre 3 2 post 5 4<br>Deltóide pre 4 3 post 5 4
**Notes**	Temperature: ?

Jacintho et al., 2008

**Methods**	Case series
**Participants**	10 patients, 35-67 years, 2 dropouts not analyzed, Fibromyalgia
**Interventions**	not clear, whether WATSU was part of the protocol at all; 16 weeks "Each session consisted of 10 minutes of stretching, 40 minutes of aerobic training according to the desired intensity, followed by 10 minutes of relaxation."
**Outcomes**	After the intervention, an improvement on the SF-36's general average, as well as on the average of all controls of the mental and physical components, was observed.
**Notes**	28-31 °C, active exercises

Jakaitis & Guazelli, 2005

**Methods**	Case report
**Participants**	1, Vigil coma patient: 40 years, one year in coma. cognitive level II (Rancho Los Amigos scale) 8, after one year of hospitalization. Clinically stabilized in the physiotherapeutic evaluation, it presents global spasticity level III (Ashworth scale) 17, non selective movement of upper and lower limbs with predominance of the right hemicorp, cervical and trunk control in the static and dynamic evaluation are precarious and presenting hyperreflexia And involuntary movement of the left lower limb. It presents spontaneous eye opening and does not do directed visual monitoring.
**Interventions**	"The study was conducted within 18 months, three weekly sessions lasting 45 minutes." "After a multidisciplinary evaluation, aquatic physiotherapy was started, in which its protocol was based on vestibular stimulation. It consisted of hearing immersion, tonic adjustment, global relaxations (displacements and fluctuations), stimulation of the posture stages (passive three-dimensional mobilizations), cognitive stimulation and environmental suitability. The aquatic treatment was

	based on the Halliwick, Bad Ragaz and Watsu methods, specific to aquatic physiotherapy, but adapted from terrestrial methods such as Bobath, Kabat, RPG and Shiatsu, for therapeutic purposes."
**Outcomes**	An initial evaluation was made using the Rancho Los Amigos and Ashworth scales for statistical measurement, reassessed monthly, maintaining the same treatment protocol and not performing terrestrial therapy.<br>In the sensory context, we observed the improvement of spatial and cognitive awareness in the present study, through the analysis of the initial (II) and final (IV) scores of the Rancho Los Amigos scale, being elevated.<br>Analyzing the motor acquisitions, we can see improvement of the cervical control, adjustment and tonic adequacy, improvement of the postural pattern and reduction of the reflex of the left lower limb.<br>No significant improvement in the Ashworth scale (always III), however, no shortening and deformity facilities were developed, representing a very important focus for maintaining the integrity of joint motion amplitudes.
**Notes**	Different methods mixed in water treatment, WATSU considering the state of the patient likely to be central.<br>Temperature: not reported

Kakihara & Neves, 2005

**Methods**	CT
**Participants**	11 patients diagnosed with ischemic or hemorrhagic stroke, male and female, aged between 53 and 81 years, all underwent soil physical therapy, only 5 of them underwent hydrotherapy in combination with soil therapy. Ground 2-3 times weekly and in the water once a week. Duration: 2 months
**Interventions**	Watsu, Halliwick and Bad Ragaz
**Outcomes**	"Although the study did not present any statistically significant difference among the variables, it was found that some variables improved after 2 months of physiotherapy, both in the group where the patients only received ground therapy and in the group in which the patients were submitted. to ground therapy plus hydrotherapy. Patients who did not undergo hydrotherapy showed an increase in the means of the variables: evacuation, urination, chair / bed, stairs, bath and in the total variable. Patients who underwent hydrotherapy associated with ground therapy showed improvement in the means of the variables: neatness, urination, dressing, mobility, stairs and the total variable. If we compare the differences in the averages of the total variable between October and December between the patients who did not hydrotherapy and those who did hydrotherapy, we notice that there was a variation of 4.1 in patients who did not hydrotherapy, while in patients who did hydrotherapy. , this variation was greater: 8.0. This suggests that patients who associate soil therapy with hydrotherapy are more likely to improve ADLs than those who only perform soil therapy. This work demonstrates that even in a short time of 2 months, patients who have suffered a stroke may find it easier to perform their daily activities due to physical therapy, even if their complete rehabilitation is only achieved. after years of physical therapy treatment."
**Notes**	40 min WATSU

Kim & Lee, 2015

**Methods**	CT
**Participants**	20 children with cerebral palsy, 2 groups (10/10)
**Interventions**	30min WATSU in mixed program, 2 times per week, total 6 weeks: "Aquatic exercise treatment group (experimental group) The aquatic exercise therapy applied to the study was performed by using a buoyancy device to completely float the subject's body Based on Watsu, which is a traditional aquatic exercise method, the 5th-year therapist who has completed Watsu 1, 2, 3 specialist training of the international WABA Association in a way to perform therapeutic intervention with minimized weight load on the lower limb (Table 2). The results of the study were as follows. The buoyancy devices used in the study were arm rings worn on both proximal arms. Intervention was performed for 30 minutes for each session, twice a week for a total of 6 weeks. The first step is to establish a relationship with the therapist,<br>Maintaining contact and allowing the child to become accustomed to the water using light levels of shaking. Because fear increases muscle strain, creates abnormal movements, and interferes with the learning of motor control (Mary & Diane, 1998), it is aimed at eliminating it.<br>Step 2 is the stage where the child is assisted by the therapist before wearing the floating device.<br>The basic procedure is the same as in Step 1, but the therapist gradually moves to the side or back to reduce assistance and move out of the child's field of vision. In this step, the child is free from the fear of movement in the water, can swim with his hand, rolls his or her feet, and learns to move in the water.<br>In phase 3, the patient is assisted by a buoyant device. This is done in a way that reduces the therapist's assistance and provides a static load by maintaining an upright posture against water flow and buoyancy. This is the same principle as maintaining the posture on the shaking plate, which can improve the balance ability and stability restoration ability of the object (Anne et al., 2003).<br>In the fourth stage, the subject starts to actively move while wearing a buoyancy mechanism, and learns the separated movements of the pelvis and the lower limb. Reduced tension allows the movement of each joint to be performed smoothly, allowing the user to experience the exercise pattern of the lower limb necessary for walking."
**Outcomes**	Kobayashi-Frostig MSTB made with reference to MOB (Movement Skill Test Battery)
**Notes**	temperature?, several therapies involved, mainly active intervention

Kim et al., 2009

**Methods**	CT
**Participants**	12 children (9 male, 3 female) with cerebral palsy (6 intervention, 6 control). Able to walk, mean age 10 years
**Interventions**	20 min WATSU in mixed program of total 60 minutes duration

**Outcomes**	Kobayashi-Frostig with reference to MOB (Movement Skill Test Battery, MSTB) "Aquatic rehabilitation exercise showed effect on sitting trunk flection of children with hemiplegia.<br>Aquatic rehabilitation exercise showed effect on standing long jump, shuttle run change of body position, sit-up of children with hemiplegia.<br>Aquatic rehabilitation exercise showed effect on opened-eyes foot balance, closed- eyes foot balance of children with hemiplegia.<br>Aquatic rehabilitation exercise showed effect on movement skill test battery`s total score of children with hemiplegia.<br>In conclusion, aquatic rehabilitation exercise has a positive effect on the body flexibility, muscle strength, agility and balance which is contribute to improve of physical fitness and health. Therefore, further studies were suggested that various and specific measures for the practical application of this program were to be applied."
**Notes**	32 °C

Kwangmin et al., 2016

**Methods**	CT
**Participants**	32 patients 8-48 years (18 male, 14 female) cerebral palsy. Their cerebral palsy levels ranged from 1 to 3. 3 groups, n=10 in Aquatic group
**Interventions**	assisted aquatic movement, horseback riding therapies, and controls "The intervention for the AATG consisted of warming up for 10 min (i.e., dorsiflexion, plantar flexion, lateral bending right and left, lifting the legs toward the head while floating on the water, pronation, and supination), assisted aquatic movement therapy for 30 min (i.e., floating on the water using buoyancy, floating on the water in a sitting posture and walking backward and forward, walking right and left backward and forward, on the pool bottom in the water while wearing a weight suit, putting numbers on a wall using a buoyancy ring, and playing ball-catching and throwing game with instructor), and cooling down for 10 min (i.e., cooldown with Watsu basic movement exercise, one and two leg offering, accordion, rotating accordion, near and far leg rotation, over grip rotation, stillness, and follow movement)"
**Outcomes**	Electroencephalograms, the Feeling Scale and the Felt Arousal Scale were examined as dependent variables.<br>Analysis of self-reported data demonstrated a significant positive improvement in the emotions of participants in the assisted aquatic movement therapy group in comparison with the control group. With regard to the electroencephalogram analysis, the results of this study showed increased alpha power in the assisted aquatic movement therapy group compared with the horseback riding and control groups.
**Notes**	only 10 min cooling down with WATSU

Lee & Kim, 2015

**Methods**	Case series
**Participants**	10 children with encephalopathy
**Interventions**	Water rehabilitation exercises
**Outcomes**	EEG Hemispheric Asymmetry, emotion questionnaires
**Notes**	Watsu only part of Cool Down part of therapy session<br>Temperature: 34±1°C

Lotan & Barmatz, 2009

**Methods**	Case report
**Participants**	1 patient with Rett Syndrome (RS)
**Interventions**	Hydrotherapy during 3 years and also conventional physical therapy, hippotherapy.
**Outcomes**	learned to improve her communication abilities by communicating her wishes (through eye pointing) to the therapist,thereby achieving a sense of control over the situation. Her ability to control her body gradually improved, and she was able to move more freely in the water, thereby achieving functional abilities such as climbing a floatation device and moving her body in the water. Her ability to walk in the water was transferred to other areas and during that time and onward, she has also shown the ability to take some steps (supported at shoulder level) on land, as well as to go up and down aflight of stairs (fully supported)
**Notes**	"aquatic environment is highly recommended for the person with RS", not clear in which way (and whether at all) WATSU was included in hydrotherapy<br>Temperature: "warm"

Maczkowiak et al., 2007

**Methods**	RCT
**Participants**	31 Patients (m = 11, f = 20, mean age 45 years) diagnosed with depression ICD-10 (F32.00 or F33.00), Depression index: >18, mean 25 (BDI).<br>WATSU-group: m = 2, f = 7.
**Interventions**	3 groups:<br>Group 1:exercis-/ experience-centered (z.B. Walking, Jogging, Aqua Jogging, Yoga, spinal exercises)<br>Group 2: experience- / conflict-centered I (z.B. Tanztherapie, Integrative Motion Therapy, Walking, Jogging)<br>Group 3: experience- / conflict-centered II WATSU (once administered by a therapist, 9 times by peers), Walking, spinal exercises, Aquatraining
**Outcomes**	depression, anxiety, mental health:<br>Beck-Depression-Inventory (BDI),<br>Frankfurter Körperkonzeptskalen (FKKS)<br>Sense of Coherence (SOC)<br>Fragebogen zum Körperbild (FKB 20), to assess body image disturbances<br>Mindful Attention Awareness Scale (MAAS), awareness and attention<br>Erfassung des „Spielerischen Welterlebens“ nach Ebinghaus und Dillmann (2005)<br>Genuss-Fragebogen Frankes

pre: two weeks before treatment (at home), post: at the end of stationary treatment.
BDI, t1 t2
Gruppen N M SD M SD /Effekte F sign. Eta-Quadrat
1 12 27,50 7,98 21,92 9,54 /Zeit 43,944 0,000 0,611
2 10 22,70 10,76 12,70 10,48 /Gruppen 5,747 0,008 0,291
3 9 24,67 5,32 5,00 3,24 /Interaktion 5,403 0,010 0,278
ges. 31 25,13 8,36 14,03 10,91

SOC, t1 t2
Gruppen N M SD M SD /Effekte F sign. Eta-Quadrat
1 12 104,83 18,44 105,25 22,92 /Zeit 6,610 0,016 0,191
2 10 113,80 34,68 132,00 31,26 /Gruppen 3,900 0,032 0,218
3 9 113,89 20,76 141,11 23,58 /Interaktion 1,836 0,178 0,116
ges. 31 110,35 24,91 124,29 29,73

FKKS– Selbstakzeptanz, t2 t2
Gruppen N M SD M SD Effekte F sign. Eta-Quadrat
1 12 3,08 0,83 3,06 0,67 Zeit 9,030 0,006 0,244
2 10 2,80 0,79 3,30 0,86 Gruppen 1,051 0,363 0,070
3 9 2,83 0,77 3,97 0,71 Interaktion 3,514 0,043 0,201
ges. 31 2,92 0,79 3,40 0,82

FKKS– Akzeptanz durch andere, t2 t2
Gruppen N M SD M SD Effekte F sign. Eta-Quadrat
1 12 3,68 0,79 3,58 0,99 Zeit 2,819 0,104 0,091
2 10 3,98 1,04 3,95 0,85 Gruppen 1,093 0,349 0,072
3 9 3,76 0,84 4,59 1,10 Interaktion 4,274 0,024 0,234
ges. 31 3,80 0,87 3,99 1,04

FKB20 – Vitalität, t1 t2
Gruppen N M SD M SD Effekte F sign. Eta-Quadrat
1 12 1,89 0,65 2,10 0,72 Zeit 19,155 0,000 0,406
2 10 2,15 0,78 2,94 0,91 Gruppen 7,077 0,003 0,336
3 9 2,18 0,44 3,51 0,73 Interaktion 3,397 0,048 0,195
ges. 31 2,06 0,64 2,78 0,97

FKB20 – Ablehnende Körperbewertung, t1 t2
Gruppen N M SD M SD Effekte F sign. Eta-Quadrat
1 12 2,88 0,76 2,55 0,88 Zeit 14,289 0,001 0,338
2 10 2,66 0,97 2,26 0,74 Gruppen 2,011 0,153 0,126
3 9 2,40 0,69 1,80 0,36 Interaktion 0,452 0,641 0,031
ges. 31 2,67 0,82 2,24 0,76

MAAS, t1 t2
Gruppen N M SD M SD Effekte F sign. Eta-Quadrat
1 12 3,69 0,70 3,44 0,81 Zeit 4,990 0,034 0,151
2 10 3,24 0,79 3,90 0,95 Gruppen 1,082 0,353 0,072
3 9 3,61 0,81 4,28 0,62 Interaktion 3,858 0,033 0,216
ges. 31 3,52 0,77 3,83 0,86

	Spielerisches Welterleben, t1 t2<br>Gruppen N M SD M SD Effekte F sign. Eta-Quadrat<br>1 12 4,82 1,17 4,87 1,27 Zeit 8,559 0,007 0,241<br>2 10 4,63 1,01 5,20 1,01 Gruppen 0,215 0,808 0,016<br>3 9 4,28 1,03 5,90 1,02 Interaktion 3,371 0,049 0,200<br>ges. 31 4,60 1,07 5,28 1,17<br><br>Genuss, t1 t2<br>Gruppen N M SD M SD Effekte F sign. Eta-Quadrat<br>1 12 2,40 0,42 2,58 0,43 Zeit 11,549 0,002 0,292<br>2 10 2,28 0,68 2,83 0,70 Gruppen 0,110 0,896 0,008<br>3 9 2,15 0,49 2,85 0,38 Interaktion 1,256 0,300 0,082<br>ges. 31 2,29 0,53 2,74 0,52
**Notes**	Temperature:35 °C, duration not reported. WATSU was received by the patient once and then exchaged (given and received) with the other patients nine times. Unclear, whether giving or receiving WATSU is responsible for the enormous effect on depression.

Martins et al., 2015

**Methods**	Case series
**Participants**	8 children with cerebral palsy classified at levels I, II and III of Gross Motor Function Classification System (GMFCS)
**Interventions**	nine months of intervention with neuroevolutive Bobath concept, aquatic therapy and virtual rehabilitation
**Outcomes**	GMFM (Gross motor function measure)
**Notes**	Aquatic therapy was a mixture of Bad Ragaz, Halliwick and Watsu<br>Temperature: not mentioned ("effects of water therapy treatments depend on temperature...")

Masselli et al., 2009

**Methods**	Case series
**Participants**	10 work related musculoskeletal disorder (WRMD) patients
**Interventions**	The aim of this study was to propose a program aquatic exercise and analyze its benefits by investigating the improvement of symptoms and the functional capacity of patients undergoing hydrotherapy.<br>10 sessions of hydroherapy including walking in water, streching, strenghtening and last relaxation in the supine 2007
**Outcomes**	Boston-Questionnaire
**Notes**	Not clear which methods were used, if Watsu was used at all<br>Temperature: not mentioned

Mazetto & Navarro, 2007

**Methods**	Case report
**Participants**	male, 40 years, rheumatoid arthritis. Initial evaluation: deformities in upper limbs in semiflexion; with reduced range of motion (ROM) in the hip, knees, wrists, elbows and shoulders. Patient wanders and is semi-dependent on their daily living activities (ADL), needing help with foot hygiene and wearing socks and shoes. It presents balance deficits with compromised gait. He started Orthopedic Physiotherapy four months ago.
**Interventions**	"To finish Watsu for ten minutes, using methods such as breathing balance, accordion, rotational accordion, proximal leg rotation, distal leg rotation and sea kelp." 5 sessions of 45 minutes duration. The treatment goals were to improve gait, improve static and dynamic balance, improve upper limb motor coordination and lower limbs, provide better quality of life, promote muscle relaxation and ADM gain in MMSS, MMII and prevent further deformities and promote analgesia.
**Outcomes**	"We observe significant improvement in the range of motion in the joints, balance and functionality."<br><br>Shoulder pre flexion 155 140 (right / left) extension 30 50 (right / left) post 155 150 45 50<br>Elbow pre 90 70 80 45 post 90 85 60 45<br>Knee pre 80 80 0 0 post 80 85 0 0<br>Trunc pre 70 10 post 70 10
**Notes**	Temperature ?

Melo et al., 2012

**Methods**	Case study
**Participants**	child with clinical diagnosis of hydrocephalus and physical therapy diagnosis of moderate spastic quadriplegia
**Interventions**	20 sessions of hydrotherapy, three times per week, lasting 45 minutes<br>The patient was taken to the pool by the physiotherapist in ball position. Watsu was used for adjustment to water and muscle relaxation.<br>Adaptations: Patient sitting between the legs of the physiotherapist who performed pressure tapping on the shoulder with a focus on shoulder external rotation, extending the hand and thus extending the arm.<br>Again WATSU maneuvers at the end of each session, leave the child calm, quiet, relaxed and with greater range without spasm muscle before entering the pool.
**Outcomes**	Hydrotherapeutic and Neuropediatric Card used in the school clinic for evaluation in the supervised stage<br>CHQ-PF50<br>Modified Ashword Scale: Alteração de tônus muscular antes e após sessão de hidroterapia através da Escala Modificada de Ashwoorth: From the second week until the end of treatment, the patient was more adapted to the aquatic environment, presenting improvement of a point related to muscular tonus.<br>1º semana 3 3<br>2º semana 3 2<br>3º semana 3 2

	4º semana 3 2<br>5º semana 3 2<br>6º semana 3 2<br>7º semana 3 2<br>With reference to the children's health questionnaire - CHQ-PF50 Parents' Report, results were obtained in the following items: body pain, emotional impact on parents and family activity. About body pain over the last four weeks was classified as "very often" moderately prior to treatment and "mild and with some frequency" after treatment. The emotional impact on the parents was "enough" before treatment for and "some" after treatment, the mother reported much emotional damage and fear regarding the physical health of the child. In the question of family activity, it was the answer "sometimes" the patient's health limited the family to leave home having to cancel and change plans in the last hour to "almost never".<br>The remaining items of the questionnaire remained unchanged after the hydrotherapy sessions, according to a questionnaire answered by the mother. before entering the pool the child presented with a number of three muscle spasms per minute<br>And after the ducts these spasms were spreading from five about ten minutes with a single episode of muscle spasm being noticed.
**Notes**	Temperature: 32 to 33 °C

Mesquita et al., 2007

**Methods**	Case series
**Participants**	8 military policemen between 25 and 45 years old, with idiopathic low back pain
**Interventions**	ten sessions of hydrotherapy, twice a week: exercise program, with 50 minutes each session, was as follows: 5 minutes of walking, 40 minutes of specific exercises – float suspension bicycle in the axillar area and 5 minutes of stretching of paravertebral, biceps femuralis, gluteus maximus and medius, tensor faciae latae muscle, lumbar quadrate and triceps surae. At the end of each session, 5 minutes of relaxation are performed with classic Watsu maneuvers.
**Outcomes**	"One concludes that the stretching method used by hydrotherapy in patients with low back pain promoted reduction in HP levels and low back pain. "
**Notes**	34° C, 5 min WATSU at the end of active program

Nascimento et al., 2012

**Methods**	Case report
**Participants**	1 Patient with Guillain-barré
**Interventions**	Treatment methods used were:hydrokinesiotherapy by the methods, Watsu and Bad Ragaz,divided according to the needs and phase treatment.
**Outcomes**	SF-36, manovacuometer and goniometria
**Notes**	Watsu only part of each treatment (mostly cool down, relaxation)<br>Temperature:33°C

Navarro et al., 2006

**Methods**	Case series
**Participants**	Two female patients with Fibromyalgia (39 and 43 years), > 15 years of pain
**Interventions**	12 water therapy sessions per patient in 4 weeks. The hydrotherapy session was one hour, where ten minutes were performed warm-up exercises ten minutes of stretching, dynamic ten minutes and thirty minute adapted Watsu, because the patients needed to wear the neck vest.
**Outcomes**	Whoquol-bref intitial and at end of treatment<br>VAS pain at each session
**Notes**	Temperature: 34º C a 35º C

Navarro et al., 2006a

**Methods**	Case series
**Participants**	two female patients
**Interventions**	12 times hydrotherapy in 4 weeks, duration of sessions 60 min, 30 of them WATSU
**Outcomes**	Whoquol-bref "One mentioned remarked improvement in her satisfaction concerning her own health, together with energy, and capability to perform her daily activities. The other related an increasing satisfaction concerning her health after the treatment, and began to enjoy life much more, with more everyday energy."
**Notes**	33.35° C

Nogueira et al., 2017

**Methods**	Case report
**Participants**	61-year-old female patient with brachial plexus involvement, accessory nerve and depressive symptoms
**Interventions**	18 sessions of hydrotherapy: "heating (5 minutes), stretching (10 minutes of 3 repetitions of 5 seconds each), strengthening (30 minutes), and cooling (5 minutes). "
**Outcomes**	range of motion, pain and depression improved
**Notes**	"...and in some sessions were used Halliwick techniques and Watsu, in adduction and abduction, using cervical collar, pelvic belt, noodles and cervical traction techniques. Deterioration with walking or buoyancy."

Oh et al., 2015

**Methods**	Description of the development of an aquatic treatment program
**Participants**	workers with chronic musculoskeletal disorders

**Interventions**	"The aquomanual therapy program was 60 minutes long: 10 minutes to adapt to the water, 40 minutes of treatment, and 10 minutes of cool-down. The body parts included the cervical spine, thoracic spine, shoulder complex, lumbar, and lumbar spine. The treatments consisted of four static stretches, manually assisted movements, mobilization, and manipulation (Table 2). The aquomanual therapy program was organized by body part and consisted of four detailed treatments.<br>Therapy was delivered on a one-to-one ratio of therapist to worker, and each patient wore an aqua inflatable neck collar (#707; Sprint, USA) and waist belt (aqua belt; SAEHAN, KOREA) to float in the supine position in the water."
**Outcomes**	program development
**Notes**	25 °C

Orsini et al., 2009

**Methods**	Case report
**Participants**	1 patient with hereditary spastic paraparesis (HSP), Strumpell-Lorrain disease
**Interventions**	Aquatic exercise program included passive stretching, mobilisation, active training, gait training, balance training, muscular strength training and Watsu for relaxation.<br>The therapeutic program implemented, after a detailed kinetic-functional diagnosis, aims to adjust muscle tone, increase mobility and dissociation of waists (scapular and pelvic); Controlling pain, facilitating the gait oscillation phase, training adequate weight transfers and improving balance and protection reactions (Table 3). 3x 50min
**Outcomes**	Results were not possible due to the recent entry of the individual into the therapeutic proposal.
**Notes**	Watsu only part of the program (Relaxation)<br>Temperature:33–34° C

Park et al., 2015

**Methods**	Case series
**Participants**	13 hemiplegic patients
**Interventions**	3x/week 30min, 4 weeks: "The aquatic-therapy regimen was based on Halliwick, WATSU, and trunk-specific exercise methods, and its purpose was to improve the activation of trunk muscles associated with gait parameters in stroke patients. Aquatic exercise sessions consisted of a warm-up, trunk-specific exercises, and then a cool-down period (Table 1)18–20). The basic exercises in the Halliwick method for balance restoration concentrate on sagittal rotation control (bending from left to right or transferring weight while in an upright position), longitudinal rotation control (the ability to control movement around the sagittal-frontal axis whether in the vertical position or floating horizontally, e.g., to rotate in the same place while standing or to do so while supine and floating prone with one’s face in the water), transversal rotation control (around the transverse axis with the subject moving from standing to supine and then returning to the standing position), and combined rotation control (a combination of transverse sagittal and

	longitudinal rotations) (Fig. 1)21). WATSU is a body-based method comprising buoyancy, passive stretching, and massage techniques, including massage and palpation of acupuncture points administered in warm water"
**Outcomes**	Gait parameters were measured using a gait analysis system (Gait Trainer 2 analysis system, Biodex Medical Systems Inc., Shirley, NY, USA). Electromyographic signals were measured for the rectus abdominis, external abdominal oblique, transversus abdominis/ internal-abdominal oblique, and erector spine of each patients.
**Notes**	30°, WATSU in the last 5 minutes of the session as cool down

Park et al., 2016

**Methods**	CT
**Participants**	28 hemiplegic patients (15 land, 13 water)
**Interventions**	Halliwick and WATSU or land-based therapy, 30min, 3x/week, 4 weeks
**Outcomes**	Walking speed and cycle, stance phase and stride length of the affected side, and the symmetry index of the stance phase significantly improved after the aquatic and land-based trunk exercise program.
**Notes**	30 °C

Pattman et al.,, 2013

**Methods**	Case series
**Participants**	125 patients with musculoskeletal, orthopedic, rheumatology, or neurological conditions
**Interventions**	initial individual treatment by a trained aquatic physiotherapist was given.<br>Once a patient’s response to AP had been determined, group therapy (maximum of six patients per group) was continued. Patients with less complex needs were treated, after assessment, by technical instructors, under the guidance and protocols of the aquatic physiotherapist in a group session.<br>AP consists of a number of physiotherapy techniques conducted during immersion in warm water. At the BSUH, various techniques are employed during AP, including exercise methods (e.g., Halliwick, Bad Ragaz, Ai Chi, modified Mackenzie, modified pilates), relaxation (supine floating, Watsu), and manual therapy (e.g., Maitland mobilizations).
**Outcomes**	MYMOP2 (Measure Yourself Medical Outcome Profile) Questionnaire
**Notes**	Watsu was only part of various techniques in AP<br>Temperature: 35 °C

Romeiro & Navarro, 2011

**Methods**	Case report
**Participants**	male, 15 years, Guillain-Barré Syndrom

**Interventions**	"In order to finish the session, we decided to perform the seaweed of the Watsu method, since in such practice the patient remains completely passive and experiences a deep relaxation (RUOTI; MORRIS; COLE, 2000)." 11 times 45 minutes of combined aquatic treatment.
**Outcomes**	"a functional improvement of the trunk and limbs and reported this improvement in their activities of daily living", "As a result it could be observed that the proposal of aquatic treatment was efficient, because it provided the patient with a bigger independence in relation to the activities of daily life which had the functional improvement of the trunk and the members. Soon, the aquatic therapy can be very efficient in the late illness, and thus it fits to the physiotherapists as well as other health professionals, to stimulate and make these patients more aware about the illness."
**Notes**	34 °C

Santana et al., 2005

**Methods**	Case report
**Participants**	C5-7 severe transverse myelopathy patient, 32 years, female
**Interventions**	The primary objective was to compare the results obtained during the conventional treatment with those of hydrotherapy<br>Transfer training for entry into the pool;<br>● Decoupling of pelvic girdle: passive pelvic rotation in the supine float, with stabilization of the patient's trunk near the edge of the pool;<br>● Watsu: with the breath of dance techniques, rotating with the next leg, Knee-head, hip balance;<br>● passive mobilization of lower limbs: with rotation exercises, flexion, adduction and abduction hips, flexion-extension of the knee and passive full drive tibio-tarsal joint. These mobilizations are accompanied by stretching muscle;<br>● Active rotation of the pelvis: after stabilization of the trunk, the patient performs rotation, as well as tilt and active in order to strengthened oblique muscles and side of the stem;<br>● Muscle strengthening: the muscle groups of the upper limbs, essential for functionality transfers of paraplegic patients;<br>● Remove the manual contact the patient's skin: it is important to give more sense of independence and autonomy
**Outcomes**	At the end of treatment, the patient showed increased relaxation. It was also noticed an exponential increase in the possibility of held passive mobilization work in all joints of the lower limbs, facilitated for muscle relaxation and the normalization of tone previously achieved
**Notes**	Temperature: "warm". WATSU is only part of the program.

Santos & Facci, 2009

**Methods**	Case series
**Participants**	19 patients were included, (18 women, one man), mean age 49,63 years
**Interventions**	20 water physiotherapy sessions, three times a week, with 5 people in each group, lasting 45 minutes each session
**Outcomes**	Fibromyalgia Impact Questionnaire<br>Number of painful points of the human body<br>Pain intensity<br>"meaningful statistically improvement related to the number of painful points of the human body (p=0,016) in pain intensity (p=0, 04) and in some Fibromyalgia Impact Questionnaire items."
**Notes**	Watsu only part of therapy session, group sessions<br>Temperature: 32° to 35°

Santos et al., 2018

**Methods**	Controlled trial
**Participants**	Twenty-four men and women (67.83 ± 5.70 years) diagnosed with hypertension
**Interventions**	Intervention group: at least 6 month of hydrotherapy twice a week for 50 minutes "10 minutes of warm-up (gait), 20 minutes of aerobic exercises (jumping, dancing and pedaling activities, besides 10 minutes of strengthening muscles of the upper limbs, lower limbs and abdominals, playful activities that involved balance and coordination and relaxation in the final 10 minutes with the Watsu method."<br>Control group: no hydrotherapy
**Outcomes**	"hypertensive elderly patients who underwent regular aquatic physiotherapy presented better functional capacity and there were no differences in quality of life among hypertensive elderly individuals between groups."
**Notes**	32° C, 10 minutes WATSU only

Silva & Navarro, 2006

**Methods**	Case report
**Participants**	patient 44 years old, female, late post bariatric surgery
**Interventions**	two weekly sessions of hydrotherapy and three of ground physiotherapy for a period of 4 months
**Outcomes**	6 minutes walk and the SF-36
**Notes**	Temperature not stated. Watsu only for relaxation at the end of hydrotherapy session

Silva et al., 2006

**Methods**	Case report
**Participants**	female, 44 years, obese
**Interventions**	2 weekly hydrotherapy sessions over 4 months
**Outcomes**	6-minutes walk test: increase of 90 meters; SF-36: increase in 6 of 8 scales

**Notes**	15 minutes of WATSU to cool down

Smeeding & Osguthorpe, 2005

**Methods**	Report
**Participants**	573 soldiers, unclear, how many actually received WATSU.
**Interventions**	"13 therapies and classes offered though the IHCC include acupuncture, Choosing to Heal classes, guided imagery for surgical preparation, herbal/nutritional supplement consultation with a doctor of pharmacy, medical hypnosis, meditation, non-denominational prayer group, qigong movement classes, watsu, yoga, Native American healing ceremonies, multidisciplinary Quit Smart Tobacco Cessation group class, and the Fit and Trim Weight Management program."
**Outcomes**	"Movement therapies such as yoga, qigong, and watsu have helped improve patient mobility and reduce pain."
**Notes**	Temperature: "heated", durationand amount of WATSU-sessions not mentioned. No conclusions can be drawn to the effects of WATSU. Reports on same project as "Smeeding et al., 2011" and "Smeeding et al., 2010"

Smeeding et al., 2010

**Methods**	Retrospective post-hoc quasi-experimental design
**Participants**	165, soldiers after combat: chronic nonmalignant pain and stress-related depression, anxiety, and symptoms of post-traumatic stress disorder (PTSD). n(WATSU) = ?
**Interventions**	WATSU and Jahara-Technique, also parallel therapies involved (10 holistic, nonpharmacological mind-body skills and CAM therapies)
**Outcomes**	effective reduction in pain-related psychopathology, depression, and anxiety and may improve some aspects of HRQOL through use of nonpharmacological and nontraditional mind-body skills and CAM therapies. "All of the comparison groups demonstrated an improvement in depression and anxiety scores, as well as in some SF-36 categories. The subgroups with the greatest improvement, seen at 6 months, were found in the high anxiety group (Cohen's d = 0.52), the high-depression group (Cohen's d = 0.46), and the PTSD group (Cohen's d = 0.41)."
**Notes**	Temperature and duration of WATSU not mentioned, apparently also Jahara-technique involved. No conclusions can be drawn to the effects of WATSU. Reports on same project as "Smeeding & Osguthorpe, 2005" and "Smeeding et al., 2011"

Smeeding et al., 2011

**Methods**	Retrospective post-hoc quasi-experimental design with a group analysis comparing chronic nonspinal-related pain (CNSP) (eg, joint pain, headache, and fibromyalgia) (n=53) to chronic spinal-related pain (CSP) (eg, back pain and neck pain) (n=88)
**Participants**	53 patients with CNSP and 88 patients with CSP. n(WATSU) = ?

**Interventions**	mind-body therapies: Treatment plans were very individualized based on patient choice and provider assessment and provided an option to try other therapies and classes when appropriate.<br>Integrative Health Clinic Intake:Chief complaint, pain and medical history, conventional and CAM therapy history, social support, emotional and mental health assessment, description, risk/benefit of IHCP therapies and classes, physical exam, computerized outcome tests (BDI, BAI, SF-36).<br>Treatment plan is collaborative, with the patient as primary director of plan. Referrals are sent to conventional services: physical therapy, anesthesia interventional pain service, medical, surgical or mental health as needed; orders are written, appointments made for IHCP therapies/classes as determined by the plan<br>Acupuncture:Trial of 4-6 treatments/one/wk, continuation and frequency based on response and evaluation thereafter<br>Aquatic Bodywork (WATSU):Trial of 6-8 sessions, continuation based on response and evaluation.Provided in the VASLC heated swimming pool<br>Choose to Heal Stress management:8wk stress management, mind-body skills experiential: meditation,guided imagery, relaxation techniques, biofeedback/autogenic training, qigong, self-awareness of thought patterns, reframing of negative thought patterns<br>Herbal/nutritional supplement/drug interaction education and counseling:The VA does not prescribe herbal or nutritional supplements, however if a patient chooses to take supplements and prescription medication the Pharm. D. counsels on the risk/benefit<br>Medical Hypnosis<br>Meditation<br>Qigong<br>Tobacco Cessation VA Quit Smart program with Tobacco Cessation hypnosis<br>Weight Management<br>Yoga
**Outcomes**	Integrative Health Clinic Follow-up at 6mo, 1 y, 2 y and as needed by the treatment plan<br>Short Form-36 (SF-36):physical function, bodily pain, vitality, social function, role physical, general health, role emotional, mental health<br>Beck Depression Inventory (BDI)<br>Beck Anxiety Inventory<br>NRS (VAS) Pain<br>Service connection for pain<br>"CAM therapies were chosen for inclusion into the model because they emphasize treatment of the whole person, integrating psychological, social, spiritual, and physical care, and can easily be coupled with MBSs that promote self-empowerment through self-management techniques. IHCP therapies/classes include: acupuncture, aquatic bodywork, stress management, hypnosis, meditation, qigong, yoga, herb/drug interaction counseling, multidisciplinary weight management, and tobacco cessation classes (Table 1). The therapies and classes are designed to have a biopsychosocial synergistic effect, as each therapy and class includes the IHCP philosophy of thought and body awareness, use of stress and pain selfmanagement tools, and promotion of an internal locus of control to enhance self-efficacy, coping, and wellness behaviors."

**Notes**	Temperature: "heated", duration of WATSU-sessions not stated. No conclusion can be drawn to the effects of WATSU. Reports on same project as "Smeeding & Osguthorpe, 2005" and "Smeeding et al., 2010"

Taketa et al., 2018

**Methods**	Case report
**Participants**	15-year-old male patient of juvenile ankylosing spondylitis
**Interventions**	Sixty sessions of hydrotherapy were performed individually in a heated pool at 32 ° C. -34 ° C, twice a week, lasting 60 minutes. The exercise protocol included: warm-up, stretching, trunk, pelvic, lower and upper limb mobility, aerobic exercise, and relaxation.
**Outcomes**	improvement in pain, mobility, function, disease activity and quality of life
**Notes**	"Relaxation (5-10 minutes) Performed with castor bean in trapezoid region. Passive mobilization of the Watsu method"

Tanoue et al., 2009

**Methods**	Case report
**Participants**	female, 45 years; onset of fibromyalgia 5 years ago; joint pain, restricted range of motion in flexion and abduction of shoulders, bursitis and tendinitis in the glenohumeral joint bilaterally
**Interventions**	combination of aquatic interventions, 22 sessions of 45 minutes in 11 weeks "The therapies were finalized by performing the previously described passive stretches and relaxation methods such as Watsu: applying the breathing dance techniques, accordion, rotational accordion, pendulum, inside and outside leg balance, seaweed and hip."
**Outcomes**	visual analogue pain scale, the degree of Oxford muscle strength of the deltoid muscles, major and minor pectoralis, biceps, triceps and large dorsal muscles, and range of motion of the abductors and shoulder flexors. The quality of life evaluation was performed through the application of the generic Medical Outcomes Study 36-item Short-Form Health Survey (SF-36), with items on: depression, stiffness, pain, anxiety and limitations in activities of daily living ADL. It was observed that the proposed treatment, improved the range of motion and of life quality of the patient.
**Notes**	Temperature: not reported, only some WATSU at the end of an aquatic program

Tonieto et al., 2015

**Methods**	pre-post treatment study
**Participants**	Six patients with a diagnosis of stroke
**Interventions**	18 sessions of aquatic therapy, mixed approach of several techniques (Halliwick, Bad Ragaz, Watsu)

**Outcomes**	Stroke Specific Quality of Life Scale (SS-QOL) to assess the quality of life, the Test of 6-minute walk, to assess the functional capacity and the Walk Test of 10 meters to measure gait speed<br>"increase in quality of life, gait speed, and functional capacity"
**Notes**	Temperature: 33 a 36ºC, 45 min

Tüfekçioğlu & Çotuk, 2009

**Methods**	Case series
**Participants**	18, 25-35 years
**Interventions**	Each measurement was taken at three different locations, namely landing (vertical), lying (horizontal) and water (watsu). "In the Watsu position, a large part of the body weight (85-90%) is left in the water. In order not to adversely affect the measurements, the float-assisted rods of foam material are placed in the head and neck areas of the knee. The upper part of the chest area of the person standing in horizontal position in the water is slightly above the water. The face of the chin is on the water so that it can breathe easily. The part up to the ears is in the water. Measurements were made at the time of applying the "water breath dance" technique (4). The subjects, who were supported by the investigator under the hand, were kept completely calm and immobile on the water. The measured pool water temperature is 30, the pool ambient temperature is 32 degrees and the humidity is 65%."
**Outcomes**	Participants' HRV values were recorded with the S810i of the Polar® telemetric pulse-gauge device. The time between heartbeats (RR interval) was recorded at 1 ms resolution.<br>The parameter we use in the time dimension analysis of HRV is RMSSD. The RMSSD is defined as the square root of the squared average of consecutive RR intervals. Frequency dimension analysis of HRV was performed with the power spectrum obtained by "Fast Fourier Transform". The frequency domain parameters are HF% and LF / HF. HF% indicates the high frequency ratio between 0.15-0.40 Hz in the power spectrum, while LF / HF is the low and high frequency power ratio (1).
**Notes**	30°. The description refers to one WATSU-maneuver only, which does not really constitute a WATSU-session.

Useros-Olmo & Collado-Vázquez, 2010

**Methods**	Pre-post treatment pilot study
**Participants**	16 patients (13 female and 3 male) diagnosed with cervical dystonia.<br>Inclusion: Be diagnosed with focal cervical dystonia for more than five years and have no scheduled toxin infiltration in the period of duration of the study.<br>The exclusion criteria were: To suffer degenerative neurological diseases or secondary to brain damage and having been infiltrated of toxin over a period of less than 12 weeks.<br>16 patients (n = 16), 13 women (81.3%) and three men (18.8%). The mean age of the sample Analyzed was 46.19 years and the median was 42.5, with a standard deviation of 12.32 and a range of 32-70 years. In terms of classification of dystonia, Of the 16 subjects, 10 presented laterocolis, three retrocolis and

	three cervical rotations.
**Interventions**	hydrotherapy treatment consisted of three individual sessions (WATSU) alternating with three group sessions of aquatic exercises, duration 40min
**Outcomes**	Toronto Western Spasmodic Torticollis Rating Scale (TWSTRS);<br>the balance was evaluated by means of the Get up & Go and Tinetti tests. In addition, the range of active mobility, flexibility of the neck was measured: Flexion, Lateroflexion and rotation (The articular path of the neck (active range) was measured by tape measure for flexion movements (chin-to-chest distance), lateroflexion (ear lobe distance and opposite acromioclavicular joint), rotation (chin-acromioclavicular joint distance on the same side. For flexion, the patient was measured in the supine position, and for the lateroflexion and rotation, in a chair with a backrest, in order to stabilize the patient to control the compensations.)<br>self reported outcome about improvement (10 questions) and satisfaction about the program (10 questions)<br>The test were measured pre and post-treatment.<br>The Student t showed a significant difference ($p < 0, 01$) in all the values. The range of active mobility of the neck improved in all movements:<br>flexion (1.3 ± 1.0 cm), right lateralization (3.4 ± 1.7 cm) and left (4.0 ± 3.0 cm) and right rotation (1.6 ± 2.5 cm) and left (2.2 ± 1.5 cm).<br>At the same time, improvements in: Tinetti (3.0 ± 2.2), Get up & Go (2.3 ± 1.6) and TWSTRS (8.4 ± 5.4). Regarding the descriptive analysis of the Questionnaire of perceived improvement and satisfaction patients reflected a level of improvement (average: 42.06) and Satisfaction (average: 48.38) very high.
**Notes**	Temperature: 33 °C No conclusions can be drawn to the effects of WATSU.

Useros-Olmo et al., 2018

**Methods**	quasi-experimental study with experimental and control groups without random assignment.
**Participants**	diagnosed with cervical dystonia by a neurologist at least 5 years before the beginning of the study.<br>Excluded when they had secondary dystonia or had scheduled an infiltration of botulinum<br>toxin during the clinical intervention.<br>27 people were enrolled in the research. Of these people, 15 were interested in receiving the clinical interventions. (=12 controls)
**Interventions**	The experimental group received an aquatic therapy known as “Watsu” (Dull, 2004) and<br>autogenic relaxation training (Schultz and Luthe, 1959) for 1 month; the control group did not receive treatment.<br>Details: four individual sessions (one per week, 50 min) of an aquatic therapy known as Watsu therapy (WT) and autogenic training (AT) over 1 month. After each WT session, participants completed 15 min of AT in a room adjacent to a swimming pool. The standard procedure developed by the German psychiatrist Johannes Schultz was applied (Luthe, 1969; Schultz and Luthe, 1959).<br>In addition, they practiced AT for 15 min, twice a day at home, with instructions provided via an audio compact disc.

**Outcomes**	questionnaires QoL, pain, and mood:<br>36-Item Short-Form Health Survey SF-36<br>Spasmodic Torticollis Rating Scale (TWSTRS)<br>Visual Analog Scale (VAS)<br>Beck Depression Inventory (BDI-II)<br>State-Trait Anxiety Inventory (STAI)<br>Experimental group (N=15)<br>Pre (M±SD) Post (M±SD) Diff means, SD diff, (95% IC) d p<br>Physical role 25.00 ± 41.19 43.33 ± 44.79 – 18.33 33.36 (– 36.81 a 0.14) 0.43 < 0.05<br>Physical function 57.00 ± 29.57 57.33 ± 11.11 – 0.33 11.87 (– 6.91 a 6.24) 0.02 ns.<br>Body pain 36.20 ± 24.25 54.80 ± 24.35 – 18.60 14.23 (– 26.48 a – 10.72) 0.77 < 0.001<br>General health 37.20 ± 15.63 42.27 ± 19.89 – 5.07 14.43 (– 13.06 a 2.93) 0.29 ns.<br><br>Vitality 38.20 ± 24.96 55.73 ± 21.34 – 17.53 18.97 (– 28.04 a – 7.03) 0.76 < 0.01<br>Social function 47.50 ± 29.58 70.00 ± 25.35 – 22.50 26.39 (– 37.11 a – 7.89) 0.82 < 0.01<br>Mental health 44.27 ± 23.54 61.87 ± 18.02 – 17.60 17.81 (– 27.46 a – 7.74) 0.85 < 0.01<br>Emotional role 28.84 + 41.53 51.07 + 48.60 – 22.22 43.00 (– 46.04 a 1.58) 0.49 ns.<br>Pain scale (TWSTRS) 11.13 + 6.53 8.87 + 5.68 2.27 2.43 (0.92 a 3.61) 0.37 < 0.01<br>Pain scale (VAS) 4.80 + 3.23 2.27 + 2.19 2.53 2.36 (1.23 a 3.84 0.93 < 0.01<br>Depression (BDI-II) 17.40 + 9.82 13.33 + 7.74 4.07 8.16 (– 0.45 a 8.59) 0.46 ns.<br>Anxiety (STAI) 71.13 + 28.14 60.67 + 34.04 10.47 24.86 (– 3.30 a 24.24) 0.34 ns.<br>Control group (N = 12)<br>Pre (M±SD) Post (M±SD) Diff means, SD diff, (95% IC) d p<br>Physical role 50.00 ± 45.23 52.08 ± 40.53 – 2.08 22.51 (– 16.38 a 12.22) 0.05 ns.<br>Physical function 63.33 ± 25.35 61.67 ± 23.09 1.67 18.50 (– 10.09 a 13.42) 0.07 ns.<br>Body pain 55.08 ± 30.13 55.50 ± 22.30 – 0.42 18.88 (– 12.41 a 11.58) 0.02 ns.<br>General health 44.08 ± 16.82 43.00 ± 22.65 1.08 19.53 (– 11.32 a 13.49) 0.05 ns.<br><br>Vitality 41.75 ± 26.70 42.08 ± 20.94 – 0.33 21.67 (– 14.10 a 13.43) 0.01 ns.<br>Social function 64.58 ± 23.13 71.88 ± 20.03 – 7.29 13.55 (– 15.90 a 1.31) 0.34 ns.<br><br>Mental health 59.75 ± 19.34 59.83 ± 16.15 – 0.08 10.13 (– 6.52 a 6.35) 0.00 ns.<br>Emotional role 86.08 ± 33.26 61.07 ± 42.26 25.02 53.44 (8.94 a 58.97) 0.66 ns.<br>Pain scale (TWSTRS) 6.42 ± 6.22 8.00 ± 6.81 – 1.58 3.00 (– 3.49 a 0.32) 0.24 ns.<br><br>Pain scale (VAS) 3.33 ± 3.75 3.42 ± 3.37 – 0.08 1.24 (– 0.87 a 0.70) 0.02 ns.<br>Depression (BDI-II) 11.75 ± 7.25 12.33 ± 7.35 – 0.58 3.37 (– 2.72 a 1.56) 0.08 ns.<br><br>Anxiety (STAI) 60.67 ± 28.02 62.08 ± 28.35 – 1.42 21.06 (– 14.80 a 11.96) 0.05 ns.
**Notes**	50 min, 33-35 °C

Vogtle et al., 1998

**Methods**	Case series
**Participants**	Six adults with cerebral palsy
**Interventions**	aquatic therapy 2 days a week for 7 weeks, approximately 45 minutes of water shiatsu (WATSU) using a modified head cradle sequence and approximately 15 minutes of Halliwick method activities focused on head, trunk, and extremity movement control.
**Outcomes**	Outcomes suggest that the program was effective for improving range of motion, decreasing pain, and providing a pleasurable social experience. Benefits were also realized by the students participating in the swim program, including skill development and appreciation of patients with disability as individuals.
**Notes**	32° C

Zanella & Romero, 2011

**Methods**	Case series
**Participants**	5 patients with spinal cord injury, with an average of 2 years of injury and average of age 20
**Interventions**	soil treatment was based on methods of PNF<br>treatment in water was based on the Bad Ragaz<br>method that acts restoring normal patterns of movement, helping in the muscle strengthening and the Watsu method that provides a deep state of relaxation, modulating tone pathological and cinesiotherapy adapted to the water.
**Outcomes**	SF 36
**Notes**	Temperature: not mentioned

Footnotes
