# Supplementary material for: Applications, indications, and effects of passive hydrotherapy WATSU (WaterShiatsu)—A systematic review and meta-analysis
Source: PLoS One. 2020 Mar 13;15(3):e0229705. doi: 10.1371/journal.pone.0229705 (PMC7069616; doi:10.1371/journal.pone.0229705)
Supplement: S4 File — (PDF) [file pone.0229705.s009.pdf]

Applications, Indications, and Effects of Passive Hydrotherapy WATSU

References to studies

Excluded studies

Araújo & Assis, 2018x

Araújo DS, Assis SCd. Effects of aquatic physiotherapy in the treatment of amyotrophic lateral sclerosis [Efeitos da fisioterapia aquática no tratamento da esclerose lateral amiotrófica]. Fisioterapia Brasil 2018;19(5):170-175.

Becker & Lynch, 2017x

Becker BE, Lynch S. Case Report: Aquatic Therapy and End-Stage Dementia. PM R Sept 14 2017;XXX:1-5. [DOI: 10.1016/j.pmrj.2017.09.001]

Bonetti et al., 2010x

Bonetti A, Cantos GA, Frutuoso JT, Rocha ME. Interdisciplinary and Multiprofessional Activities Cardiovascular Diseases Prevention Program Experience Report [Atividades interdisciplinares e multiprofissionais: relatos de experiência com participantes do programa de prevenção para doenças cardiovasculares Extensio: Revista Eletrônica de Extensão]. Revista Eletrônica de Extensão 2010;7(10):70-89.

Cantos & Souza, 2013x

Cantos GA, Souza LC. Follow Up of Two Diabetic Patients by Interdisciplinary and Mulitprofessional Team [Acompanhamento de dois pacientes diabéticos por equipe interdisciplinar e multiprofissional]. Extensio: Revista Eletrônica de Extensão 2013;9(14):81-91. [DOI: 10.5007/1807-0221.2012v9n14p81]

Cantos et al., 2008x

Cantos GA, Schütz R, Rocha ME. Association of the Techniques of Watsu and Halliwick with the Aquatic Biodanza, as Form to Improve the Psychological Stress of Patients with Chronic Deseases [Associação das técnicas de Watsu e Halliwick com a Biodanza® aquática, como forma de melhorar o estresse psicológico de pacientes com doenças crônicas]. Revista Pensamento Biocêntrico 2008;9(Jan/Jun):69-83.

Cantos et al., 2008xa

Cantos GA, Schütz R, Rocha ME, Marques CdSdS, Balén MdGW, Hermes E. Association of Methods Watsu, Halliwick, and Biodanca: Preliminary Data of a Population with Chronic Diseases [O método watsu e halliwick associados com a biodança: dados preliminares de uma população com doenças crônicas]. Pensamento Biocêntrico 2008;10(Jul/Dec):27-41.

Cantos et al., 2013x

Cantos GA, Corrêa TA, Bonetti A, Wommer D. Contribution of a Body Practices Recreational in the Prevention of Cardiovascular Disease [Contribuição de um programa de práticas corporais lúdicas na prevenção de doenças cardiovasculares]. Extensio: Revista Eletrônica de Extensão 2012;9(14):73-80. [DOI: 10.5007/1807-0221.2012v9n14p73]

Chang et al., 2009x

Chang KH, Ryu JK, Jung YJ. Validation of the Effect of Aquatic Exercise Program Designed to Improve Motor Function of Hemiplegic Patients. Jounal of Adapted Physical Activity 2009;17(4):19-34.

Costa et al., 2017x

Costa MRDVd, Lima RCd, Lopes CP, Shirahige L, Albuquerque PL. Effects of hydrotherapy on the endurance and quality of life instroke survivors [Efeito da hidroterapia nocondicionamento cardiovascular ena qualidade de vida de pacientesapós acidente vascular encefálico]. ConScientiae Saúde 2017;16(2):259-265. [DOI: 10.5585/ConsSaude.v16n2.6936]

Degani & Villa, 2005x

Degani AM, Villa PdS. Range of Movement and Health Related Quality of Life in Patients with Juvenile Idiopatic Arthiritis Submitted to Aquatic Physical Therapy [Amplitude de Movimento Articular e Qualidade de Vida Relacionada á Saúde de Portador de Artrite Idiopática Juvenil Submetido á Fisioterapia Aquática]. Fisioterapia em Movimento 2005;18(4):33-42.

Elsner et al., 2009x

Elsner VR, Trentin RP, Horn CC. The Effects of Hydrotherapy in the Quality of Life of Women Undergoing Mastectomy [Efeito da hidroterapia na qualidade de vida de mulheres mastectomizadas]. Arquivos de Ciências da Saúde 2009;16(2):67-71.

Eo & Lee, 2011x

Eo S-J, Lee E-H. Effect of Water Exercise on Fitness Factor and Delivery in Pregnant Women. The Korean Journal of Sport 2011;9(3):571-578.

Ferreira & Matsutani, 2006x

Ferreira KB, Matsutani LA. Hydrotherapy approach in the treatment of fibromyalgia [Abordagem da hidroterapia no tratamento da fibromialgia]. Revista PIBIC (Programa Institucional de Bolsas de Iniciação Científica) 2006;3(2):39-47.

Gimenes et al., 2007x

Gimenes RO, Previato BL, Claudio PDS, Santos ECd. Repercussion of a hydrokinesiotherapy program in the life and pain satisfaction of patients with herniated disc [Repercussão de um programa de hidrocinesioterapia na satisfação de vida e dor de pacientes com hérnia de disco]. In: Congresso Internacional de Postura - I Edição - 2007. Rio de Janeiro , Brasil, 2007. [Other: ISSN 2359-411X]

Gimenes et al., 2008x

Gimenes RO, Carvalho NTPd, Farelli BC, Mello TWPd. Impact of Aquatic Physiotherapy on Arterial Pressure of Aged People [Impacto da fisioterapia aquática na pressão arterial de idosos]. Ö Mundo da Saúde 2008;32(2):170-75.

Gimenes et al., 2008xa

Gimenes RO, Previato BL, Claudio PDS, Santos ECd. Impact of the spinal school program on individuals with lumbar disc herniation [Impacto do programa escola de coluna em indivíduos com hérnia de disco lombar]. Revista Dor 2008;9(2):1234-1241.

Gonçalves et al., 2009x

Gonçalves PV, Santos WC, Peternella FMN. Assessment of an aquatic therapy program in treatment of thoracic ravine syndrome: case report [Avaliação de um programa de terapia aquática no tratamento da síndrome do desfiladeiro torácico: relato de caso]. Revista UNINGÁ, Maringá 2008;20:161-170.

Jacintho et al., 2008x

Jacintho RLM, Galvão LLLF, Araújo AGR, Andrade SCd. Evaluation of the Life Quality of Women with Fibromyalgia After Aquatic Physical Therapy [Avaliação da qualidade de vida em mulheres com fibromialgia após fisioterapia aquática]. Revista da FARN (Faculdade Natalense para o Desenvolvimento do Rio Grande do Norte) 2008;7(1):13-27.

Jakaitis & Guazelli, 2005x

Jakaitis F, Guazzelli ABA. Study of the effect motor-sensories of the aquatic therapy withpatients in vigil coma stage [Estudo dos efeitos sensório-motores dafisioterapia aquática com pacientes emestado de coma vigil]. Revista Neurociências 2005;13(4):215-18.

Kakihara & Neves, 2005x

Kakihara CT, Neves CG. Functional assessment of neurological patients with stroke, before and after physical therapy and hidrotherapy [Avaliação do grau de funcionalidade de pacientes que sofreram acidente vascular encefálico antes e após intervenção fisioterapêutica no solo e na hidroterapia]. Fisioterapia Brasil 2005;6(5):332-338.

Kim & Lee, 2015x

Kim BO, Lee HJ. Effects of Aquatic Exercise Therapy on Motor Functionand Balance in Children with Spastic Cerebral Palsy. Journal of Special Educaton & Rehabilitation Science 2015;54(1):75-94.

Kim et al., 2009x

Kim MG, Hong YJ, Koo KS. The Effects on the Gross Motor Skills of the Children with Hemiplegia by Aquatic Rehabilitation Exercise. Korean Society for Developmental Biology 2009;17(2):99-108.

Kwangmin et al., 2016x

Kwangmin R, Asif A, Minji K, Changyoung L, Yujin K, Gyusung L, Jingu K. Effects of assisted aquatic movement andhorseback riding therapies on emotion and brainactivation in patients with cerebral palsy. The Journal of Physical Therapy Science 2016;28:3283-3287.

Lee & Kim, 2015x

Lee CY, Kim CH. The Effects of Water Rehabilitation Exercise on Emotion of Children with Encephalopathy: A Study Using Electroencephalography. The Korenan Journal of Growth and Development 2015;23(2):171-79.

Lotan & Barmatz, 2009x

Lotan M, Barmatz C. Hydrotherapy for a young child with Rett syndrome. Review of the literature and a case study. International Journal on Disability and Human Development 2009;8(3):349-57.

Maczkowiak et al., 2007x

Maczkowiak S, Hölter G, Otten H. WATSU - on the effectiveness of differently accentuated movement-therapeutic tnterventions in clinically depressed patients [WATSU – Zur Wirksamkeit unterschiedlich akzentuierter bewegungstherapeutischer Interventionen bei klinisch depressiven Patienten]. Bewegungstherapie und Gesundheitssport 2007;23:58-64.

Martins et al., 2015x

Martins LG, Rocha LPB, Veríssimo TCRA, Souza JdS, Prudente COM, Ribeiro MFM. Effects of Virtual Rehabilitation, Bobath Concept, and Aquatic Therapy in Children with Cerebral Palsy [Efeitos da reabilitação virtual, conceito Bobath eterapia aquática em crianças com paralisia cerebral]. Revista Neurociencia 2015;23(1):68-73. [DOI: 10.4181/RNC.2015.23.01.975.6p]

Masselli et al., 2009x

Masselli MR, Casoti A, Campos ACLd, Flores ALdM, Campos JI, Silvestre MR. Hydrotherapy for treating work related muskuloskeletal disorder [Hidroterapia no tratamento de pacientes com distúrbios osteomus-culares relacionados ao trabalho]. Revista da Sociedade Brasileira para o Estudo da Dor 2009;10(4):307-12.

Mazetto & Navarro, 2007x

Mazetto AA, Navarro FM. Benefit of the aquatic therapy in the arthritisrheumatoid: study of case [Benefício da terapia aquática na artrite reumatóide: estudo de caso]. Revista Uningá 2007;12:153-159.

Melo et al., 2012x

Melo FR, Alves DAG, Leite JMRS. Benefits of hydrotherapy for spasticity in children with hydrocephalus [Benefícios da Hidroterapia para Espasticidade em Uma Criança com Hidrocefalia]. Revista Neurociências 2012;20(3):415-21. [DOI: 10.4181/RNC.2012.20.707.7p]

Mesquita et al., 2007x

Mesquita MG, Caetano L, Pernambuco CS, Silva EB, Dantas EHM. Urinary excretion of hydroxyproline analysis and pain in subjects with low back pain after stretching in hydrotherapy [Análise da excreção urinária de hidroxiprolina e do nível de dor em sujeitos com lombalgia após alongamento na hidrocinesioterapia]. Fisioterapia Brasil 2007;8(2):99-102.

Nascimento et al., 2012x

Nascimento VLdS, Borba GdS, Leite CMdB, Garabini MC. Hydrotherapy Protocol In Guillain Barre’s Syndrome - Case Report [Protocolo Hidroterápico na Síndrome de Guillain-Barré- Estudo de Caso]. Revista Neurociencias 2012;20(3):392-8.

Navarro et al., 2006x

Navarro FM, Neto JOC, Benossi TG. The Effect Water Therapy in the Fibromyalgic Patients' Quality Life: Related of Case [Efeitos da terapia aquática na qualidade de vida de pacientes fibromiálgicos - estudo de caso]. Arquivos de Ciências da Saúde 2006;10(2):93-7.

Navarro et al., 2006xa

Navarro FM, Sato CT, Melo LCD. Hydrotherapy in the treatment of stroke: a case study [Hidroterapia no tratamento do acidente vascular cerebral: estudo de caso]. Revista Uningá 2006;7:151-156.

Nogueira et al., 2017x

Nogueira ER, Silva EPDC, Navarro FM, Tako KV. Effects of hydrotherapy on brachial plexus and accessory nerve injury: Case study [Efeitos da hidroterapia na lesão de plexo braquial e nervo acessório: Estudo de caso]. Revista Uningá 2017;8(1):109-118.

Oh et al., 2015x

Oh SJ, Yeom J-W, Kim M, Lee M, Yoon BC. Aquomanual Therapy Program Development for Workers with Chronic Musculoskeletal Disorders. Journal of Yoga & Physical Therapy 2015;5(4):1. [DOI: http://dx.doi.org/10.4172/2157-7595.1000212]

Orsini et al., 2009x

Orsini M, Mello MP, Calheiros M, Nascimento OJM, Freitas MRGd. Hydrotherapy for spasticity in Strumpell-Lorrain disease: case report [Hidroterapia para espasticidade na doença de Strumpell-Lorrain: relato de caso]. Revista Neurociencia 2009;17(1):67-71.

Park et al., 2015x

Park B-S, Noh J-W, Kim M-Y, Lee L-K, Yang S-M, Lee W-D, Shin Y-S, Kim J-H, Lee J-U,Kwak T-Y, Lee T-H, Kim J-Y, Park J, Kim J. The effects of aquatic trunk exercise on gait and muscle activity in stroke patients: a randomized controlled pilot study. Journal of physical therapy science 2015;27(11):3549.

Park et al., 2016x

Park B-S, Noh J-W, Kim M-Y, Lee L-K, Yang S-M, Lee W-D, Shin Y-S, Kim J-H, Lee J-U, Kwak T-Y, Lee T-H, Park J, Kim J. A comparative study of the effects of trunk exercise program in aquatic and land-based therapy on gait in hemiplegic stroke patients. The Journal of Physical Therapy Science 2016;28:1904-8.

Pattman et al., 2013x

Pattman J, Hall J, Record E. Effectiveness of Aquatic Physiotherapy in Clinical Practice. International Journal of Aquatic Research and Education 2013;7:396-406.

Romeiro & Navarro, 2011x

Romeiro TC, Navarro FM. Aquatic therapy in the Guillain-Barré Syndrome: case study [Terapia aquática na síndrome de Guillain-Barré: estudo de caso]. Revista UNINGÁ, Maringá 2011;29:121-129.

Santana et al., 2005x

Santana JMd, Filho VJdS, Almeida RDd. Hydrokinesiotherapeutic Approach for Bearers of Severe Transverse Myelitis [Abordagem hidrocinesioterapêutica para portadores de mielite transvera grave]. Revista HISPECI & LEMA 2005;8:77-80.

Santos & Facci, 2009x

Santos STd, Facci LM. Aquatic Exercise Therapy in Fibromyalgia: Serie of Cases [Hidrocinesioterapia na Fibromialgia: Série de Casos]. Revista Saúde e Pesquisa 2009;2(3):427-32.

Santos et al., 2018x

Santos SBd, Oliveira Santos Md, Ferreira LL. Influence of aquatic physiotherapy on the functional capacity andquality of life of hypertensive elderly [Influência da fisioterapia aquática na capacidadefuncional e qualidade de vida de idosos hipertensos]. Revista Interdisciplinar De Promoção Da Saúde 2018;1(1):7-13. [DOI: http://dx.doi.org/10.17058/rips.v1i1.11940]

Silva & Navarro, 2006x

Silva EPDC, Navarro FM. Repercussion of hydrotherapy in atrophy oligopontocerebellar: report of a case [Repercussão da hidroterapia na atrofia oligopontocerebelar: relato de um caso]. Revista Uningá 2006;9:119-125.

Silva et al., 2006x

Silva TJPV, Telles GCQ, Gimenes RO, Trimer R. Hydrotherapy and cardiovascular rehabilitation: A new approach on late post-operative rehabilitation after bariatric surgery [Hidroterapia e reabilitação cardiovascular: Uma nova abordagem no pós-operatório tardio de cirurgia bariátrica]. O mundo da Saúde São Paulo 2006;30(1):179-184.

Smeeding & Osguthorpe, 2005x

Smeeding S, Osguthorpe S. The Development of an Integrative Healthcare Model in the Salt Lake City Veterans Affairs Healthcare System. Alternative Therapies in Health and Medicine 2005;11(6):46-51.

Smeeding et al., 2010x

Smeeding SJW, Bradshaw DH, Kumpfer K, Trevithick S, Stoddard GJ. Outcome Evaluation of the Veterans AffairsSalt Lake City Integrative Health Clinic for ChronicPain and Stress-Related Depression, Anxiety,and Post-Traumatic Stress Disorder. The Journal of Alternative and Complementary Medicine 2010;16(8):823-835. [DOI: 10.1089/acm.2009.0510]

Smeeding et al., 2011x

Smeeding S, Bradshaw DH, Kumpfer KL, Trevithick S, Stoddard GJ. Outcome evaluation of the Veterans Affairs Salt Lake City Integrative Health Clinic for chronic nonmalignant pain. The Clinical Journal of Pain 2011;27(2):146-55.

Taketa et al., 2018x

Taketa PS, Landim ACLP, Teixeira MP, Cardoso JR, Moreira ECH, Facci LM. Effects of aquatic physiotherapy in a patient with youth ankilosant spondilitis: Case report [Efeitos da fisioterapia aquatica em um paciente com espondilite anquilosante juvenil: Relato de caso]. In: Fórum Internacional de Qualidade de Vida e Saúde Curitiba: Revista Científica JOPEF. Vol. 1. 2018.

Tanoue et al., 2009x

Tanoue MB, Reis PCd, Peternella FMN. The hydrotherapy as physiotherapeutic conduct in treatment of fibromyalgia: case report [A hidroterapia como conduta fisioterapêutica no tratamento da fibromialgia: relato de caso]. Revista Uninga 2009;19:161-171.

Tonieto et al., 2015x

Tonieto M, Rama P, Schuster RC, Renosto A. Effects of an Aquatic Physical Therapy Intervention in Patients After Stroke [Efeitos de uma intervenção de fisioterapia aquática em pacientes pós-acidente vascular cerebral]. Revista de Atenção à Saúde 2015;13(45):5-12. [DOI: 10.13037/rbcs.vol13n45.2838]

Tüfekçioğlu 2009x

Tüfekçioğlu E, Çotuk HB. Comparison of Heart Rate Variability in Differen Body Positions on Land and in Water [Suda ve karadakī farkli beden konumlarinda kalp hizih değişkenlīğīnīn karşilaştirilmasi]. Nigde University Journal of Physical Education and Sport Sciences 2009;3(3):152-9.

Useros-Olmo & Collado-Vázquez, 2010x

Useros-Olmo AI, Collado-Vázquez S. Effects of an hidrotherapy program in the treatment of cervical dystonia. A pilot study [Efectos de un programa de hidroterapia en el tratamiento de la distonía cervical. Estudio piloto]. Revista de Neurología 2010;51(11):669-76.

Useros-Olmo et al., 2018x

Useros-Olmo AI, Martinez-Pernia D, Huepe D. The effects of a relaxation program featuring aquatic therapy and autogenic training among people with cervical dystonia (a pilot study). Physiother Theory Pract 2018;1-10. [DOI: 10.1080/09593985.2018.1488319]

Vogtle et al., 1998x

Vogtle LK, Morris DM, Denton BG. An aquatic program for adults with cerebral palsy living in group homes. Physical Therapy Case Reports 1998;1(5):250-259.

Zanella 2011x

Zanella J, Romero CH. Quality of Life in Patients With Spinal Chord Injury Submitted to Neurofunctional Physiotherapy and Aquatic Physiotherapy [Qualidade de vida em pacientes com lesão medular submetidos a fisioterapia neurofuncional e fisioterapia aquática]. FIEP Bulletin Online, Special Edition 2011;81(2).
