# Supplementary material for: Applications, indications, and effects of passive hydrotherapy WATSU (WaterShiatsu)—A systematic review and meta-analysis
Source: PLoS One. 2020 Mar 13;15(3):e0229705. doi: 10.1371/journal.pone.0229705 (PMC7069616; doi:10.1371/journal.pone.0229705)
Supplement: S5 File — (PDF) [file pone.0229705.s010.pdf]

DATABASES (2018, without updates)

	retrieval with filters	Provider	Database		retrieval without filters	
		1	1	0	World Health Organization’s International Clinical Trials Registry Platform (ICTRP)	
		1	1	0	Cochrane Central Register of Controlled Trials (CENTRAL)	
		1	1	0	EU Clinical Trials Register	
		1	1	0	U.S. National Institutes of Health’s Clinical Trials Register	
		1	1	0	German Clinical Trials Register	
						includes:
						• LWW (Lippincott Williams & Wilkins): 0,
						• PsycARTICLES: 0,
					Ovid	• Embase 10,
						• MEDLINE: 4,
						• Books@Ovid: 1,
		1	6	19		• Journals@ovid: 2
		1	1	360	Swissbib (redundant)	
		1	1	43	Science direct	
17	embase	1	1	17	Embase	
						includes:
						• CINAHL: 19 Results
						• SocINDEX: 0
						• SPORTDiscus with Full Text: 30 Results
						• Business Source Premier: 16 Results
						• Regional Business News: 8 Results
						• MEDLINE: 5 Results
						• RILM Abstracts of Music Literature (1967 to Present only): 1 R
						• International Bibliography of Theatre & Dance with Full Text: 2 R
						• Dentistry & Oral Sciences Source: 4 Results
86	EBSCO	1	5	86	EBSCO	• Avery Index to Architectural Periodicals: 1 Result
12	waba	1	1	12	waba	
						includes:
						• Web of Science TM Core Collection: 2 Results
						• KCI-Korean Journal Database: 3 Results
						• SciELO Citation Index: 2 Results
12	Web of Science	1	4	12	Web of Scienc	• MEDLINE: 5 Results
19	CINAHL	1	1	19	CINAHL	
12	Livio	1	1	12	Livivo/Medpilot	
1	Pedro	1	1	1	Pedro	
1	Cochrane	1	1	1	Cochrane Library	
						includes:
						• ProQuest Dissertations & Theses Global: 49
						• British Periodicals: 13
						• Periodicals Archive Online: 5
						• American Periodicals: 13
83	ProQuest	1	1	83	ProQuest	• Periodicals Index Online: 3
14	Pubmed	1	1	14	Pubmed	
26	Researchgate	1	1	26	Researchgate	
13	alt: GS "wasser shiatsu"	1	1	16	GS "wasser shiatsu"	
23	alt: GS wassershiatsu			28	GS wassershiatsu	
800	alt: GS WATSU OR "water shiatsu" OR watershiatsu"			110	GS "water shiatsu"	
				120	GS watershiatsu	
				1000	GS watsu (of approximately 1'170)	
1119	merged EndNote "old"	20	32	1979	merged EndNote "new"	
