# Supplementary material for: Applications, indications, and effects of passive hydrotherapy WATSU (WaterShiatsu)—A systematic review and meta-analysis
Source: PLoS One. 2020 Mar 13;15(3):e0229705. doi: 10.1371/journal.pone.0229705 (PMC7069616; doi:10.1371/journal.pone.0229705)
Supplement: S6 File — (PDF) [file pone.0229705.s011.pdf]

A systematic review and meta-analysis of clinical effects and indications of passive hydrotherapy WATSU (water shiatsu)

Agnes M. Schitter, Johannes Fleckenstein, Peter Frei, Jan Taeymans, Nico Kurpiers, Lorenz Radlinger

Citation

Agnes M. Schitter, Johannes Fleckenstein, Peter Frei, Jan Taeymans, Nico Kurpiers, Lorenz Radlinger. A systematic review and meta-analysis of clinical effects and indications of passive hydrotherapy WATSU (water shiatsu). PROSPERO 2016 CRD42016029347 Available from: http://www.crd.york.ac.uk/PROSPERO/display_record.php?ID=CRD42016029347

Review question

Which effects are ascribed to WATSU?
What effect-sizes were reported with regard to WATSU?
Which indications for WATSU are described in the literature?

Searches

There will be no language restrictions. Since WATSU was developed in the 1980s, the publication period is restricted from 1979 on.
CINAHL, Cochrane Central Register of Controlled Trials (CENTRAL), EBSCO (Dentistry & Oral Sciences Source, MEDLINE, SocINDEX, SPORTDiscus), Embase, EU Clinical Trials Register, German Clinical Trails Register, Google Scholar, LIVIVO (MEDPILOT), Ovid (Embase, Books@ovid, Journals@ovid, LWW, MEDLINE, PsycARTICLES), Physiotherapy Evidence Database (PEDro), ProQuest (ProQuest Dissertations & Theses Global, American Periodicals, British Periodicals, Periodicals Archive Online, Periodicals Index Online), PubMed, ResearchGate, ScienceDirect, The Cochrane Library, U.S. National Institutes of Health's Clinical Trials Register, the homepage of WABA (Worldwide Aquatic Bodywork Association), Web of Science (Web of Science TM Core Collection, Korean Journal Database, SciELO Citation Index), and World Health Organization's International Clinical Trials Registry Platform (ICTRP).
A manual search of the reference lists of retrieved publications will be conducted.
For additional grey literature, Institutes of Aquatic Bodywork and authors of recent publications on WATSU will be contacted.
The searches will be re-run just before the final analysis and further studies retrieved for inclusion.

Types of study to be included

No restriction concerning types of study will be made.

Condition or domain being studied

All conditions that are being treated with WATSU will be included in this review.

Participants/population

Publications involving treatment of humans with WATSU at all ages and all states of health will be considered.

Intervention(s), exposure(s)

The intervention WATSU (water shiatsu) is a passive hydrotherapy that is administered individually in 35°C warm water.

Comparator(s)/control

This is the first systematic review about WATSU and will allow for a broad spectrum of information. The search therefore will not be limited to publications that report on controlled trials.

Primary outcome(s)

Described observations of effects, and reported indications of WATSU.

Secondary outcome(s)

Reported effect sizes on the intervention WATSU.

Data extraction (selection and coding)

Publication retrieved employing the search strategy described above will be collected, and duplicates identified and removed.
Then titles and abstracts will be screened independently by two persons to decide upon their potential to meet the inclusion criteria. The full text of these potentially eligible studies will be retrieved and independently assessed for eligibility by two review team members. Disagreements between them over the eligibility of particular studies will be resolved through discussion and by consensus (with a third reviewer, where necessary).
Data extraction will be performed using a customized data extraction form, that will include study design, subjects' characteristics, measurement method, outcome. Two review authors will extract data independently. Discrepancies will be identified and resolved through discussion (with a third author where necessary). Missing data will be requested from study authors.

Risk of bias (quality) assessment

The methodological quality of the included articles will be rated by two authors independently, using “Cochrane Collaboration’s tool for assessing risk of bias“. in a second step, quality of evidence and grade of recommendation will be assed employing GRADEpro. Disagreements between the review authors over the risk of bias in particular studies will be resolved by discussion, with involvement of a third review author where necessary.

Strategy for data synthesis

Findings from the retrieved studies will be summarized and synthesized. If sufficiently homogenous, data will be synthesized quantitatively in a meta-analysis.

Analysis of subgroups or subsets

A subgroup-analysis will be considered depending on the data retrieved.

Contact details for further information

Agnes M. Schitter
agnes.schitter@ikom.unibe.ch

Organisational affiliation of the review

University of Bern
http://www.unibe.ch/

Review team members and their organisational affiliations

Ms Agnes M. Schitter. University of Bern - Institute of Complementary Medicine, Switzerland; University of Hildesheim - Institute of Sport Science, Germany
Dr Johannes Fleckenstein. University of Bern - Institute of Complementary Medicine, Switzerland; Goethe-University Frankfurt - Department of Sports Medicine, Institute of Sports Sciences, Germany
Professor Peter Frei. University of Hildesheim - Institute of Sport Science, Germany
Professor Jan Taeymans. Bern University of Applied Sciences - Health, Switzerland; Vrije Universiteit Brussel - Sports- and Rehabilitation Sciences, Belgium
Professor Nico Kurpiers. University of Hildesheim - Institute of Sport Science, Germany
Professor Lorenz Radlinger. Bern University of Applied Sciences - Health, Switzerland

Anticipated or actual start date

21 January 2016

Anticipated completion date

16 May 2017

Funding sources/sponsors

This systematic-review is part of a dissertation which is supervised in collaboration by members of University of Hildesheim, Bern University of Applied Sciences, and University of Bern.

Conflicts of interest

Agnes M. Schitter was chairwoman of the Swiss Aquatic Bodyworkers' Association, NAKA (until June 2016), and member of the supervisory board of the Institute for Aquatic Bodywork IAKA AG, Bern, Switzerland (until March 2016). Both engagements were honorary.

Language

English

Country

Switzerland

Stage of review

Review_Completed_not_published

Subject index terms status

Subject indexing assigned by CRD

Subject index terms

Acupressure; Humans; Hydrotherapy; Musculoskeletal Manipulations; Water

Date of registration in PROSPERO

09 May 2016

Date of publication of this version

02 June 2017

Revision note for this version

Changed review team, conflicts, searches, and status of review.

Details of any existing review of the same topic by the same authors

Stage of review at time of this submission

**Stage**	**Started**	**Completed**
Preliminary searches	Yes	Yes
Piloting of the study selection process	Yes	Yes
Formal screening of search results against eligibility criteria	Yes	Yes
Data extraction	Yes	Yes
Risk of bias (quality) assessment	Yes	Yes
Data analysis	Yes	Yes

Revision note

Changed review team, conflicts, searches, and status of review.

Versions

09 May 2016
02 June 2017

PROSPERO
This information has been provided by the named contact for this review. CRD has accepted this information in good faith and registered the review in PROSPERO. CRD bears no responsibility or liability for the content of this registration record, any associated files or external websites.
