# Supplementary material for: Applications, indications, and effects of passive hydrotherapy WATSU (WaterShiatsu)—A systematic review and meta-analysis
Source: PLoS One. 2020 Mar 13;15(3):e0229705. doi: 10.1371/journal.pone.0229705 (PMC7069616; doi:10.1371/journal.pone.0229705)
Supplement: S7 File — (PDF) [file pone.0229705.s012.pdf]

Study Flow

RETRIEVAL	total	in	out
retrieval with filters		1'119	
retrieval without filters		1'979	
	3'098		
alert 1 = alert 1 add (additional sources)			
alert 2		60	
alert 3		27	
alert 4		6	
alert 5		28	
alert 6 (last search in Google Scholar 3.10.2019)		246	
alert 7 (last search in all databases 3.10.2019)		293	
	660		
all Databases	**3'758**		
additional items (institutes, handsearch "alert 1 add")		77	
additional items (institutes, researchers "alert 6 add")		13	
additional items total	**90**		
**TOTALLY RETRIEVED**	**3'848**		

SCREENINGS	total	in	out
1. Rnd screening, no duplicates	1'235	**738**	-497
2. Rnd general	738	715	-23
2. Rnd alert 1 add	77	77	0
2. Rnd alert 2	60	17	-43
2. Rnd alert 3	27	17	-10
2. Rnd alert 4	6	4	-2
2. Rnd alert 5	28	22	-6
2. Rnd alert 6 incl. Add	259	141	-118
2. Rnd alert 7 without duplicates	214	28	-186
	**1'409**	**1'021**	**-388**
**TOTALLY SCREENED,** without duplicates	**1'906**		**-885 Screened out**
**SCREENED IN (=assessed)**	**1'021**		

ASSESSMENT and DATA EXTRACTION

14 no full text

357 excluded during assessment
31 excluded during extraction

388 duplicates, not concerning WATSU, or only in itemization

131 Miscellaneous (e.g. magazine articles)
38 Conference proceedings
156 Theses
99 Books / chapters
424 Grey literature

109 Secondary peeer
59 Primary peer (WATSU only minor part)

27 Primary peer RoB
195 peer reviewed

Included, evaluated for risk of bias **27**

994 excluded
